# Trifluoromethylthiolation of Arenes Using Lewis Acid and Lewis Base Dual Catalysis

**DOI:** 10.1021/acs.joc.3c02571

**Published:** 2023-12-29

**Authors:** Lachlan J. N. Waddell, Claire Wilson, Andrew Sutherland

**Affiliations:** School of Chemistry, The Joseph Black Building, University of Glasgow, Glasgow G12 8QQ, U.K.

## Abstract

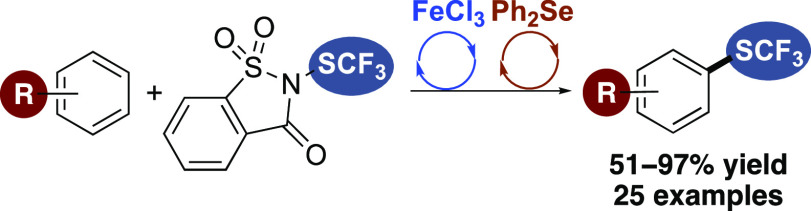

Incorporation of the highly lipophilic trifluoromethanesulfenyl group into bioactive molecules facilitates transport through lipid membranes, and thus, CF_3_S-containing compounds are important for drug discovery. Although reagents and procedures have been reported for arene trifluoromethylthiolation, methods are still required that are applicable to a diverse substrate scope and can be performed under mild conditions. Here, we describe a rapid and efficient approach for the trifluoromethylthiolation of arenes by catalytic activation of *N*-trifluoromethylthiosaccharin using a combination of iron(III) chloride and diphenyl selenide. This dual catalytic process allowed regioselective functionalization of a wide range of arenes and *N*-heterocycles under mild conditions and was used for the trifluoromethylthiolation of bioactive compounds such as tyrosine and estradiol.

## Introduction

**Figure 1:**
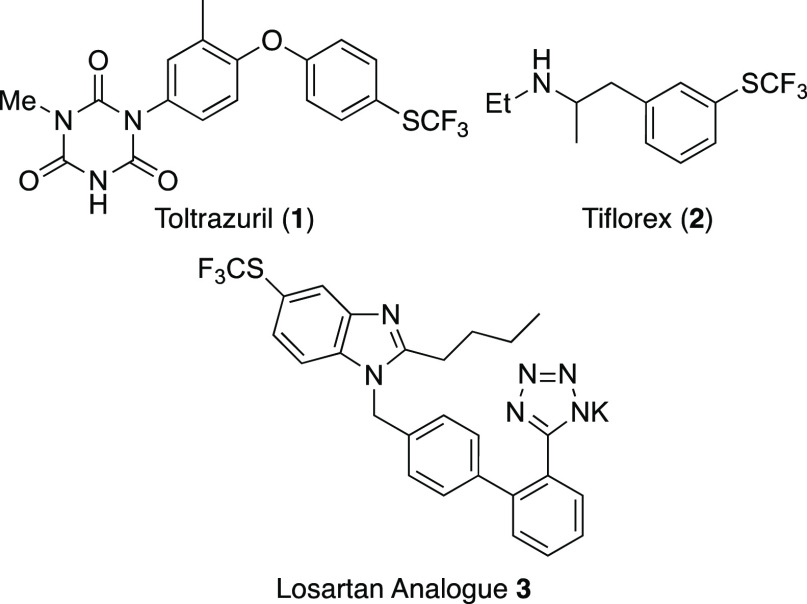
Trifluoromethanesulfenyl-containing bioactive compounds.

The trifluoromethanesulfenyl (CF_3_S) motif is a privileged functional group for the design of bioactive compounds.^1^ This is due to the high lipophilicity and high Hansch parameter (1.44) of this group that results in facile transport of CF_3_S-containing compounds through lipid membranes, thus enhancing bioavailabilty.^2^ Consequently, the trifluoromethanesulfenyl group is a valuable motif for agrochemical and pharmaceutical discovery. Several bioactive CF_3_S-containing compounds are commercially available and include toltrazuril (**1**),^3^ used to treat coccidiosis in livestock and poultry, and the anorectic drug, tiflorex (**2**) (Figure 1).^4^ Trifluoromethanesulfenyl analogues of losartan (e.g., **3**) have also been developed as potential hypotensive agents.^5^

**Figure 2:**
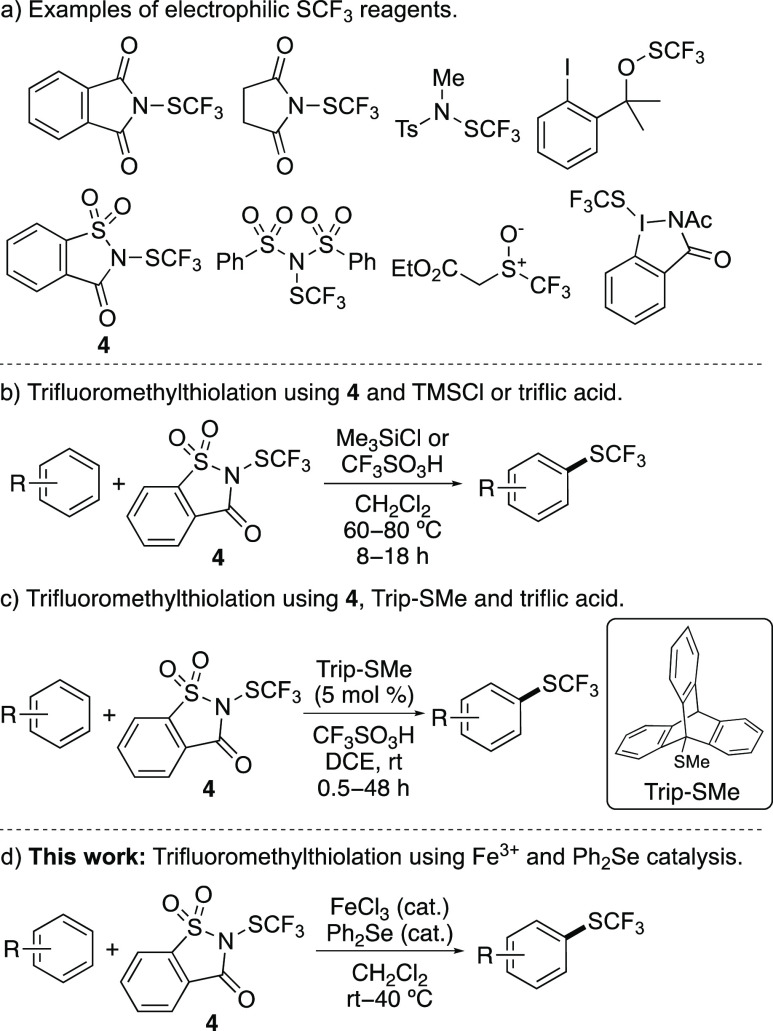
Reagents and methods for the trifluoromethylthiolation of arenes.

The importance of trifluoromethanesulfenyl-containing arenes has led to the development of a wide range of methods for the incorporation of this motif.^6^ A direct approach is the trifluoromethylthiolation of arene C–H bonds using electrophilic reagents. Since the development of the first electrophilic reagent, trifluoromethylsulfenyl chloride,^7^ which is gaseous and toxic, a wide range of more readily available, bench-stable CF_3_S-transfer reagents have been reported for trifluoromethylthiolation of arenes (Figure 2a).^8−15^ Although many of these reagents are effective, methods involving *N*-trifluoromethylthiosaccharin (**4**) have been widely used for (hetero)arene trifluoromethylthiolation.^16,17^ The synthesis and application of *N*-trifluoromethylthiosaccharin (**4**) was first reported by Shen and co-workers, who demonstrated efficient trifluoromethylthiolation of *N*-heterocycles and electron-rich arenes using either trimethylsilyl chloride or triflic acid as an activator (Figure 2b).^12b^ Since this first report, modifications to accelerate the reaction or lower the temperature have been described using Lewis base catalysis^18^ or trifluoroethanol as a solvent.^19^ More recently, the Miura group showed that activation of *N*-trifluoromethylthiosaccharin (**4**) using a combination of triptycenyl sulfide (Trip-SMe) and triflic acid allowed room temperature trifluoromethylthiolation, via a sulfonium intermediate (Figure 2c).^20^

We have shown that iron(III) salts are effective Lewis acids for the activation of succinimide and saccharin-based reagents and the subsequent regioselective functionalization of arenes.^21−23^ During our work on the thiocyanation of arenes,^23^ trifluoromethylsulfenyl compounds were prepared in two steps by iron(III)-catalyzed thiocyanation, followed by the reaction with the Ruppert–Prakash reagent under basic conditions.^24,25^ To circumvent a two-step approach, we proposed that iron(III)-catalysis could be used to activate CF_3_S-based electrophiles, for the direct, single-step trifluoromethylthiolation of arenes. In 2015, Li and co-workers reported the trifluoromethylthiolation of electron-rich arenes using iron(III) chloride activation of *N*-trifluoromethylthiosaccharin (**4**).^26^ For the functionalization of benzene-based arenes, silver hexafluoroantimonate (30 mol %) was used as an additive and the reactions were performed at 100 °C over a 16 h reaction time. Based on our recent work of Lewis base-accelerated, iron(III)-catalyzed arene thiolation reactions,^22^ we believed that a faster and milder trifluoromethylthiolation procedure could be developed, that would be amenable to a wider substrate scope, and, in particular, bioactive compounds for drug discovery. Here, we report the rapid trifluoromethylthiolation of arenes and heteroarenes using a dual catalytic method involving iron(III) chloride and diphenyl selenide activation of *N*-trifluoromethylthiosaccharin (**4**) (Figure 2d). In addition to demonstrating room temperature reactions, using low catalyst loadings (2.5–5 mol %), we also describe the application of this method for the effective trifluoromethylthiolation of various bioactive molecules.

## Results and Discussion

An iron(III)-catalyzed trifluoromethylthiolation was optimized using 2-methylanisole (**5a**) and *N*-trifluoromethylthiosaccharin (**4**) (Table 1).^27^ Initially, trifluoromethylation was attempted by activation of **4** using iron(III) chloride at a temperature of 40 °C (entry 1). After a reaction time of 20 h, negligible conversion was observed. Similar results were obtained using strong Lewis acids such as iron(III) triflimide, silver(I) triflimide, and aluminum(III) chloride (entries 2–4). In an attempt to accelerate the reaction, the Lewis base, diphenyl selenide (10 mol %) was added to an iron(III) chloride-catalyzed reaction (entry 5).^28^ At the same temperature of 40 °C, the reaction reached full conversion after 0.1 h. This led to the isolation of **6a** as a single regioisomer in 97% yield. Further optimization demonstrated that similar results could be obtained by conducting the reaction at room temperature (entry 6). The loading for both catalysts could also be lowered to 2.5 mol % (entry 7) and while this required a 2 h reaction time, the yield of **6a** was maintained. Finally, to show that both catalysts were required for the activation of *N*-trifluoromethylthiosaccharin (**4**), the reaction was repeated in the absence of iron(III) chloride (entry 8). After a 20 h reaction time under the optimized conditions, this showed no conversion, confirming the role of both catalysts in the activation of *N*-trifluoromethylthiosaccharin (**4**).

**Table 1:**
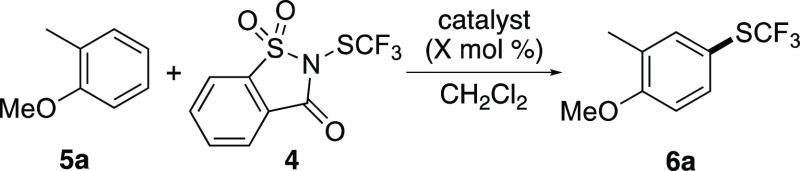
Optimization Studies for Trifluoromethylthiolation of 2-Methylanisole (**5a**)

entry	catalyst	catalyst loading (mol %)	temperature (°C)	time (h)	yield (%)^a^
1	FeCl_3_	10	40	20	3^b^
2^*c*^	Fe(NTf_2_)_3_	10	40	20	0
3	AgNTf_2_	10	40	20	0
4	AlCl_3_	10	40	20	0
5	FeCl_3_ + Ph_2_Se	10	40	0.1	97
6	FeCl_3_ + Ph_2_Se	10	rt	0.1	94
7	FeCl_3_ + Ph_2_Se	2.5	rt	2	94
8	Ph_2_Se	2.5	rt	20	0

a Isolated yields.

b Conversion.

c Fe(NTf_2_)_3_ was prepared in situ from FeCl_3_ (10 mol %) and [BMIM]NTf_2_ (30 mol %).

**Scheme 1:**
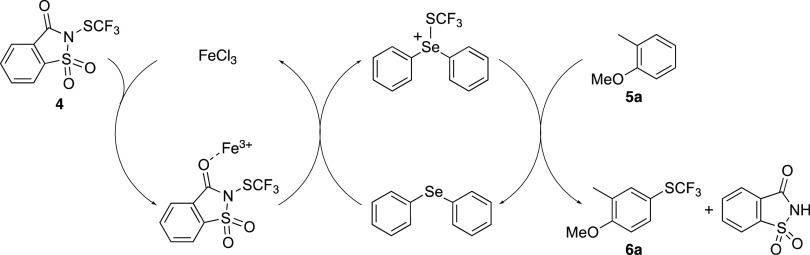
Proposed Mechanism of Trifluoromethylthiolation of 2-Methylanisole (**5a**)

Based on the reactions performed using only iron(III) chloride or diphenyl selenide (Table 1, entries 1 and 8), which show that the Lewis acid and Lewis base catalysts are both required for reagent activation, a mechanism has been proposed (Scheme 1). Following activation of *N*-trifluoromethylthiosaccharin (**4**) by the Lewis acidic iron(III) ion, the resulting species then undergoes a fast substitution reaction with the Lewis base, diphenyl selenide, yielding a trifluoromethylated selenium cation. As the selenium cation is significantly more reactive that the iron-activated saccharin intermediate, this can perform a much faster trifluoromethylation of 2-methylanisole (**5a**) under milder conditions, yielding the product and regenerating the Lewis base catalyst. For most reactions conducted in this study, only a single regioisomer was observed (e.g., **6a**), and in general, this gave the *para*-product in relation to the strongest electron-donating group. We believe that the highly regioselective nature of this transformation is due to the steric bulk of the proposed trifluoromethylthiolated diphenyl selenium cation.

**Scheme 2:**
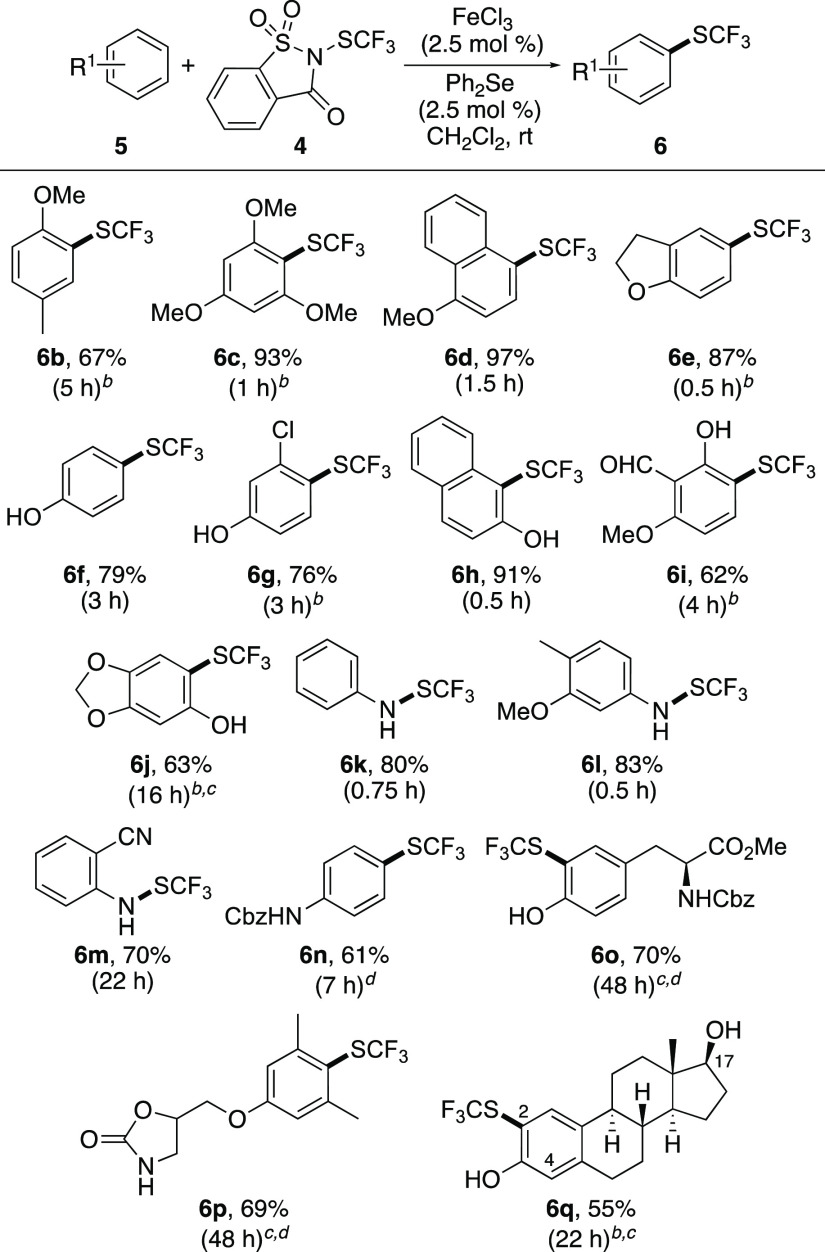
Reaction Scope of Dual-Catalytic Trifluoromethylthiolation of Arenes Isolated yields. Reaction done using FeCl_3_ (5 mol %) and Ph_2_Se (5 mol %). Reaction done at 40 °C. Reaction done using FeCl_3_ (10 mol %) and Ph_2_Se (10 mol %).

The scope of the dual-catalytic trifluoromethylthiolation reaction was then explored (Scheme 2).^29^ For anisole and phenol substrates (**5b**–**5i**), the method was found to be fast and efficient, allowing regioselective trifluoromethylthiolation of compounds with a range of substitution patterns. For the majority of substrates, a 2.5 mol% catalyst loading was sufficient, while a slightly higher loading (5 mol %) was required for some of the *ortho*-substituted substrates (e.g., **6b**, **6c**, and **6g**). The benefit of the dual catalytic process is evident from the result observed for 1,3,5-trimethoxy-4-(trifluoromethylthio)benzene (**6c**). While the previously reported FeCl_3_ (10 mol %)/AgSbF_6_ (30 mol %) method gave **6c** in 52% yield after a 16 h reaction and at 100 °C,^26^ the dual-catalytic method was complete after 1 h at room temperature and gave **6c** in 93% yield. It should be noted that to determine the regiochemical outcome of reaction with 6-methoxysalicyaldehyde (**5i**), X-ray crystallography was required.^30^ This showed that the 3-trifluoromethylthioloated isomer **6i** was the sole product, with the reaction taking place *para* to the MeO group. The regioselective outcome of this reaction is likely due to a directing effect between the adjacent hydroxyl group and the trifluoromethylthiolated diphenyl selenium cation. We have previously observed electrophilic aromatic halogenation reactions where the regiochemical outcome can be controlled by a directing group.^21a^ For unprotected anilines (**5k**–**5m**), the only product observed was that of *N*-trifluoromethylthiolation.^31^ For activated anilines **5k** and **5l**, this gave the *N*-substituted products, **6k** and **6l** in short reaction times and high yields. For the more deactivated substrate, 2-aminobenzonitrile **5m**, a longer reaction time (22 h) was required. The reactivity of anilines could be switched using *N*-protected derivatives. For example, trifluoromethylthiolation of *N*-Cbz-protected aniline **5n** gave only the ring-substituted product. Although a slightly higher loading of both catalysts was required (10 mol %), *para*-substituted product **6n** was formed as the sole product in 61% yield. Following exploration of the reaction scope with simple arenes, the study then focused on the application of this methodology for the trifluoromethylthiolation of more complex, bioactive compounds. In particular, challenging *ortho*-substituted targets, tyrosine derivative **5o**, the pain relief drug metaxalone (**5p**),^32^ and β-estradiol (**5q**), the estrogen steroid hormone, were chosen. As expected, higher catalyst loadings, a slightly higher temperature (40 °C), and longer reaction times were required. For tyrosine derivative **5o** and metaxalone (**5p**), a 10 mol% catalyst loading allowed completion after 48 h and the isolation of the trifluoromethylthiolation products, **6o** and **6p,** in 70% and 69% yields, respectively. Our previous studies on C–S bond forming reactions of estradiol (**5q**) with electrophilic succinimide- or saccharin-based reagents required protection of the hydroxyl groups.^22b,23^ However, reaction of estradiol (**5q**) using the dual-catalytic method was found to proceed without the requirement of protecting groups. The optimal conditions involved a 5 mol % catalyst loading, which gave a 2:1 mixture of the 2- and 4-trifluoromethylthiolated products. Following column chromatography, the major 2-isomer **6q** was isolated in 55% yield.

**Scheme 3:**
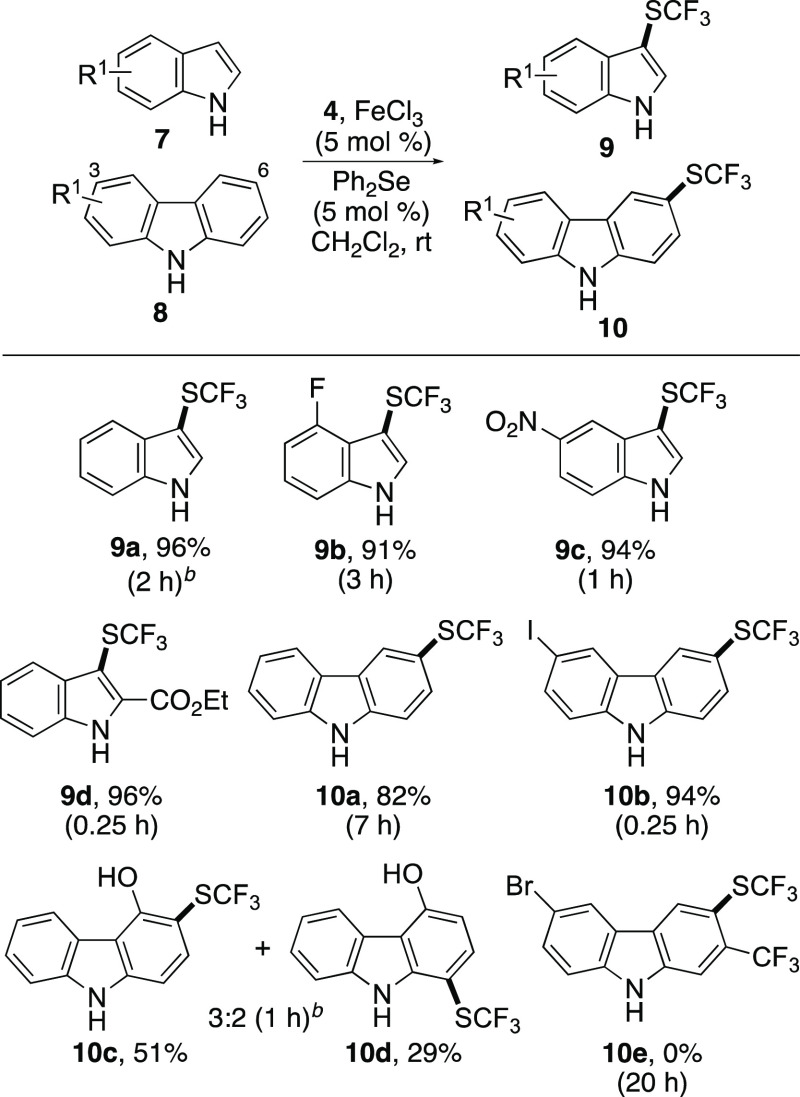
Reaction Scope of Dual-Catalytic Trifluoromethylthiolation of *N*-Heteroarenes Isolated yields. Reaction done using FeCl_3_ (2.5 mol %) and Ph_2_Se (2.5 mol %).

Following the development of the dual-catalytic method for the trifluoromethylthiolation of benzene derivatives, the study then focused on functionalization of *N*-heterocycles. For all *N*-heterocycles investigated, trifluoromethylthiolation was found to proceed at room temperature using either 2.5 or 5 mol % catalyst loading (Scheme 3). Reaction of indole (**7a**) and 2-, 4-, or 5-substituted analogues (**7b**–**7d**) was found to proceed under short reaction times (0.25–3 h) and gave 3-trifluoromethylthiolated indoles **9a**–**9d** in excellent yields (91–96%).^33^ Again, use of the dual catalytic method for trifluoromethylthiolation of indoles was significantly faster under milder conditions than the previous iron(III)/silver(I) process (50 °C, 16 h).^26^

Recently, Jiang and co-workers highlighted the importance of thiolated carbazoles for a variety of applications and reported a direct 3,6-dithiolation reaction using AgSbF_6_ (30 mol %), potassium persulfate and diaryl disulfides as the sulfur source.^34^ To complement this study, we were interested to discover whether our dual catalytic method could be used for the selective preparation of monothioarylated products (Scheme 3). The reaction of carbazole (**8a**) with *N*-trifluoromethylthiosaccharin (**4**) using a 5 mol % catalyst loading and at room temperature required a reaction time of 7 h. Only one product, 3-trifluoromethylthiolated isomer **10a,** was observed by ^1^H NMR spectroscopy, which was isolated in 82% yield. Using substituted carbazoles, the reaction was found to be selective for the most activated ring. The reaction of 3-iodocarbazole (**8b**) was complete after 15 min and gave 6-trifluoromethylthio isomer **10b** as the sole product in 94% yield. This reaction was used to investigate the scalability of the transformation. On a 1 mmol scale, the reaction was complete at the same time (0.25 h) and a similar yield (93%). Highly activated carbazoles with competing directing groups to the nitrogen atom gave mixtures of isomers. The reaction of 4-hydroxycarbazole **8c** was complete after 1 h and gave a 3:2 ratio of the 3- and 1-trifluoromethylthiolated isomers. These were readily separated by column chromatography to give trifluoromethylthiolated products **10c** and **10d**, in 51 and 29% yield, respectively. The limitation of this method was found using highly deactivated carbazoles, which showed no reaction. For example, attempted synthesis of **10e**, using a carbazole bearing deactivating substituents attached to both rings, showed no trifluoromethylthiolation after 20 h.

**Scheme 4:**
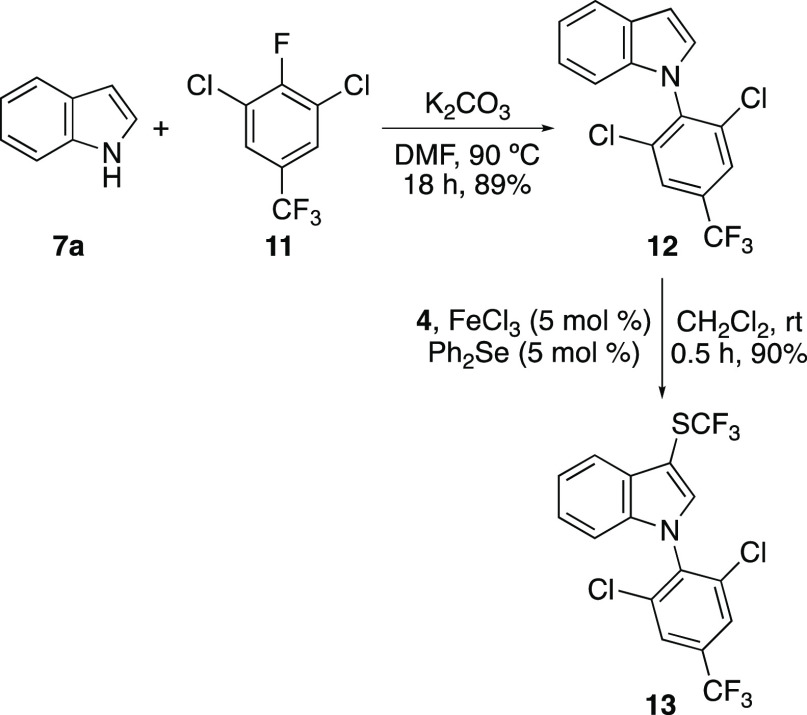
Synthetic Application of Dual-Catalytic Trifluoromethylthiolation Reaction Isolated yields.

The final stage of the study investigated the application of the dual catalytic trifluoromethylthiolation reaction for the synthesis of a bioactive target. The target chosen was *N*-substituted 3-trifluoromethylthioindole **13**, which has potent insecticidal activity against parasitic acarians of animals.^35^ In addition to exhibiting fast acting properties, the insecticide has low toxicity to mammals. A two-step synthesis of **13** was devised involving synthesis of the *N*-aryl indole **12**, followed by the dual catalytic trifluoromethylthiolation reaction. *N*-Aryl indole **12** was prepared using a nucleophilic aromatic substitution reaction of indole (**7a**) with commercially available 1,3-dichloro-2-fluoro-5-(trifluoromethyl)benzene (**11**) (Scheme 4). Under basic conditions, this gave **12** in 89% yield. Trifluoromethylthiolation of **12** using the dual catalytic process was then investigated. Despite the deactivating *N*-aryl group, the reaction of indole **12** with *N*-trifluoromethylthiosaccharin (**4**) was found to proceed at room temperature and was complete after 30 min to give insecticide **13** in 90% yield.

## Conclusions

In summary, a dual catalytic method involving Lewis acid and Lewis base activation of *N*-trifluoromethylthiosaccharin (**4**) for the regioselective trifluoromethylthiolation of arenes has been developed. The combination of iron(III) chloride and diphenyl selenide allowed fast and efficient functionalization of anisoles, phenols, *N*-protected anilines, as well as *N*-heterocycles such as indoles and carbazoles. For the majority of substrates, the reaction was found to proceed using low catalyst loadings (2.5–5 mol %) and under mild conditions (rt–40 °C). The method was also applied for the trifluoromethylthiolation of more complex, bioactive compounds such as tyrosine, metaxalone, and estradiol, and used as the key step for the efficient synthesis of a potent insecticide. The development of further applications of Lewis acid and Lewis base dual catalytic functionalization of arenes is underway.

## Experimental Section

All reagents and starting materials were obtained from commercial sources and used as received. *N*-(Trifluoromethylthio)saccharin (**4**) was prepared according to the literature.^16^ All reactions performed at elevated temperatures were heated using an oil bath. Brine refers to a saturated aqueous solution of sodium chloride. Flash column chromatography was performed using silica gel 60 (40–63 μm). Aluminum-backed plates precoated with silica gel 60F_254_ were used for thin layer chromatography and were visualized with a UV lamp or by staining with potassium permanganate. ^1^H NMR spectra were recorded on a NMR spectrometer at 400 MHz and data are reported as follows: chemical shift in ppm relative to tetramethylsilane or the to the solvent as internal standard (CHCl_3_, δ 7.26 ppm), multiplicity (s = singlet, d = doublet, t = triplet, q = quartet, m = multiplet or overlap of nonequivalent resonances, integration). The abbreviation br s refers to broad singlet. ^13^C NMR spectra were recorded on a NMR spectrometer at 101 MHz and data are reported as follows: chemical shift in ppm relative to tetramethylsilane or the solvent as internal standard (CDCl_3_, δ 77.0 ppm), multiplicity with respect to hydrogen (deduced from DEPT experiments, C, CH, CH_2_ or CH_3_). ^19^F NMR spectra were recorded on an NMR spectrometer at 376 MHz using CDCl_3_ as the solvent and data are reported as follows: chemical shift in ppm, multiplicity (s = singlet, t = triplet, m = multiplet). Infrared spectra were recorded on a FTIR spectrometer; wavenumbers are indicated in cm^–1^. Mass spectra were recorded using electrospray (ESI) or atmospheric pressure chemical ionization (APCI) techniques on a quadrupole time-of-flight (Q-TOF) mass spectrometer. Melting points are uncorrected. Optical rotations were determined as solutions irradiating with the sodium D line (λ = 589 nm) using a polarimeter. [α]_D_ values are given in units 10^–1^ deg cm^–1^ g^–1^.

### 2-Methyl-4-(trifluoromethylthio)anisole (**6a**)^36^

To a solution of *N*-(trifluoromethylthio)saccharin (**4**) (0.0500 g, 0.177 mmol) and iron(III) chloride (0.000650 g, 0.00400 mmol, 2.5 mol %) in dry dichloromethane (1 mL) under argon were added 2-methylanisole (**5a**) (0.0198 mL, 0.160 mmol) and diphenyl selenide (0.000700 mL, 0.00400 mmol, 2.5 mol %). The reaction mixture was stirred at room temperature in the absence of light for 2 h. The reaction mixture was then diluted with dichloromethane (10 mL) and washed with water (10 mL). The aqueous layer was extracted with dichloromethane (2 × 10 mL) and the combined organic layers were washed with brine (20 mL). The organic phase was dried (MgSO_4_), filtered and concentrated in vacuo. Purification by flash column chromatography (pentane) gave 2-methyl-4-(trifluoromethylthio)anisole (**6a**) (0.0336 g, 94%) as a colorless oil. Spectroscopic data were consistent with the literature.^36^^1^H NMR (400 MHz, CDCl_3_) δ 7.47 (dd, *J* = 8.5 Hz, 2.3 Hz, 1H), 7.41 (d, *J* = 2.3 Hz, 1H), 6.84 (d, *J* = 8.5 Hz, 1H), 3.86 (s, 3H), 2.22 (s, 3H); ^13^C{^1^H} NMR (101 MHz, CDCl_3_) δ 160.2 (C), 138.8 (CH), 136.0 (CH), 129.9 (C, q, ^1^*J*_CF_ = 308.2 Hz), 128.3 (C), 114.3 (C, q, ^3^*J*_CF_ = 2.1 Hz), 110.7 (CH), 55.6 (CH_3_), 16.2 (CH_3_); MS (APCI) *m*/*z* 222 (M^+^, 100).

### 2-(Trifluoromethylthio)-4-methylanisole (**6b**)^36^

The reaction was performed according to the general procedure using 4-methylanisole (**5b**) (0.0202 mL, 0.160 mmol), *N*-(trifluoromethylthio)saccharin (**4**) (0.0500 g, 0.177 mmol), iron(III) chloride (0.00130 g, 0.00800 mmol, 5.0 mol %), and diphenyl selenide (0.00139 mL, 0.00800 mmol, 5.0 mol %). The reaction mixture was stirred at room temperature for 5 h. Purification by flash column chromatography (pentane) gave 2-(trifluoromethylthio)-4-methylanisole (**6b**) (0.0238 g, 67%) as a colorless oil. Spectroscopic data were consistent with the literature.^36^^1^H NMR (400 MHz, CDCl_3_) δ 7.42 (d, *J* = 2.1 Hz, 1H), 7.26–7.23 (m, 1H), 6.87 (d, *J* = 8.4 Hz, 1H), 3.88 (s, 3H), 2.30 (s, 3H); ^13^C{^1^H} NMR (101 MHz, CDCl_3_) δ 158.7 (C), 139.0 (CH), 133.5 (CH), 130.8 (C), 129.8 (C, q, ^1^*J*_CF_ = 308.8 Hz), 112.1 (C), 111.8 (CH), 56.3 (CH_3_), 20.3 (CH_3_); MS (APCI) *m*/*z* 222 (M^+^, 100).

### 1,3,5-Trimethoxy-4-(trifluoromethylthio)benzene (**6c**)^37^

The reaction was performed according to the general procedure using 1,3,5-trimethoxybenzene (**5c**) (0.0269 g, 0.160 mmol), *N*-(trifluoromethylthio)saccharin (**4**) (0.0462 g, 0.163 mmol), iron(III) chloride (0.00130 g, 0.00800 mmol, 5.0 mol %), and diphenyl selenide (0.00139 mL, 0.00800 mmol, 5.0 mol %). The reaction mixture was stirred at room temperature for 1 h. Purification by flash column chromatography (20% ethyl acetate in hexane) gave 1,3,5-trimethoxy-4-(trifluoromethylthio)benzene (**6c**) (0.0401 g, 93%) as a white solid. Mp 76–78 °C (lit.^37^ 76–77 °C); ^1^H NMR (400 MHz, CDCl_3_) δ 6.16 (s, 2H), 3.88 (s, 6H), 3.85 (s, 3H); ^13^C{^1^H} NMR (101 MHz, CDCl_3_) δ 164.6 (C), 163.6 (2 × C), 129.6 (C, q, ^1^*J*_CF_ = 310.7 Hz), 91.9 (C), 91.2 (2 × CH), 56.4 (2 × CH_3_), 55.6 (CH_3_); MS (APCI) *m*/*z* 269 (M + H^+^, 100).

### 1-Methoxy-4-(trifluoromethylthio)naphthalene (**6d**)^38^

The reaction was performed according to the general procedure using 1-methoxynaphthalene (**5d**) (0.0232 μL, 0.160 mmol), *N*-(trifluoromethylthio)saccharin (**4**) (0.0500 g, 0.177 mmol), iron(III) chloride (0.000650 g, 0.00400 mmol, 2.5 mol %), and diphenyl selenide (0.000700 mL, 0.00400 mmol, 2.5 mol %). The reaction mixture was stirred at room temperature for 1.5 h. Purification by flash column chromatography (hexane) gave 1-methoxy-4-(trifluoromethylthio)naphthalene (**6d**) (0.0403 g, 97%) as a colorless oil. Spectroscopic data were consistent with the literature.^38^^1^H NMR (400 MHz, CDCl_3_) δ 8.48 (br d, *J* = 8.4 Hz, 1H), 8.33 (dd, *J* = 8.4, 1.4 Hz, 1H), 7.90 (d, *J* = 8.1 Hz, 1H), 7.66 (ddd, *J* = 8.4, 6.8, 1.4 Hz, 1H), 7.56 (ddd, *J* = 8.4, 6.8, 1.2 Hz, 1H), 6.85 (d, *J* = 8.1 Hz, 1H), 4.05 (s, 3H); ^13^C{^1^H} NMR (101 MHz, CDCl_3_) δ 158.8 (C), 139.1 (CH), 136.3 (C), 129.9 (C, q, ^1^*J*_CF_ = 309.6 Hz), 128.2 (CH), 126.6 (C), 126.1 (CH), 125.9 (CH), 122.7 (CH), 112.4 (C, q, ^3^*J*_CF_ = 2.2 Hz), 104.0 (CH), 55.9 (CH_3_); MS (APCI) *m*/*z* 258 (M^+^, 100).

### 2,3-Dihydro-5-(trifluoromethylthio)benzofuran (**6e**)^39^

The reaction was performed according to the general procedure using 2,3-dihydrobenzofuran (**5e**) (0.0181 g, 0.160 mmol), *N*-(trifluoromethylthio)saccharin (**4**) (0.0500 g, 0.177 mmol), iron(III) chloride (0.00130 g, 0.00800 mmol, 5.0 mol %), and diphenyl selenide (0.00139 mL, 0.00800 mmol, 5.0 mol %). The reaction mixture was stirred at room temperature for 0.5 h. Purification by flash column chromatography (hexane–5% diethyl ether in hexane) gave 2,3-dihydro-5-(trifluoromethylthio)benzofuran (**6e**) (0.0306 g, 87%) as a colorless oil. Spectroscopic data were consistent with the literature.^39^^1^H NMR (400 MHz, CDCl_3_) δ 7.45 (br s, 1H), 7.41 (dd, *J* = 8.3, 1.7 Hz, 1H), 6.80 (d, *J* = 8.3 Hz, 1H), 4.64 (t, *J* = 8.8 Hz, 2H), 3.25 (t, *J* = 8.8 Hz, 2H); ^13^C{^1^H} NMR (101 MHz, CDCl_3_) δ 162.8 (C), 137.7 (CH), 133.6 (CH), 129.8 (C, q, ^1^*J*_CF_ = 308.2 Hz), 129.0 (C), 114.4 (C, q, ^3^*J*_CF_ = 2.1 Hz), 110.5 (CH), 72.1 (CH_2_), 29.4 (CH_2_); MS (APCI) *m*/*z* 220 (M^+^, 100).

### 4-(Trifluoromethylthio)phenol (**6f**)^40^

The reaction was performed according to the general procedure using phenol (**5f**) (0.0151 g, 0.160 mmol), *N*-(trifluoromethylthio)saccharin (**4**) (0.0500 g, 0.177 mmol), iron(III) chloride (0.000650 g, 0.00400 mmol, 2.5 mol %), and diphenyl selenide (0.000700 mL, 0.00400 mmol, 2.5 mol %). The reaction mixture was stirred at room temperature for 3 h. Purification by flash column chromatography (dichloromethane) gave 4-(trifluoromethylthio)phenol (**6f**) (0.0245 g, 79%) as a white solid. Mp 53–54 °C (lit.^40^ 53–54 °C); ^1^H NMR (400 MHz, CDCl_3_) δ 7.56–7.52 (m, 2H), 6.89–6.85 (m, 2H), 5.10 (br s, 1H); ^13^C{^1^H} NMR (101 MHz, CDCl_3_) δ 158.2 (C), 138.7 (2 × CH), 129.7 (C, q, ^1^*J*_CF_ = 308.1 Hz), 116.7 (2 × CH), 115.4 (C, q, ^3^*J*_CF_ = 2.2 Hz); MS (ESI) *m*/*z* 193 ([M–H]^−^, 100).

### 3-Chloro-4-(trifluoromethylthio)phenol (**6g**)^12b^

The reaction was performed according to the general procedure using 3-chlorophenol (**5g**) (0.0206 g, 0.160 mmol), *N*-(trifluoromethylthio)saccharin (**4**) (0.0500 g, 0.177 mmol), iron(III) chloride (0.00130 g, 0.00800 mmol, 5.0 mol %), and diphenyl selenide (0.00139 mL, 0.00800 mmol, 5.0 mol %). The reaction mixture was stirred at room temperature for 3 h. Purification by flash column chromatography (10% ethyl acetate in hexane) gave 3-chloro-4-(trifluoromethylthio)phenol (**6g**) (0.0278 g, 76%) as a colorless oil. Spectroscopic data were consistent with the literature.^12b^^1^H NMR (400 MHz, CDCl_3_) δ 7.63 (d, *J* = 8.5 Hz, 1H), 7.05 (d, *J* = 2.7 Hz, 1H), 6.79 (dd, *J* = 8.5, 2.7 Hz, 1H), 5.21 (br s, 1H); ^13^C{^1^H} NMR (101 MHz, CDCl_3_) δ 158.9 (C), 142.2 (C), 140.7 (CH), 129.4 (C, q, ^1^*J*_CF_ = 309.6 Hz), 117.9 (CH), 115.3 (CH), 114.8 (C, q, ^3^*J*_CF_ = 2.3 Hz); MS (ESI) *m*/*z* 227 ([M–H]^−^, 100).

### 1-(Trifluoromethylthio)-2-hydroxynaphthalene (**6h**)^12b^

The reaction was performed according to the general procedure using 2-hydroxynaphthalene (**5h**) (0.0231 g, 0.160 mmol), *N*-(trifluoromethylthio)saccharin (**4**) (0.0500 g, 0.177 mmol), iron(III) chloride (0.000650 g, 0.00400 mmol, 2.5 mol %), and diphenyl selenide (0.000700 mL, 0.00400 mmol, 2.5 mol %). The reaction mixture was stirred at room temperature for 0.5 h. Purification by flash column chromatography (10% ethyl acetate in hexane) gave 1-(trifluoromethylthio)-2-hydroxynaphthalene (**6h**) (0.0356 g, 91%) as a white solid. Mp 89–90 °C (lit.^12b^ 89–91 °C); ^1^H NMR (400 MHz, CDCl_3_) δ 8.34 (br d, *J* = 8.2 Hz, 1H), 7.95 (d, *J* = 8.9 Hz, 1H), 7.81 (br d, *J* = 8.2 Hz, 1H), 7.63 (ddd, *J* = 8.2, 6.9, 1.3 Hz, 1H), 7.43 (ddd, *J* = 8.2, 6.9, 1.1 Hz, 1H), 7.30 (d, *J* = 8.9 Hz, 1H), 6.92 (s, 1H); ^13^C{^1^H} NMR (101 MHz, CDCl_3_) δ 158.5 (C), 136.0 (C), 135.0 (CH), 129.6 (C), 129.0 (C, q, ^1^*J*_CF_ = 312.8 Hz), 128.7 (CH), 128.5 (CH), 124.5 (CH), 124.4 (CH), 117.2 (CH), 101.0 (C); MS (ESI) *m*/*z* 243 ([M–H]^−^, 100).

### 2-Hydroxy-3-(trifluoromethylthio)-6-methoxybenzaldehyde (**6i**)

The reaction was performed according to the general procedure using 6-methoxysalicyaldehyde (**5i**) (0.0243 g, 0.160 mmol), *N*-(trifluoromethylthio)saccharin (0.0500 g, 0.177 mmol), iron(III) chloride (0.00130 g, 0.00800 mmol, 5.0 mol %), and diphenyl selenide (0.00139 mL, 0.00800 mmol, 5.0 mol %). The reaction mixture was stirred at room temperature for 4 h. Purification by flash column chromatography (10% diethyl ether in hexane) gave 2-hydroxy-3-(trifluoromethylthio)-6-methoxybenzaldehyde (**6i**) (0.0251 g, 62%) as a white solid. Mp 118–120 °C; IR (neat) 2896, 1647, 1599, 1391, 1233, 1082, 802 cm^–1^; ^1^H NMR (400 MHz, CDCl_3_) δ 12.82 (s, 1H), 10.33 (s, 1H), 7.78 (d, *J* = 8.8 Hz, 1H), 6.49 (d, *J* = 8.8 Hz, 1H), 3.96 (s, 3H); ^13^C{^1^H} NMR (101 MHz, CDCl_3_) δ 194.5 (CH), 165.9 (C), 165.7 (C), 148.4 (CH), 129.8 (C, q, ^1^*J*_CF_ = 309.7 Hz), 111.5 (C), 103.8 (C, q, ^3^*J_CF_* = 2.0 Hz), 102.8 (CH), 56.8 (CH_3_); ^19^F NMR (376 MHz, CDCl_3_) δ −43.2 (s, 3F); MS (ESI) *m*/*z* 253 (M + H^+^, 100); HRMS (ESI) *m*/*z*: [M + H]^+^ calcd for C_9_H_8_F_3_O_3_S 253.0141; found 253.0142.

### 3,4-Methylenedioxy-6-(trifluoromethylthio)phenol (**6j**)^40^

The reaction was performed according to the general procedure using sesamol (**5j**) (0.0221 g, 0.160 mmol), *N*-(trifluoromethylthio)saccharin (**4**) (0.0500 g, 0.177 mmol), iron(III) chloride (0.00130 g, 0.00800 mmol, 5.0 mol %), and diphenyl selenide (0.00139 mL, 0.00800 mmol, 5.0 mol %). The reaction mixture was stirred at room temperature for 4 h and then 40 °C for 16 h. Purification by flash column chromatography (10% ethyl acetate in hexane) gave 3,4-methylenedioxy-6-(trifluoromethylthio)phenol (**6j**) (0.0241 g, 63%) as a white solid. Mp 80–81 °C (lit.^40^ 82–83 °C); ^1^H NMR (400 MHz, CDCl_3_) δ 6.94 (s, 1H), 6.59 (s, 1H), 6.18 (s, 1H), 5.98 (s, 2H); ^13^C{^1^H} NMR (101 MHz, CDCl_3_) δ 155.1 (C), 152.9 (C), 142.1 (C), 128.8 (C, q, ^1^*J*_*CF*_ = 311.6 Hz), 115.1 (CH), 102.2 (CH_2_), 97.9 (CH), 97.7 (C); MS (ESI) *m*/*z* 237 ([M–H]^−^, 100).

### *N*-(Trifluoromethylthio)aniline (**6k**)^41^

The reaction was performed according to the general procedure using aniline (**5k**) (0.0146 mL, 0.160 mmol), *N*-(trifluoromethylthio)saccharin (**4**) (0.0500 g, 0.177 mmol), iron(III) chloride (0.000650 g, 0.00400 mmol, 2.5 mol %), and diphenyl selenide (0.000700 mL, 0.00400 mmol, 2.5 mol %). The reaction mixture was stirred at room temperature for 0.75 h. Purification by flash column chromatography (pentane–5% diethyl ether in pentane) gave *N*-(trifluoromethylthio)aniline (**6k**) (0.0246 g, 80%) as a colorless oil. Spectroscopic data were consistent with the literature.^41^^1^H NMR (400 MHz, CDCl_3_) δ 7.31–7.24 (m, 2H), 7.11–7.05 (m, 1H), 7.00–6.94 (m, 1H), 5.08 (br s, 1H); ^13^C{^1^H} NMR (101 MHz, CDCl_3_) δ 145.2 (C), 129.5 (C, q, ^1^*J*_CF_ = 317.4 Hz), 129.5 (2 × CH), 122.1 (CH), 115.3 (2 × CH); MS (ESI) *m*/*z* 193 (M^+^, 100).

### *N*-(Trifluoromethylthio)-3-methoxy-4-methylaniline (**6l**)

The reaction was performed according to the general procedure using 3-methoxy-4-methylaniline (**5l**) (0.0219 g, 0.160 mmol), *N*-(trifluoromethylthio)saccharin (**4**) (0.0500 g, 0.177 mmol), iron(III) chloride (0.000650 g, 0.00400 mmol, 2.5 mol %), and diphenyl selenide (0.000700 mL, 0.00400 mmol, 2.5 mol %). The reaction mixture was stirred at room temperature for 0.5 h. Purification by flash column chromatography (5% ethyl acetate in hexane) gave *N*-(trifluoromethylthio)-3-methoxy-4-methylaniline (**6l**) (0.0315 g, 83%) as an orange oil. IR (neat) 3360, 2980, 1614, 1509, 1279, 1108, 1036, 962 cm^–1^; ^1^H NMR (400 MHz, CDCl_3_) δ 7.00 (d, *J* = 8.0 Hz, 1H), 6.64 (d, *J* = 2.4 Hz, 1H), 6.55 (dd, *J* = 8.0, 2.4 Hz, 1H), 5.02 (br s, 1H), 3.83 (s, 3H), 2.15 (s, 3H); ^13^C{^1^H} NMR (101 MHz, CDCl_3_) δ 158.6 (C), 144.4 (C), 131.0 (CH), 129.6 (C, q, ^1^*J*_CF_ = 317.7 Hz), 120.2 (C), 106.9 (CH), 98.2 (CH), 55.4 (CH_3_), 15.6 (CH_3_); ^19^F NMR (376 MHz, CDCl_3_) δ −52.9 (s, 3F); MS (ESI) *m*/*z* 238 (M + H^+^, 100); HRMS (ESI) *m*/*z*: [M + H]^+^ calcd for C_9_H_11_F_3_NOS 238.0508; found 238.0510.

### *N*-(Trifluoromethylthio)-2-cyanoaniline (**6m**)

The reaction was performed according to the general procedure using 2-cyanoaniline (**5m**) (0.0189 g, 0.160 mmol), *N*-(trifluoromethylthio)saccharin (**4**) (0.0500 g, 0.177 mmol), iron(III) chloride (0.000650 g, 0.00400 mmol, 2.5 mol %), and diphenyl selenide (0.000700 mL, 0.00400 mmol, 2.5 mol %). The reaction mixture was stirred at room temperature for 22 h. Purification by flash column chromatography (5% ethyl acetate in hexane) gave *N*-(trifluoromethylthio)-2-cyanoaniline (**6m**) (0.0245 g, 70%) as a white solid. Mp 88–90 °C; IR (neat) 3286, 2821, 2224, 1604, 1575, 1489, 1286, 1116, 921, 755 cm^–1^; ^1^H NMR (400 MHz, CDCl_3_) δ 7.60–7.48 (m, 3H), 7.06–6.99 (m, 1H), 5.95 (br s, 1H); ^13^C{^1^H} NMR (101 MHz, CDCl_3_) δ 148.1 (C), 134.6 (CH), 132.8 (CH), 129.1 (C, q, ^1^*J*_CF_ = 316.8 Hz), 122.0 (CH), 116.5 (C), 114.7 (CH), 99.8 (C); ^19^F NMR (376 MHz, CDCl_3_) δ −52.3 (s, 3F); MS (ESI) *m*/*z* 219 (M + H^+^, 100); HRMS (ESI) *m*/*z*: [M + H]^+^ calcd for C_8_H_6_F_3_N_2_S 219.0198; found 219.0200.

### Benzyl [4-(Trifluoromethylthio)benzene]carbamate (**6n**)

The reaction was performed according to the general procedure using benzyl benzenecarbamate (**5n**) (0.0364 g, 0.160 mmol), *N*-(trifluoromethylthio)saccharin (**4**) (0.0500 g, 0.177 mmol), iron(III) chloride (0.00260 g, 0.0160 mmol, 10 mol %), and diphenyl selenide (0.00279 mL, 0.0160 mmol, 10 mol %). The reaction mixture was stirred at room temperature for 7 h. Purification by flash column chromatography (40% dichloromethane in hexane) gave benzyl [4-(trifluoromethylthio)benzene]carbamate (**6n**) (0.0320 g, 61%) as a white solid. Mp 104–106 °C; IR (neat) 3262, 3070, 1699, 1589, 1526, 1239, 1115, 830, 739 cm^–1^; ^1^H NMR (400 MHz, CDCl_3_) δ 7.62–7.56 (m, 2H), 7.48–7.44 (m, 2H), 7.43–7.33 (m, 5H), 6.80 (br s, 1H), 5.22 (s, 2H); ^13^C{^1^H} NMR (101 MHz, CDCl_3_) δ 153.0 (C), 140.6 (C), 137.8 (2 × CH), 135.8 (C), 129.7 (C, q, ^1^*J*_CF_ = 308.1 Hz), 128.9 (2 × CH), 128.7 (CH), 128.6 (2 × CH), 119.1 (2 × CH), 118.1 (C, q, ^3^*J*_CF_ = 2.3 Hz), 67.6 (CH_2_); ^19^F NMR (376 MHz, CDCl_3_) δ −43.4 (s, 3F); MS (APCI) *m*/*z* 328 (M + H^+^, 100); HRMS (APCI) *m*/*z*: [M + H]^+^ calcd for C_15_H_13_F_3_NO_2_S 328.0614; found 328.0615.

### *N*-(Benzyloxycarbonyl)-3′-(trifluoromethylthio)-L-tyrosine Methyl Ester (**6o**)

The reaction was performed according to the general procedure using *N*-(benzyloxycarbonyl)-L-tyrosine methyl ester (**5o**) (0.0527 g, 0.160 mmol), *N*-(trifluoromethylthio)saccharin (**4**) (0.0500 g, 0.177 mmol), iron(III) chloride (0.00260 g, 0.0160 mmol, 10 mol %), and diphenyl selenide (0.00279 mL, 0.0160 mmol, 10 mol %). The reaction mixture was stirred at 40 °C for 48 h. Purification by flash column chromatography (30% ethyl acetate in hexane) gave *N*-(benzyloxycarbonyl)-3′-(trifluoromethylthio)-L-tyrosine methyl ester (**6o**) (0.0482 g, 70%) as a white solid. Mp 109–111 °C; [α]_D_^17^ +69.6 (*c* 0.1, CHCl_3_); IR (neat) 3420, 3301, 2947, 1735, 1691, 1541, 1486, 1271, 1190, 1107, 733 cm^–1^; ^1^H NMR (400 MHz, CDCl_3_) δ 7.39–7.29 (m, 6H), 7.16 (dd, *J* = 8.4, 2.2 Hz, 1H), 6.96 (d, *J* = 8.4 Hz, 1H), 6.31 (s, 1H), 5.28 (br d, *J* = 7.9 Hz, 1H), 5.13 (d, *J* = 12.0 Hz, 1H), 5.08 (d, *J* = 12.0 Hz, 1H), 4.67–4.60 (m, 1H), 3.72 (s, 3H), 3.11 (dd, *J* = 14.1, 5.8 Hz, 1H), 3.03 (dd, *J* = 14.1, 5.8 Hz, 1H); ^13^C{^1^H} NMR (101 MHz, CDCl_3_) δ 171.7 (C), 157.4 (C), 155.7 (C), 138.8 (CH), 136.3 (C), 135.4 (CH), 129.1 (C), 128.8 (C, q, ^1^*J*_CF_ = 310.6 Hz), 128.7 (2 × CH), 128.4 (CH), 128.3 (2 × CH), 116.6 (CH), 108.5 (C), 67.2 (CH_2_), 54.9 (CH), 52.6 (CH_3_), 37.2 (CH_2_); ^19^F NMR (376 MHz, CDCl_3_) δ −42.8 (s, 3F); MS (ESI) *m*/*z* 430 (M + H^+^, 100); HRMS (ESI) *m*/*z*: [M + H]^+^ calcd for C_19_H_19_F_3_NO_5_S 430.0931; found 430.0934.

### 5-[(3′,5′-Dimethyl-4′-(trifluoromethylthio)phenoxy)methyl]-1,3-oxazolidin-2-one (**6p**)

The reaction was performed according to the general procedure using metaxalone (**5p**) (0.0354 g, 0.160 mmol), *N*-(trifluoromethylthio)saccharin (**4**) (0.0500 g, 0.177 mmol), iron(III) chloride (0.00260 g, 0.0160 mmol, 10 mol %), and diphenyl selenide (0.00279 mL, 0.0160 mmol, 10 mol %). The reaction mixture was stirred at 40 °C for 48 h. Purification by flash column chromatography (30% ethyl acetate in hexane) gave 5-[(3′,5′-dimethyl-4’-(trifluoromethylthio)phenoxy)methyl]-1,3-oxazolidin-2-one (**6p**) (0.0353 g, 69%) as a white solid. Mp 95–97 °C; IR (neat) 3466, 2981, 1748, 1591, 1311, 1231, 1153, 1095, 964 cm^–1^; ^1^H NMR (400 MHz, CDCl_3_) δ 6.73 (s, 2H), 6.14 (br s, 1H), 5.00–4.92 (m, 1H), 4.15 (d, *J* = 4.8 Hz, 2H), 3.78 (t, *J* = 8.8 Hz, 1H), 3.59 (dd, *J* = 8.8, 6.1 Hz, 1H), 2.53 (s, 6H); ^13^C{^1^H} NMR (101 MHz, CDCl_3_) δ 159.9 (C), 159.7 (C), 147.6 (2 × C), 130.1 (C, q, ^1^*J*_CF_ = 309.6 Hz), 115.7 (C, q, ^3^*J*_CF_ = 1.8 Hz), 114.8 (2 × CH), 74.1 (CH), 67.9 (CH_2_), 42.8 (CH_2_), 22.6 (2 × CH_3_); ^19^F NMR (376 MHz, CDCl_3_) δ −42.4 (s, 3F); MS (APCI) *m*/*z* 322 (M + H^+^, 100); HRMS (APCI) *m*/*z*: [M + H]^+^ calcd for C_13_H_15_F_3_NO_3_S 322.0719; found 322.0721.

### 2-(Trifluoromethylthio)-β-estradiol (**6q**)

The reaction was performed according to the general procedure using β-estradiol (**5q**) (0.0436 g, 0.160 mmol), *N*-(trifluoromethylthio)saccharin (**4**) (0.0500 g, 0.177 mmol), iron(III) chloride (0.00130 g, 0.00800 mmol, 5.0 mol %), and diphenyl selenide (0.00139 mL, 0.00800 mmol, 5.0 mol %). The reaction mixture was stirred at 40 °C for 22 h. Purification by flash column chromatography (40% diethyl ether in hexane) gave 2-(trifluoromethylthio)-β-estradiol (0.0328 g, 55%) (**6q**) as a white solid. Mp 112–115 °C; [α]_D_^17^ +113.7 (*c* 0.1, CHCl_3_); IR (neat) 3328, 2928, 1604, 1485, 1103, 905, 733 cm^–1^; ^1^H NMR (400 MHz, CDCl_3_) δ 7.44 (s, 1H), 6.79 (s, 1H), 6.06 (s, 1H), 3.77–3.70 (m, 1H), 2.89–2.83 (m, 2H), 2.35–2.26 (m, 1H), 2.21–2.08 (m, 2H), 2.00–1.93 (m, 1H), 1.92–1.85 (m, 1H), 1.75–1.66 (m, 1H), 1.55–1.15 (m, 7H), 0.79 (s, 3H); ^13^C{^1^H} NMR (101 MHz, CDCl_3_) δ 155.8 (C), 144.4 (C), 135.2 (CH), 134.4 (C), 129.0 (C, q, ^1^*J*_CF_ = 310.5 Hz), 116.0 (CH), 105.3 (C, q, ^3^*J*_CF_ = 2.0 Hz), 81.2 (CH), 50.2 (CH), 43.7 (CH), 43.4 (C), 38.6 (CH), 36.7 (CH_2_), 30.7 (CH_2_), 29.8 (CH_2_), 27.0 (CH_2_), 26.4 (CH_2_), 23.3 (CH_2_), 11.2 (CH_3_); ^19^F NMR (376 MHz, CDCl_3_) δ −43.2 (s, 3F); MS (ESI) *m*/*z* 355 (MH^+^–H_2_O, 100); HRMS (ESI) *m*/*z*: [M + H – H_2_O]^+^ calcd for C_19_H_22_F_3_OS 355.1338; found 355.1340.

### 3-(Trifluoromethylthio)indole (**9a**)^12b^

The reaction was performed according to the general procedure using indole (**7a**) (0.0187 g, 0.160 mmol), *N*-(trifluoromethylthio)saccharin (**4**) (0.0500 g, 0.177 mmol), iron(III) chloride (0.000650 g, 0.00400 mmol, 2.5 mol %), and diphenyl selenide (0.000700 mL, 0.00400 mmol, 2.5 mol %). The reaction mixture was stirred at room temperature for 2 h. Purification by flash column chromatography (30% diethyl ether in hexane) gave 3-(trifluoromethylthio)indole (**9a**) (0.0335 g, 96%) as an orange oil. Spectroscopic data were consistent with the literature.^12b^^1^H NMR (400 MHz, CDCl_3_) δ 8.50 (br s, 1H), 7.84–7.79 (m, 1H), 7.55 (d, *J* = 2.6 Hz, 1H), 7.46–7.40 (m, 1H), 7.34–7.26 (m, 2H); ^13^C{^1^H} NMR (101 MHz, CDCl_3_) δ 136.2 (C), 132.9 (CH), 129.6 (C), 129.6 (C, q, ^1^*J*_CF_ = 309.9 Hz), 123.6 (CH), 121.8 (CH), 119.5 (CH), 111.8 (CH), 95.8 (C, q, ^3^*J*_CF_ = 2.4 Hz); MS (ESI) *m*/*z* 216 ([M–H]^−^, 100).

### 3-(Trifluoromethylthio)-4-fluoroindole (**9b**)

The reaction was performed according to the general procedure using 4-fluoroindole (**7b**) (0.0216 g, 0.160 mmol), *N*-(trifluoromethylthio)saccharin (**4**) (0.0500 g, 0.177 mmol), iron(III) chloride (0.00130 g, 0.00800 mmol, 5.0 mol %) and diphenyl selenide (0.00139 mL, 0.00800 mmol, 5.0 mol %). The reaction mixture was stirred at room temperature for 3 h. Purification by flash column chromatography (10% ethyl acetate in hexane) gave 3-(trifluoromethylthio)-4-fluoroindole (**9b**) (0.0344 g, 91%) as a white solid. Mp 91–93 °C; IR (neat) 3453, 1577, 1509, 1318, 1235, 1092, 1007, 730 cm^–1^; ^1^H NMR (400 MHz, CDCl_3_) δ 8.62 (br s, 1H), 7.50 (d, *J* = 2.8 Hz, 1H), 7.24–7.16 (m, 2H), 6.96–6.87 (m, 1H); ^13^C{^1^H} NMR (101 MHz, CDCl_3_) δ 156.8 (C, d, ^1^*J*_CF_ = 250.7 Hz), 138.9 (C, d, ^3^*J*_CF_ = 9.5 Hz), 133.7 (CH), 129.4 (C, q, ^1^*J*_CF_ = 309.4 Hz), 124.3 (CH, d, ^3^*J*_CF_ = 7.8 Hz), 118.2 (C, d,^2^*J*_CF_ = 19.0 Hz), 108.0 (CH, d, ^4^*J*_CF_ = 4.2 Hz), 107.4 (CH, d, ^2^*J*_CF_ = 19.0 Hz), 93.6 (C, q, ^3^*J*_CF_ = 3.0 Hz); ^19^F NMR (376 MHz, CDCl_3_) δ −45.5 (d, ^6^*J*_FF_ = 4.6 Hz, 3F), −124.2 (m, F); MS (APCI) *m*/*z* 236 (M + H^+^, 100); HRMS (APCI) *m*/*z*: [M + H]^+^ calcd for C_9_H_6_F_4_NS 236.0152; found 236.0149.

### 3-(Trifluoromethylthio)-5-nitroindole (**9c**)^37^

The reaction was performed according to the general procedure using 5-nitroindole (**7c**) (0.0259 g, 0.160 mmol), *N*-(trifluoromethylthio)saccharin (**4**) (0.0500 g, 0.177 mmol), iron(III) chloride (0.00130 g, 0.00800 mmol, 5.0 mol %), and diphenyl selenide (0.00139 mL, 0.00800 mmol, 5.0 mol %). The reaction mixture was stirred at room temperature for 1 h. Purification by flash column chromatography (25% ethyl acetate in hexane) gave 3-(trifluoromethylthio)-4-fluoroindole (**9c**) (0.0395 g, 94%) as a yellow solid. Mp 177–178 °C (lit.^37^ 170–172 °C); ^1^H NMR (400 MHz, CDCl_3_) δ 8.99 (br s, 1H), 8.76 (d, *J* = 2.2 Hz, 1H), 8.22 (dd, *J* = 9.0, 2.5 Hz, 1H), 7.75 (d, *J* = 2.5 Hz, 1H), 7.54 (d, *J* = 9.0 Hz, 1H); ^13^C{^1^H} NMR (101 MHz, CDCl_3_) δ 143.7 (C), 139.1 (C), 135.9 (CH), 129.4 (C), 129.2 (C, q, ^1^*J*_CF_ = 309.9 Hz), 119.3 (CH), 116.8 (CH), 112.3 (CH), 99.0 (C, q, ^3^*J*_CF_ = 2.4 Hz); MS (APCI) *m*/*z* 263 (M + H^+^, 100).

### 3-(Trifluoromethylthio)indole-2-carboxylic Acid Ethyl Ester (**9d**)

The reaction was performed according to the general procedure using indole-2-carboxylic acid ethyl ester (**7d**) (0.0303 g, 0.160 mmol), *N*-(trifluoromethylthio)saccharin (**4**) (0.0500 g, 0.177 mmol), iron(III) chloride (0.00130 g, 0.00800 mmol, 5.0 mol %), and diphenyl selenide (0.00139 mL, 0.00800 mmol, 5.0 mol %). The reaction mixture was stirred at room temperature for 15 min. Purification by flash column chromatography (5% ethyl acetate in hexane) gave 3-(trifluoromethylthio)indole-2-carboxylic acid ethyl ester (**9d**) (0.0443 g, 96%) as a white solid. Mp 136–137 °C; IR (neat) 3289, 2992, 1676, 1510, 1333, 1260, 1105, 1014, 740 cm^–1^; ^1^H NMR (400 MHz, CDCl_3_) δ 9.47 (br s, 1H), 7.90 (br d, *J* = 8.1 Hz, 1H), 7.46 (br d, *J* = 8.2 Hz, 1H), 7.42 (ddd, *J* = 8.2, 6.8, 1.2 Hz, 1H), 7.32 (ddd, *J* = 8.1, 6.8, 1.2 Hz, 1H), 4.50 (q, *J* = 7.1 Hz, 2H), 1.47 (t, *J* = 7.1 Hz, 3H); ^13^C{^1^H} NMR (101 MHz, CDCl_3_) δ 160.9 (C), 135.2 (C), 131.4 (C), 131.3 (C), 129.5 (C, q, ^1^*J*_CF_ = 310.3 Hz), 126.5 (CH), 122.7 (CH), 121.3 (CH), 112.3 (CH), 100.4 (C, q, ^3^*J*_CF_ = 2.5 Hz), 62.1 (CH_2_), 14.3 (CH_3_); ^19^F NMR (376 MHz, CDCl_3_) δ −43.0 (s, 3F); MS (APCI) *m*/*z* 290 (M + H^+^, 100); HRMS (APCI) *m*/*z*: [M + H]^+^ calcd for C_12_H_11_F_3_NO_2_S 290.0457; found 290.0458.

### 3-(Trifluoromethylthio)carbazole (**10a**)^36^

The reaction was performed according to the general procedure using carbazole (**8a**) (0.0268 g, 0.160 mmol), *N*-(trifluoromethylthio)saccharin (**4**) (0.0500 g, 0.177 mmol), iron(III) chloride (0.00130 g, 0.00800 mmol, 5.0 mol %), and diphenyl selenide (0.00139 mL, 0.00800 mmol, 5.0 mol %). The reaction mixture was stirred at room temperature for 7 h. Purification by flash column chromatography (7.5% ethyl acetate in hexane) gave 3-(trifluoromethylthio)carbazole (**10a**) (0.0351 g, 82%) as a white solid. Mp 143–145 °C (lit.^36^ 146–147 °C); ^1^H NMR (400 MHz, CDCl_3_) δ 8.38 (d, *J* = 1.7 Hz, 1H), 8.19 (br s, 1H), 8.10 (br d, *J* = 8.0 Hz, 1H), 7.68 (dd, *J* = 8.4, 1.7 Hz, 1H), 7.51–7.42 (m, 3H), 7.30 (ddd, *J* = 8.0, 6.6, 1.6 Hz, 1H); ^13^C{^1^H} NMR (101 MHz, CDCl_3_) δ 140.8 (C), 139.9 (C), 134.1 (CH), 130.0 (C, q, ^1^*J*_CF_ = 308.2 Hz), 129.7 (CH), 127.0 (CH), 124.5 (C), 122.7 (C), 120.8 (CH), 120.5 (CH), 113.7 (C, q, ^3^*J*_CF_ = 2.2 Hz), 111.5 (CH), 111.0 (CH); MS (APCI) *m*/*z* 268 (M + H^+^, 100).

### 3-Iodo-6-(trifluoromethylthio)carbazole (**10b**)

The reaction was performed according to the general procedure using 3-iodocarbazole (**8b**) (0.0469 g, 0.160 mmol), *N*-(trifluoromethylthio)saccharin (**4**) (0.0500 g, 0.177 mmol), iron(III) chloride (0.00130 g, 0.00800 mmol, 5.0 mol %), and diphenyl selenide (0.00139 mL, 0.00800 mmol, 5.0 mol %). The reaction mixture was stirred at room temperature for 15 min. Purification by flash column chromatography (10–15% ethyl acetate in hexane) gave 3-iodo-6-(trifluoromethylthio)carbazole (**10b**) (0.0590 g, 94%) as a white solid. Mp 134–136 °C; IR (neat) 3483, 2918, 1597, 1468, 1287, 1098, 868, 806 cm^–1^; ^1^H NMR (400 MHz, CDCl_3_) δ 8.40 (d, *J* = 1.7 Hz, 1H), 8.31 (br d, *J* = 1.8 Hz, 1H), 8.22 (br s, 1H), 7.72 (dd, *J* = 8.5, 1.7 Hz, 1H), 7.70 (dd, *J* = 8.5, 1.8 Hz, 1H), 7.44 (d, *J* = 8.5 Hz, 1H), 7.24 (d, *J* = 8.5 Hz, 1H); ^13^C{^1^H} NMR (101 MHz, CDCl_3_) δ 140.7 (C), 139.0 (C), 135.3 (CH), 134.7 (CH), 129.9 (C, q, ^1^*J*_CF_ = 308.4 Hz), 129.9 (CH), 129.7 (CH), 125.2 (C), 123.2 (C), 114.4 (C, q, ^3^*J*_CF_ = 2.3 Hz), 113.0 (CH), 111.7 (CH), 83.1 (C); ^19^F NMR (376 MHz, CDCl_3_) δ −43.9 (s, 3F); MS (APCI) *m*/*z* 393 (M^+^, 100); HRMS (APCI) *m*/*z*: [M]^+^ calcd for C_13_H_7_F_3_INS 392.9290; found 392.9290.

### 3-Iodo-6-(trifluoromethylthio)carbazole (**10b**): Large-Scale Reaction

The reaction was performed according to the general procedure using 3-iodocarbazole (**8b**) (0.293 g, 1.00 mmol), *N*-(trifluoromethylthio)saccharin (**4**) (0.312 g, 1.10 mmol), iron(III) chloride (0.00811 g, 0.0500 mmol, 5.0 mol %), and diphenyl selenide (0.00817 mL, 0.0500 mmol, 5.0 mol %). The reaction mixture was stirred at room temperature for 15 min. Purification by flash column chromatography (10–15% ethyl acetate in hexane) gave 3-iodo-6-(trifluoromethylthio)carbazole (**10b**) (0.366 g, 93%) as a white solid. Spectroscopic data are as described above.

### 3-(Trifluoromethylthio)-4-hydroxycarbazole (**10c**) and 1-(Trifluoromethylthio)-4-hydroxycarbazole (**10d**)

The reaction was performed according to the general procedure using 4-hydroxycarbazole (**8c**) (0.0293 g, 0.160 mmol), *N*-(trifluoromethylthio)saccharin (**4**) (0.0500 g, 0.177 mmol), iron(III) chloride (0.00065 g, 0.00400 mmol, 2.5 mol %), and diphenyl selenide (0.000700 mL, 0.00400 mmol, 2.5 mol %). The reaction mixture was stirred at room temperature for 1 h. Purification by flash column chromatography (20% dichloromethane in hexane) gave 3-(trifluoromethylthio)-4-hydroxycarbazole (**10c**) (0.0230 g, 51%) as a white solid. Mp 184–186 °C; IR (neat) 3443, 3386, 2919, 1602, 1443, 1247, 1092, 755 cm^–1^; ^1^H NMR (400 MHz, CDCl_3_) δ 8.34 (d, *J* = 8.0 Hz, 1H), 8.23 (br s, 1H), 7.55 (d, *J* = 8.4 Hz, 1H), 7.34–7.29 (m, 2H), 7.32 (ddd, *J* = 8.0, 5.5, 2.7 Hz, 1H), 7.04 (d, *J* = 8.4 Hz, 1H), 7.01 (br s, 1H); ^13^C{^1^H} NMR (101 MHz, CDCl_3_) δ 154.9 (C), 143.8 (C), 139.0 (C), 135.0 (CH), 129.0 (C, q, ^1^*J*_CF_ = 311.1 Hz), 126.0 (CH), 123.3 (CH), 122.3 (C), 120.9 (CH), 111.6 (C), 110.5 (CH), 104.6 (CH), 96.8 (C); ^19^F NMR (376 MHz, CDCl_3_) δ −44.1 (s, 3F); MS (ESI) *m*/*z* 284 (M + H^+^, 100); HRMS (ESI) *m*/*z*: [M + H]^+^ calcd for C_13_H_9_F_3_NOS 284.0351; found 284.0352. Further elution (50% dichloromethane in hexane) gave 1-(trifluoromethylthio)-4-hydroxycarbazole (**10d**) (0.0132 g, 29%) as a white solid. Mp 153–155 °C; IR (neat) 3473, 3352, 2919, 1584, 1442, 1295, 1100, 801, 745 cm^–1^; ^1^H NMR (400 MHz, CDCl_3_) δ 8.58 (br s, 1H), 8.30–8.25 (m, 1H), 7.53 (d, *J* = 8.2 Hz, 1H), 7.51 (ddd, *J* = 8.2, 1.3, 0.8 Hz, 1H), 7.46 (ddd, *J* = 8.2, 6.9, 1.2 Hz, 1H), 7.31 (ddd, *J* = 7.8, 6.9, 1.3 Hz, 1H), 6.63 (d, *J* = 8.2 Hz, 1H), 5.91 (br s, 1H); ^13^C{^1^H} NMR (101 MHz, CDCl_3_) δ 155.2 (C), 144.8 (C), 138.5 (C), 136.6 (CH), 129.7 (C, q, ^1^*J*_CF_ = 310.5 Hz), 126.1 (CH), 123.1 (CH), 122.6 (C), 120.7 (CH), 112.3 (C), 110.7 (CH), 106.9 (CH), 95.6 (C); ^19^F NMR (376 MHz, CDCl_3_) δ −43.2 (s, 3F); MS (ESI) *m*/*z* 284 (M + H^+^, 100); HRMS (ESI) *m*/*z*: [M + H]^+^ calcd for C_13_H_9_F_3_NOS 284.0351; found 284.0353.

### 1-[(2′,6′-Dichloro-4′-trifluoromethyl)benzene]indole (**12**)

To a solution of indole (**7a**) (0.0879 g, 0.750 mmol) in dry *N*,*N*′-dimethylformamide (5 mL) under argon were added potassium carbonate (0.124 g, 0.900 mmol) and 1,3-dichloro-2-fluoro-5-(trifluoromethyl)benzene (**11**) (0.135 mL, 0.900 mmol), and the reaction mixture was stirred at 90 °C for 18 h. After cooling to room temperature, the reaction mixture was diluted with ethyl acetate (20 mL) and washed with water (3 × 10 mL) and brine (20 mL). The organic phase was dried (MgSO_4_), filtered, and concentrated *in vacuo*. Purification by flash column chromatography (hexane–1% ethyl acetate in hexane) gave 1-[(2’,6’-dichloro-4’-trifluoromethyl)benzene]indole (**12**) as a yellow oil (0.220 g, 89%). IR (neat) 3079, 1491, 1453, 1302, 1133, 880, 815, 737 cm^–1^; ^1^H NMR (400 MHz, CDCl_3_) δ 7.79 (s, 2H), 7.75–7.69 (m, 1H), 7.25–7.19 (m, 2H), 7.10 (d, *J* = 3.3 Hz, 1H), 6.96–6.90 (m, 1H), 6.79 (dd, *J* = 3.3, 0.9 Hz, 1H); ^13^C{^1^H} NMR (101 MHz, CDCl_3_) δ 138.3 (C), 136.6 (2 × C), 135.9 (C), 132.4 (C, q, ^2^*J*_CF_ = 34.4 Hz), 128.5 (C), 127.6 (CH), 126.2 (2 × CH, q, ^3^*J*_CF_ = 3.7 Hz), 123.0 (CH), 122.5 (C, q, ^1^*J*_CF_ = 310.6 Hz), 121.4 (CH), 121.0 (CH), 110.3 (CH), 104.8 (CH); ^19^F NMR (376 MHz, CDCl_3_) δ −63.0 (s, 3F); MS (APCI) *m*/*z* 330 (M + H^+^, 100); HRMS (APCI) *m*/*z*: [M + H]^+^ calcd for C_15_H_9_^35^ Cl_2_F_3_N 330.0059; found 330.0057.

### 1-[(2′,6′-Dichloro-4′-trifluoromethyl)benzene]-3-(trifluoromethylthio)indole (**13**)

The reaction was performed according to the general procedure using 1-[(2′,6′-dichloro-4′-trifluoromethyl)benzene]indole (**12**) (0.0528 g, 0.160 mmol), *N*-(trifluoromethylthio)saccharin (**4**) (0.0500 g, 0.177 mmol), iron(III) chloride (0.00130 g, 0.00800 mmol, 5.0 mol %), and diphenyl selenide (0.00139 mL, 0.00800 mmol, 5.0 mol %). The reaction mixture was stirred at room temperature for 0.5 h. Purification by flash column chromatography (hexane) gave 1-[(2′,6′-dichloro-4′-trifluoromethyl)benzene]-3-(trifluoromethylthio)indole (**13**) (0.0622 g, 90%) as a white solid. Mp 76–78 °C; IR (neat) 3115, 1515, 1366, 1305, 1099, 817, 746 cm^–1^; ^1^H NMR (400 MHz, CDCl_3_) δ 7.89 (br d, *J* = 7.6 Hz, 1H), 7.82 (s, 2H), 7.45 (s, 1H), 7.40–7.29 (m, 2H), 6.99–6.95 (m, 1H); ^13^C{^1^H} NMR (101 MHz, CDCl_3_) δ 137.0 (C), 136.4 (C), 136.4 (2 × C), 135.6 (CH), 133.3 (C, q, ^2^*J*_CF_ = 34.3 Hz), 129.8 (C), 129.4 (C, q, ^1^*J*_CF_ = 310.0 Hz), 126.3 (2 × CH, q, ^3^*J*_CF_ = 3.7 Hz), 124.4 (CH), 122.7 (CH), 122.4 (C, q, ^1^*J*_CF_ = 273.6 Hz), 120.1 (CH), 110.8 (CH), 98.5 (C, q, ^3^*J*_CF_ = 2.7 Hz); ^19^F NMR (376 MHz, CDCl_3_) δ −44.2 (s, 3F), −63.1 (s, 3F); MS (ESI) *m*/*z* 430 (M + H^+^, 100); HRMS (ESI) *m*/*z*: [M + H]^+^ calcd for C_16_H_8_^35^ Cl_2_F_6_NS 429.9653; found 429.9656.

## Data Availability

The data underlying this study are available in the published article and its online Supporting Information.

## Supporting Information Available

X-ray data for compound **6i**, ^1^H and ^13^C NMR spectra for all compounds, and ^19^F NMR spectra for all novel compounds (PDF)
