# Supplementary material for: Trifluoromethylthiolation of Arenes Using Lewis Acid and Lewis Base Dual Catalysis
Source: J Org Chem. 2023 Dec 29;89(2):1275–84. doi: 10.1021/acs.joc.3c02571 (PMC10804413; doi:10.1021/acs.joc.3c02571)

Supporting Information for:

Trifluoromethylthiolation of Arenes using Lewis Acid and Lewis Base Dual Catalysis

Lachlan J. N. Waddell, Claire Wilson and Andrew Sutherland*

School of Chemistry, The Joseph Black Building, University of Glasgow, Glasgow G12 8QQ, UK. Email: Andrew.Sutherland@glasgow.ac.uk.

Table of Contents

1. Crystal Structure and Data for **6i** S2–S3
2. ^{1}H and ^{13}C NMR Spectra for all Compounds and ^{19}F NMR Spectra for all Novel Compounds S4–S71

1. Crystal Structure and Data for 6i (CCDC 2305671)

2-Hydroxy-3-(trifluoromethylthio)-6-methoxybenzaldehyde **(6i)** was recrystallized from diethyl ether. Following crystallization, the solvent was allowed to evaporate at room temperature over 18 hours. The crystals were collected and X-ray crystallographic analysis was performed.

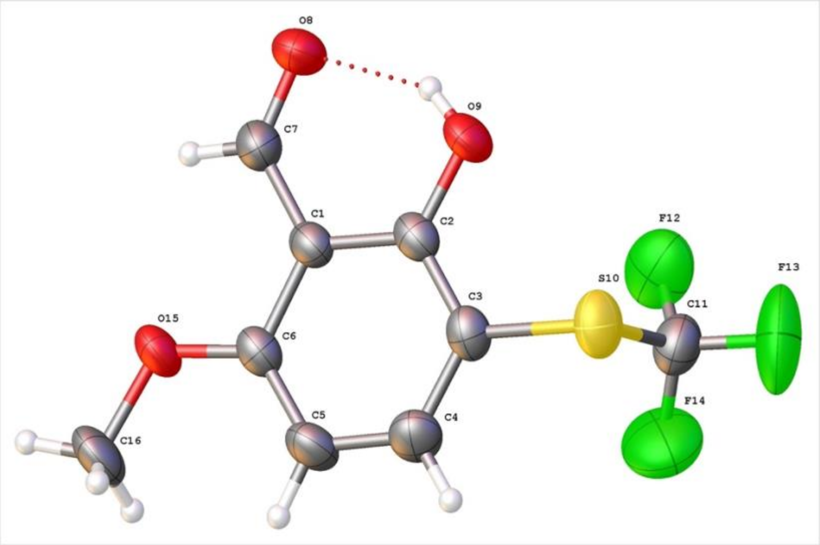

View showing the structure and atom labelling scheme for **6i**. Displacement ellipsoids are drawn at 50% probability level.

Computing details: Data collection: *APEX3* Ver. 2016.9-0 (Bruker-AXS, 2016); cell refinement: *SAINT* V8.37A (Bruker-AXS, 2016); data reduction: *APEX3* Ver. 2016.9-0 (Bruker-AXS, 2016); program(s) used to solve structure: SHELXT 2018/2;[1] program(s) used to refine structure: *SHELXL* 2018/3;[2] molecular graphics: Olex2 1.5; software used to prepare material for publication: Olex2 1.5.[3]

Crystal data

$C_9H_7F_3O_3S$	$F(000) = 512$
$M_r = 252.21$	$D_x = 1.649$ Mg m^{-3}
Monoclinic, $P2_1/n$	Mo $K\alpha$ radiation, $\lambda = 0.71073$ Å
$a = 7.9284$ (8) Å	Cell parameters from 6104 reflections
$b = 4.8567$ (5) Å	$\theta = 2.8–26.3°$
$c = 26.543$ (3) Å	$\mu = 0.35$ mm^{-1}
$\beta = 96.455$ (3)°	$T = 295$ K
$V = 1015.58$ (18) Å^3	Block, colorless
$Z = 4$	0.28 × 0.16 × 0.1 mm

Data collection

Bruker D8 VENTURE diffractometer	2065 independent reflections
Radiation source: microfocus sealed tube, INCOATEC Iμs 3.0	1825 reflections with $I > 2\sigma(I)$
Multilayer mirror optics monochromator	R_{int} = 0.036
Detector resolution: 7.4074 pixels mm^{-1}	θ_{max} = 26.4°, θ_{min} = 2.6°
ϕ and ω scans	$h = -9\rightarrow9$
Absorption correction: multi-scan *SADABS2016*/2 (Bruker,2016/2) was used for absorption correction. wR2(int) was 0.0657 before and 0.0560 after correction. The Ratio of minimum to maximum transmission is 0.8373. The λ/2 correction factor is Not present.	$k = -6\rightarrow6$
T_{min} = 0.624, T_{max} = 0.745	$l = -33\rightarrow33$
11249 measured reflections	

Refinement

Refinement on F^2	Primary atom site location: dual
Least-squares matrix: full	Hydrogen site location: inferred from neighbouring sites
$R[F^2 > 2\sigma(F^2)]$ = 0.054	H-atom parameters constrained
$wR(F^2)$ = 0.125	$w = 1/[\sigma^2(F_o^2) + (0.024P)^2 + 1.4201P]$ where $P = (F_o^2 + 2F_c^2)/3$
S = 1.18	$(\Delta/\sigma)_{max}$ < 0.001
2065 reflections	$\Delta\rangle_{max}$ = 0.28 e $Å^{-3}$
147 parameters	$\Delta\rangle_{min}$ = -0.29 e $Å^{-3}$
0 restraints	

References

1. Sheldrick, G. M. *SHELXT* – Integrated Space-Group and Crystal-Structure Determination. *Acta Cryst*. **2015**, *A71*, 3–8.

2. Sheldrick, G. M. Crystal Structure Refinement with *SHELXL*. *Acta Cryst.* **2015**, *C71*, 3–8.

3. Dolomanov, O. V.; Bourhis, L. J.; Gildea, R. J.; Howard, J. A. K.; Puschmann, H. *OLEX2*: A Complete Structure Solution, Refinement and Analysis Program. *J. Appl. Cryst*. **2009**, *42*, 339–341.

1. ^{1}H and ^{13}C NMR Spectra for All Compounds and ^{19}F NMR Spectra for all Novel Compounds

^{1}H NMR (400 MHz, $CDCl_3$)

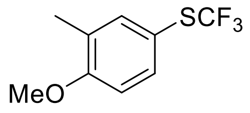

6a

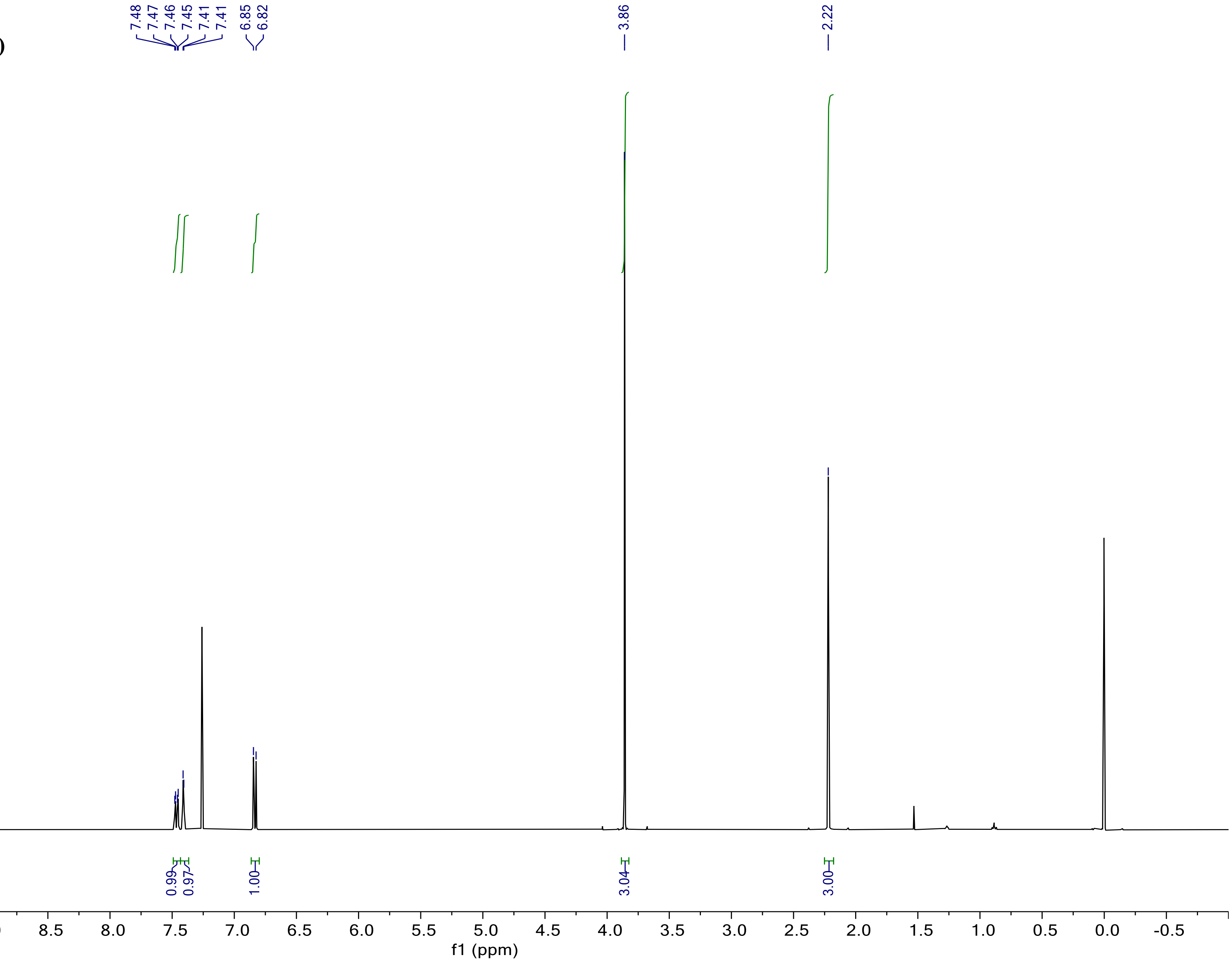

$^{13}C\{^{1}H\}$ NMR (101 MHz, $CDCl_3$)

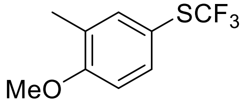

6a

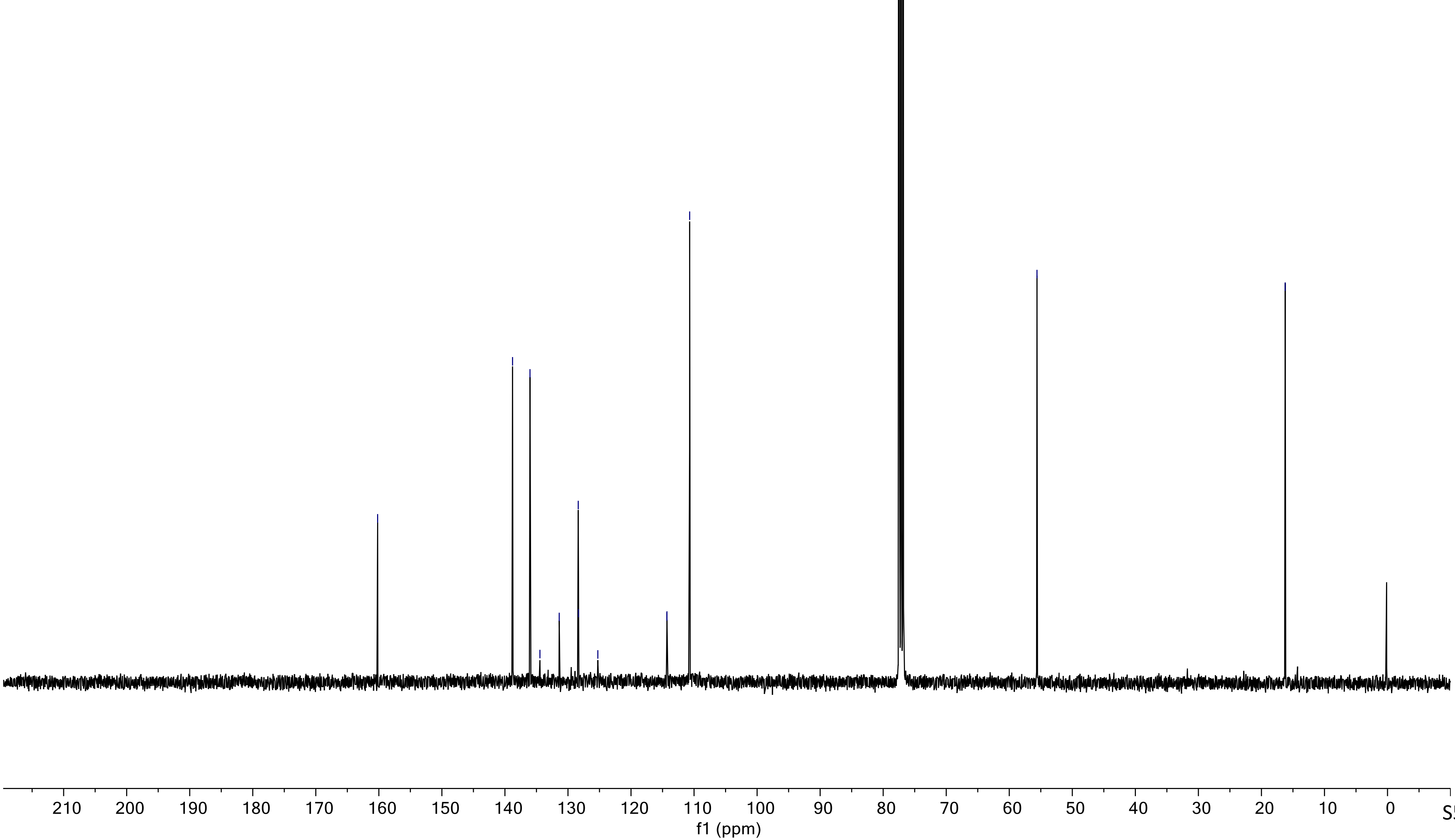

^{1}H NMR (400 MHz, $CDCl_3$)

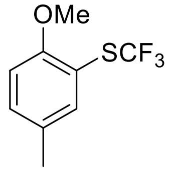

6b

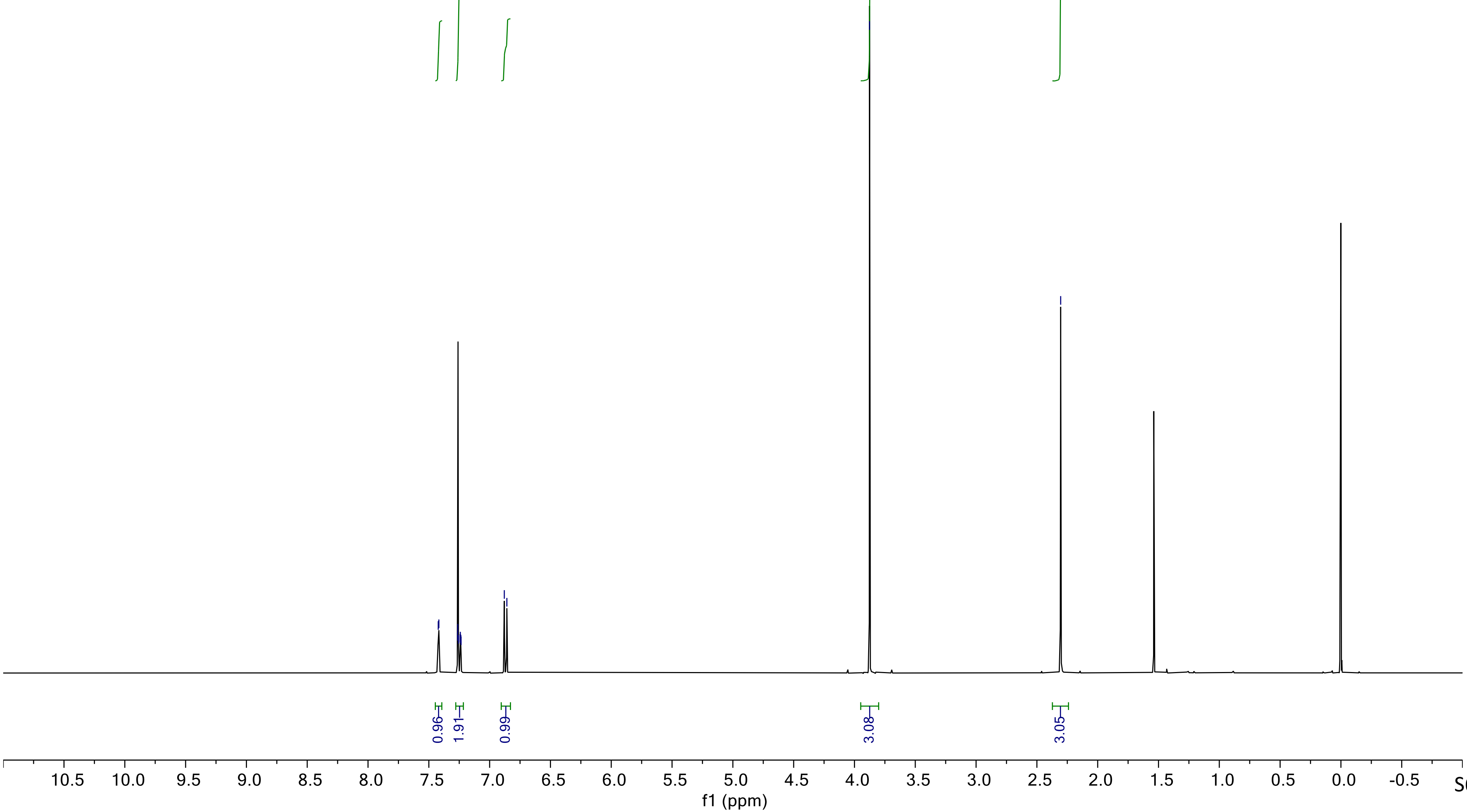

$^{13}C\{^{1}H\}$ NMR (101 MHz, $CDCl_3$)

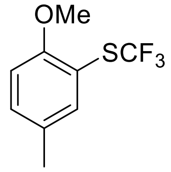

6b

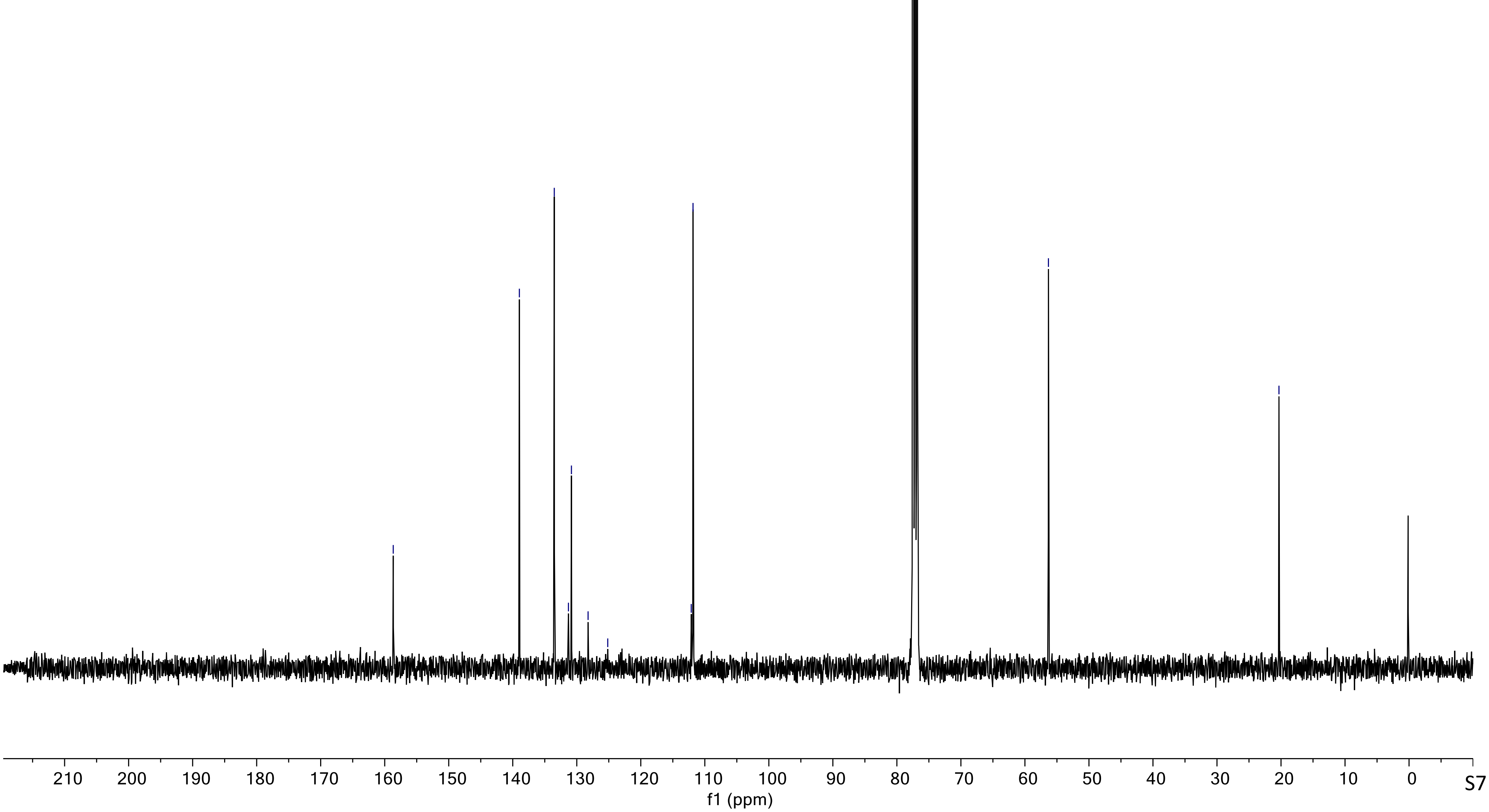

^{1}H NMR (400 MHz, $CDCl_3$)

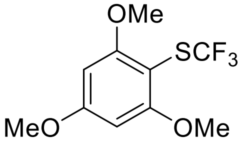

6c

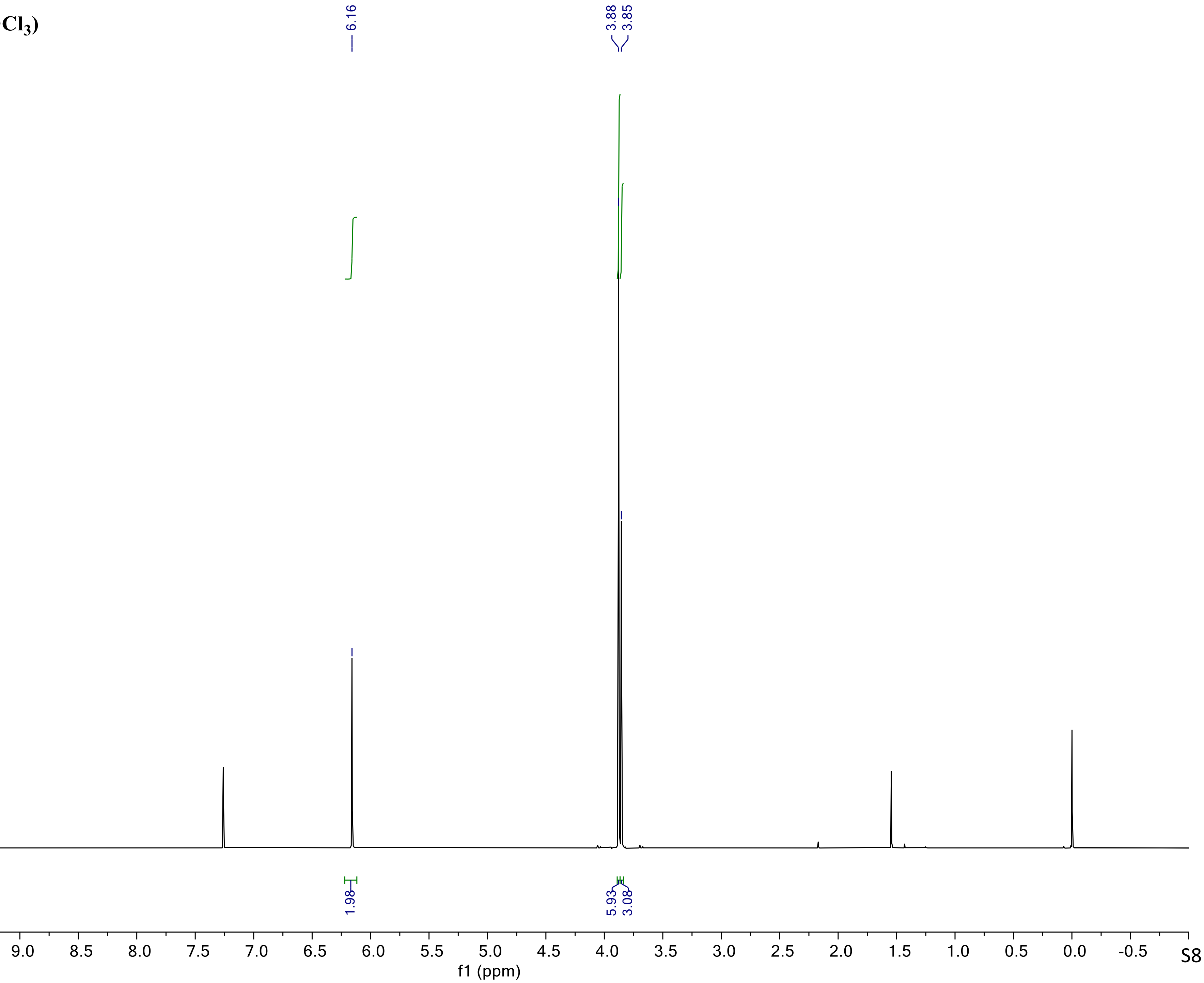

$^{13}C\{^1H\}$ NMR (101 MHz, $CDCl_3$)

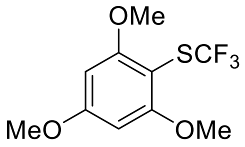

6c

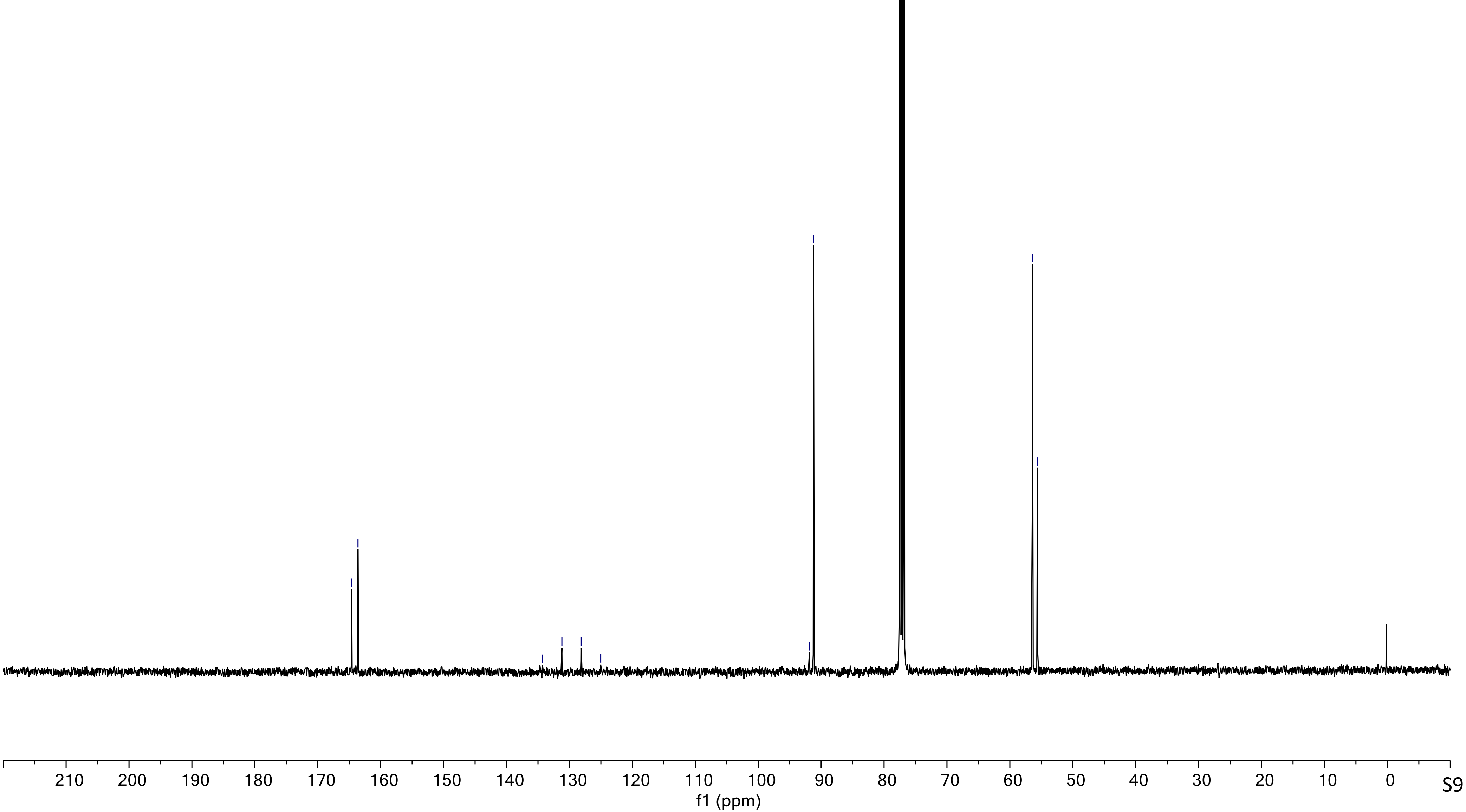

^{1}H NMR (400 MHz, $CDCl_3$)

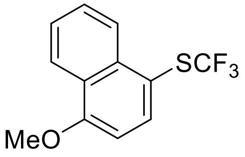

6d

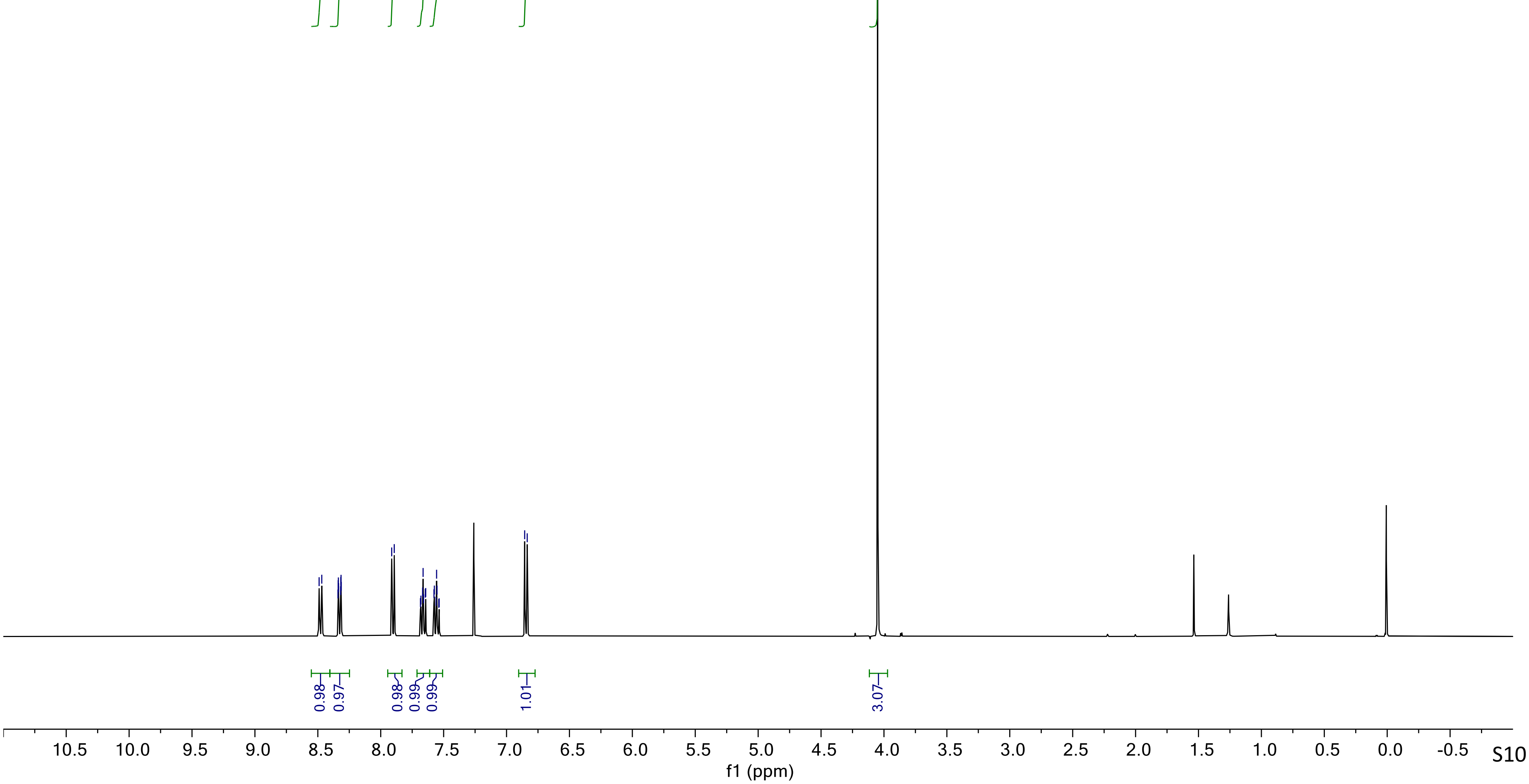

$^{13}C\{^{1}H\}$ NMR (101 MHz, $CDCl_3$)

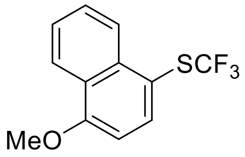

6d

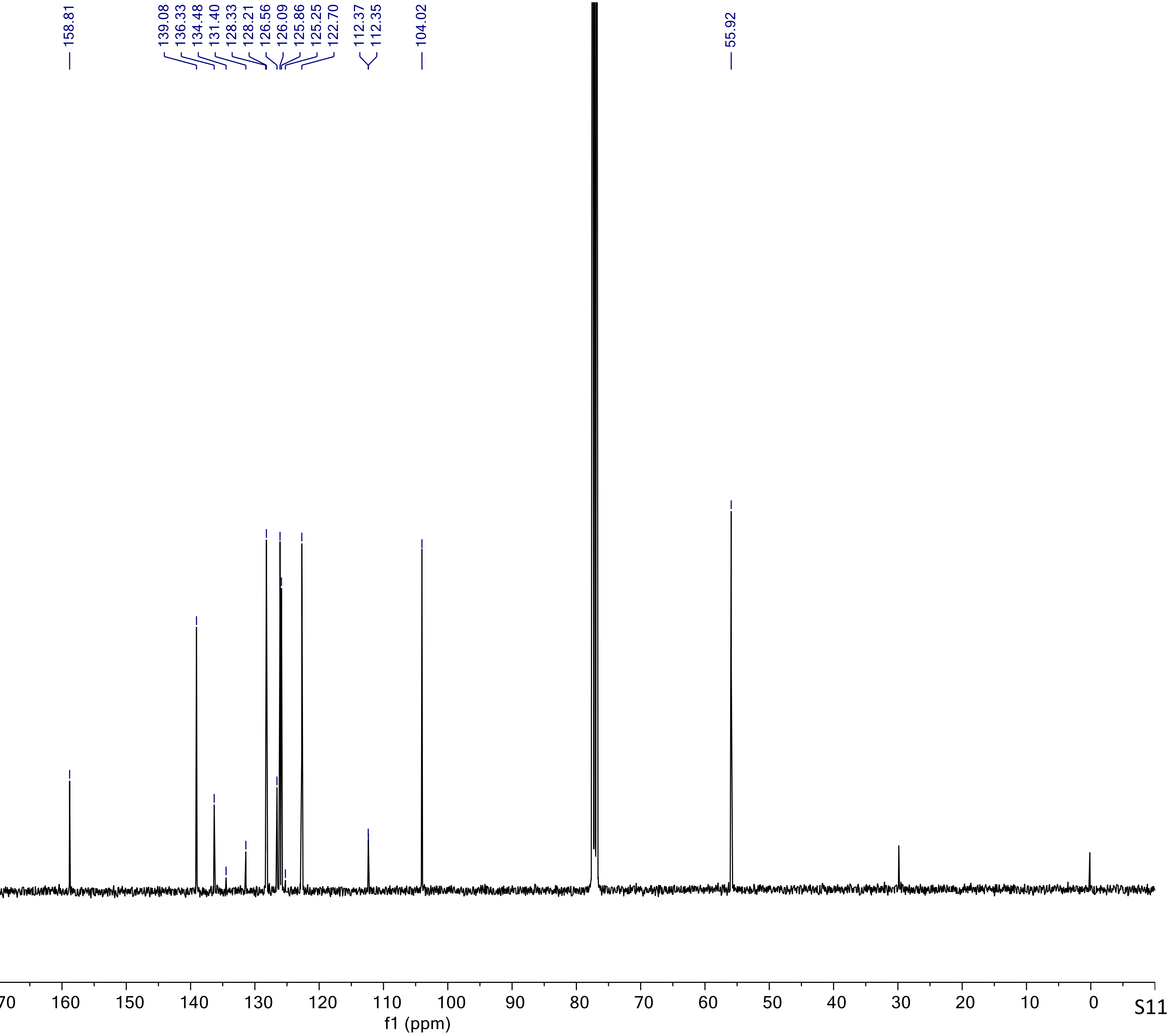

^{1}H NMR (400 MHz, $CDCl_3$)

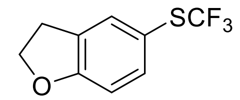

6e

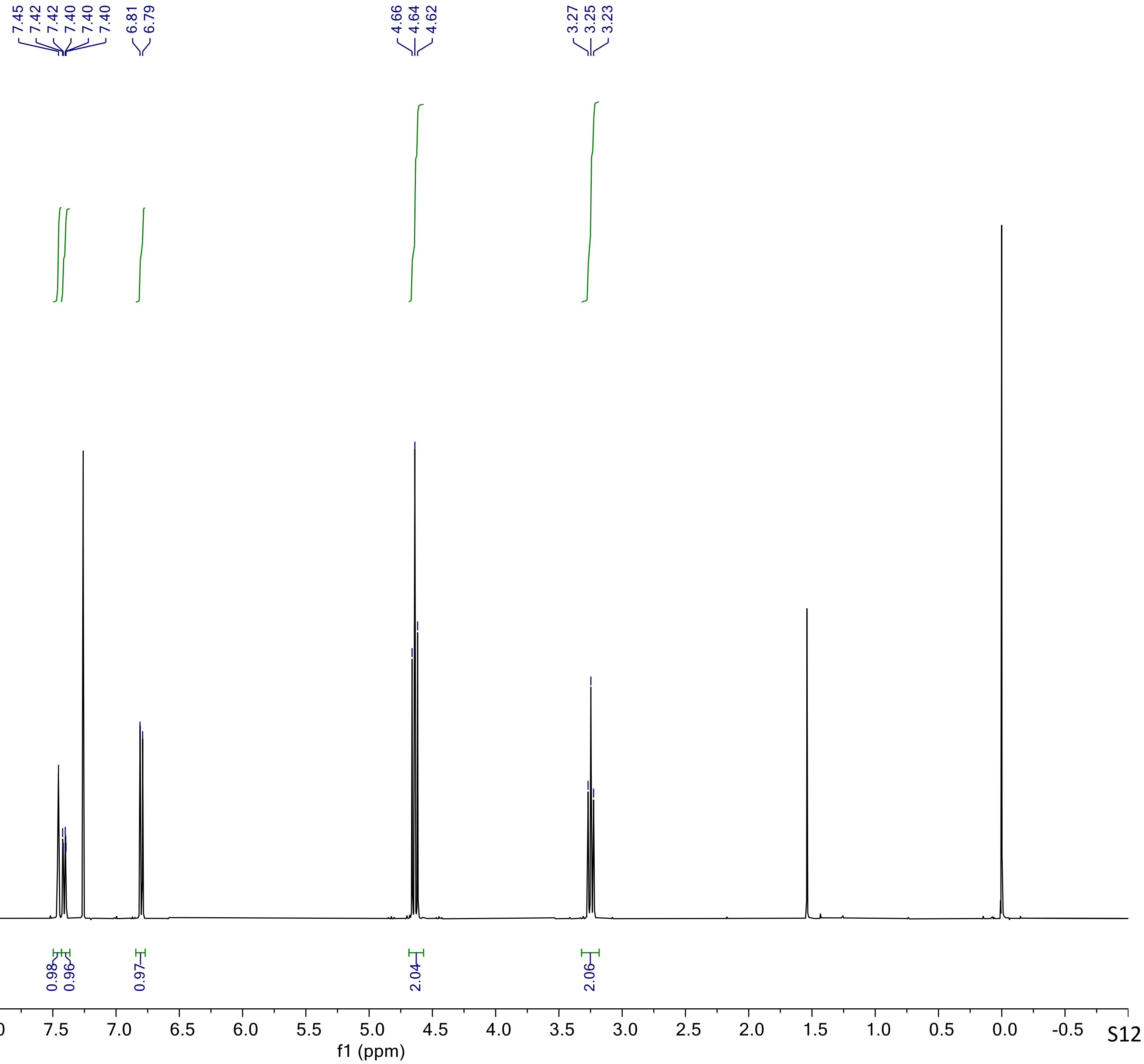

$^{13}C\{^{1}H\}$ NMR (101 MHz, $CDCl_3$)

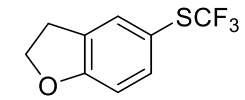

6e

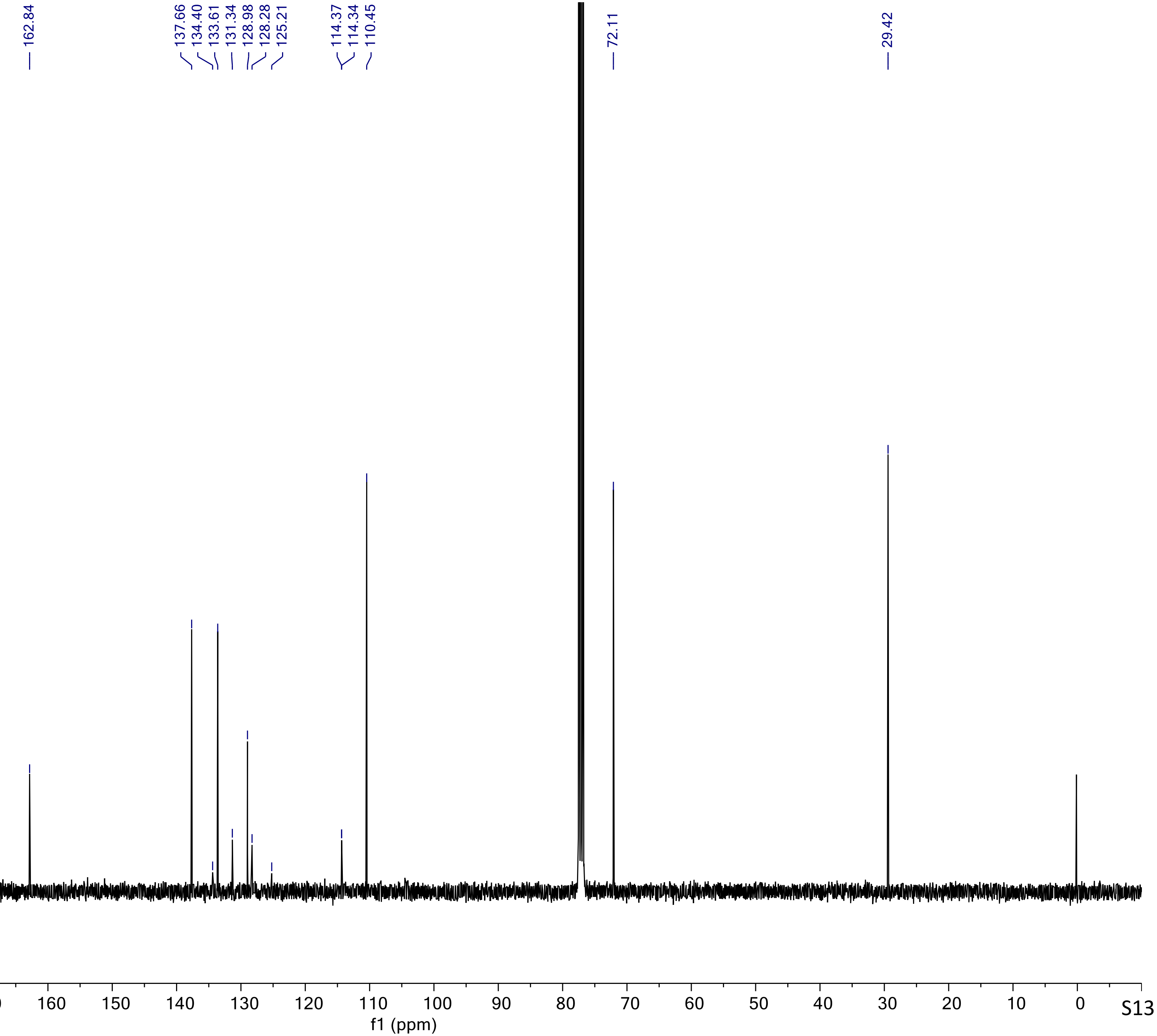

^{1}H NMR (400 MHz, CDCl$_3$)

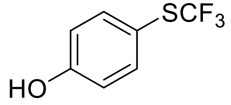

6f

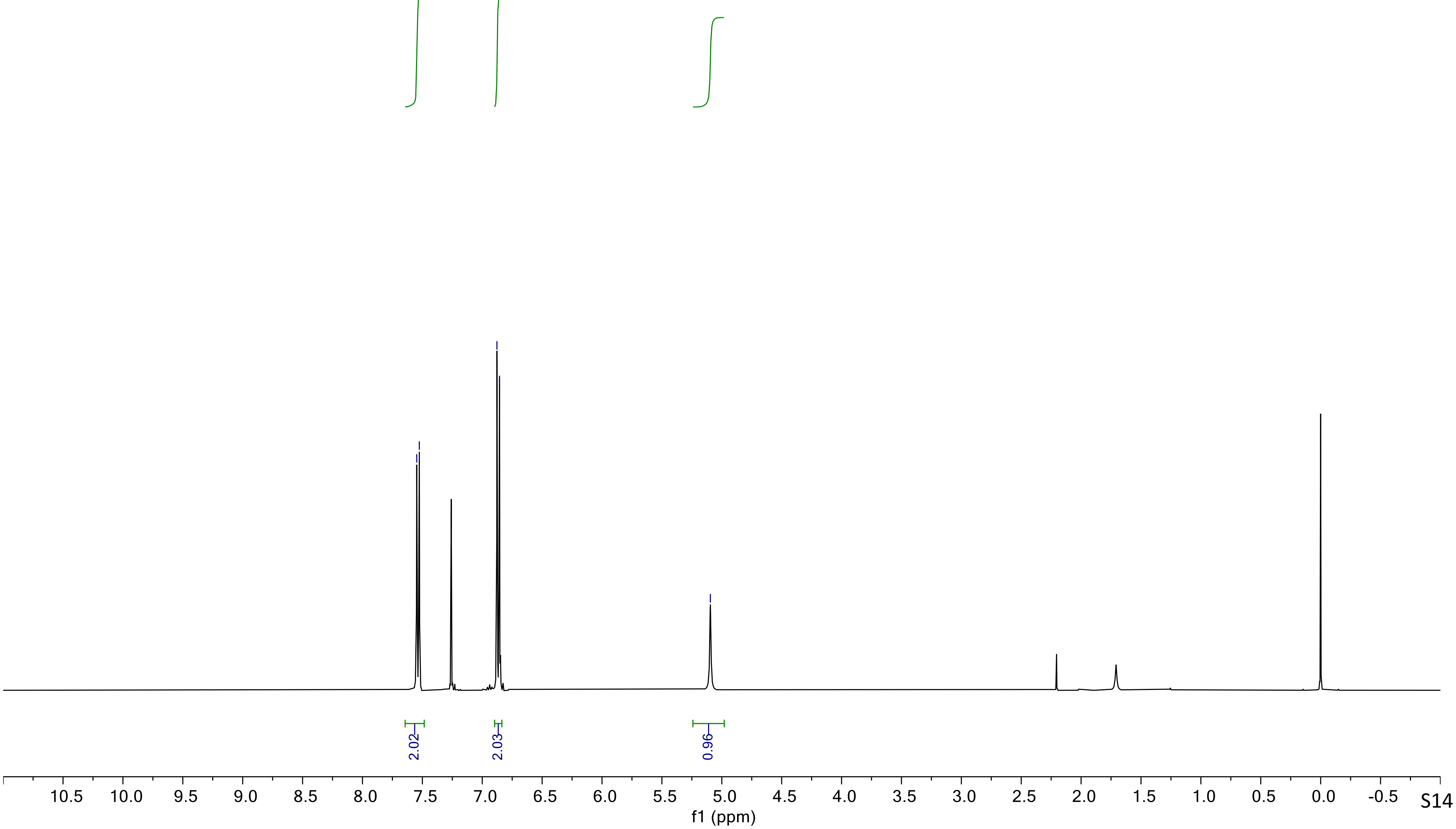

$^{13}C\{^{1}H\}$ NMR (101 MHz, $CDCl_3$)

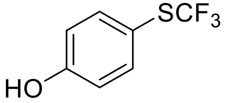

6f

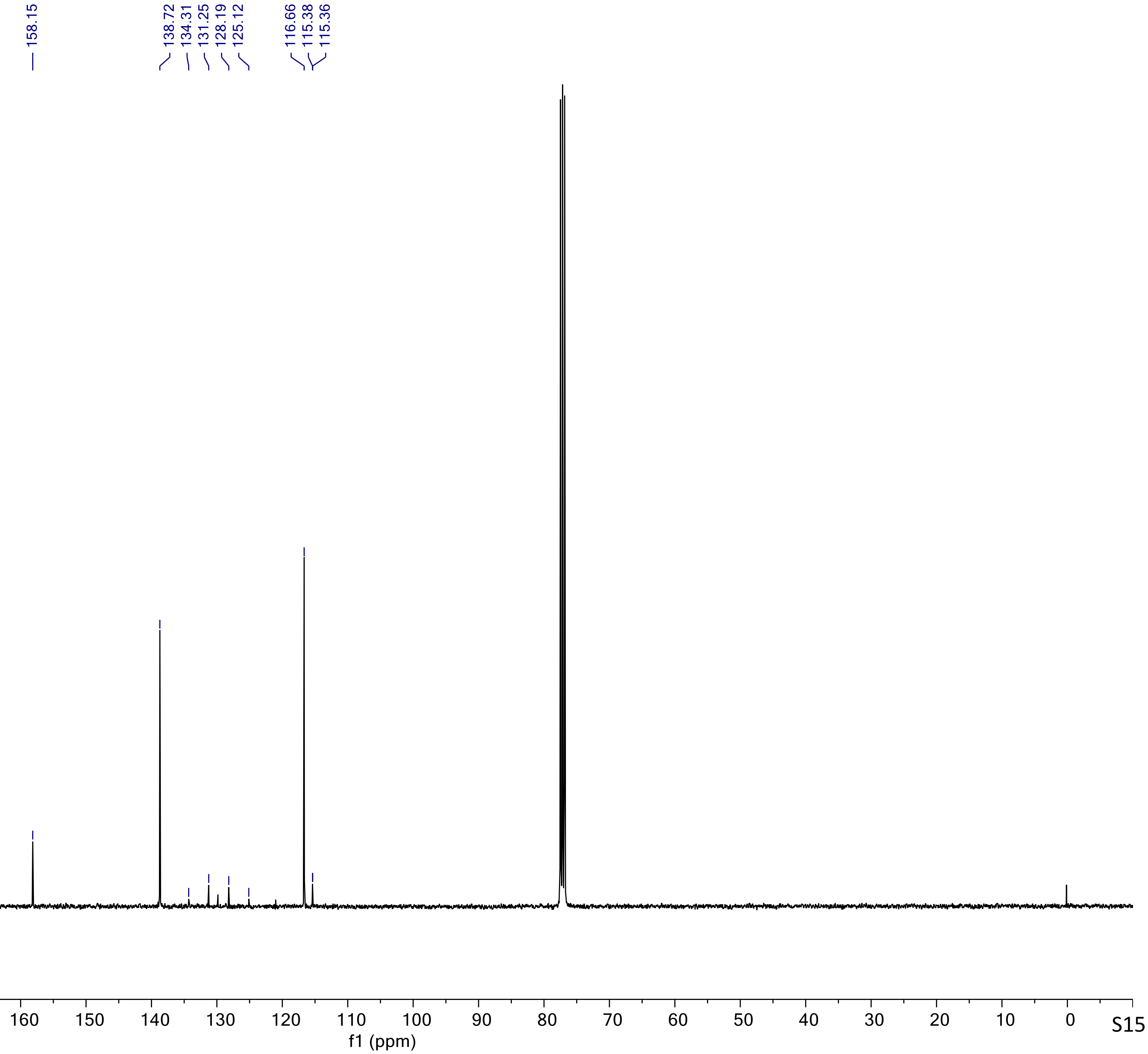

^{1}H NMR (400 MHz, $CDCl_3$)

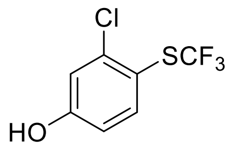

6g

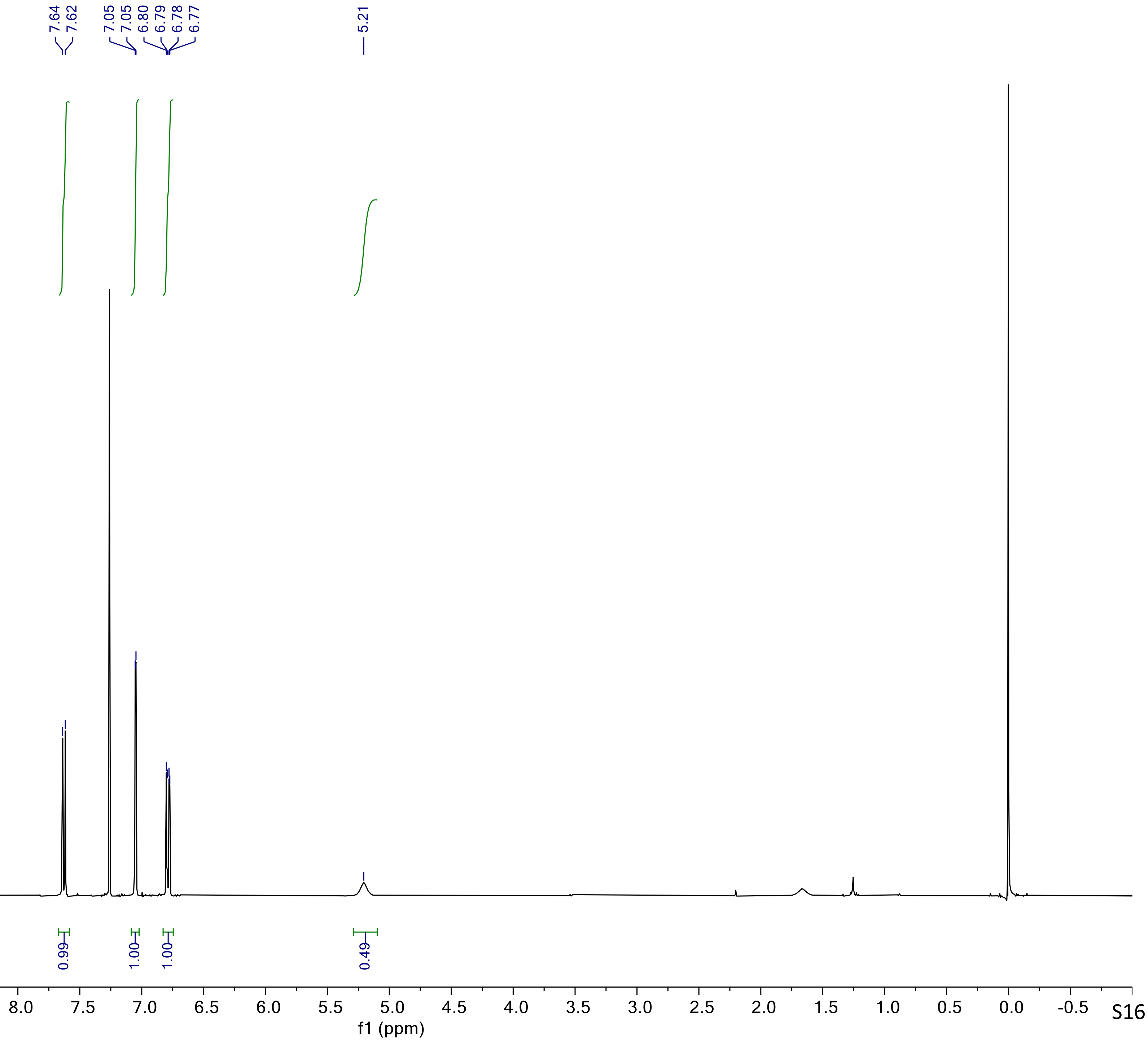

$^{13}C\{^1H\}$ NMR (101 MHz, $CDCl_3$)

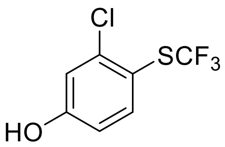

6g

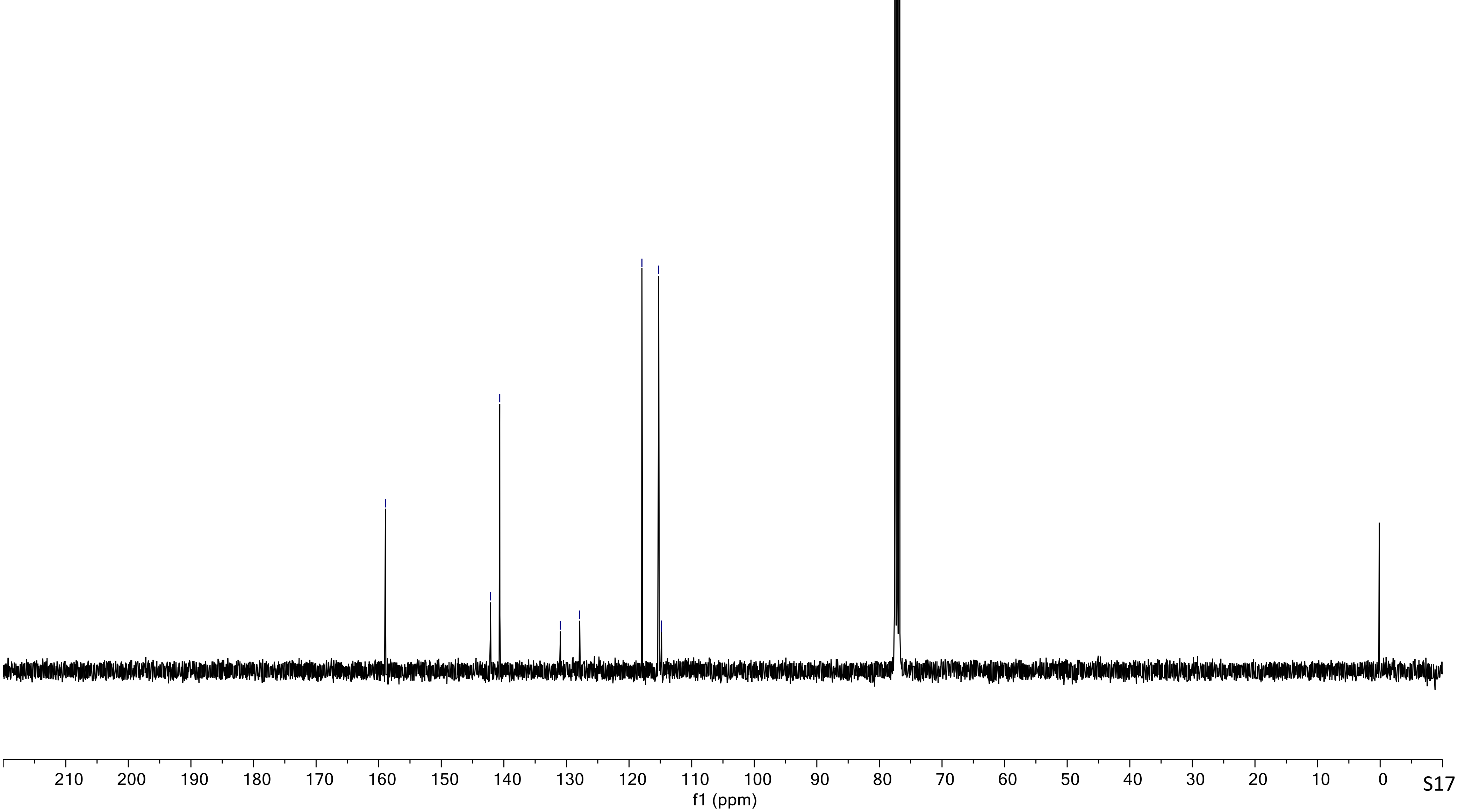

^{1}H NMR (400 MHz, $CDCl_3$)

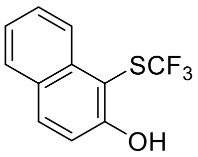

6h

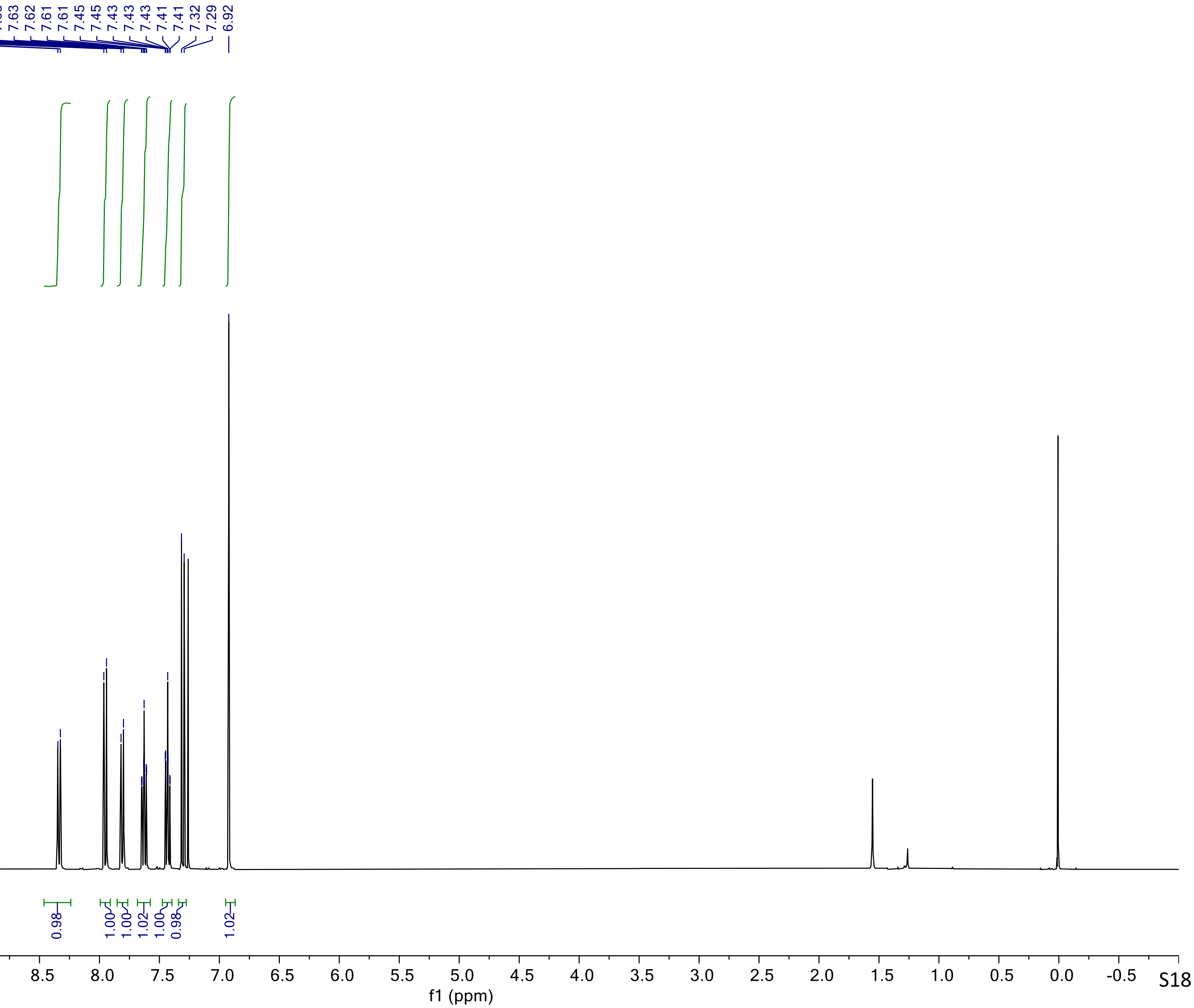

$^{13}C\{^{1}H\}$ NMR (101 MHz, $CDCl_3$)

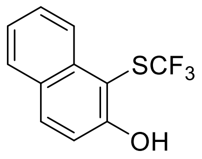

6h

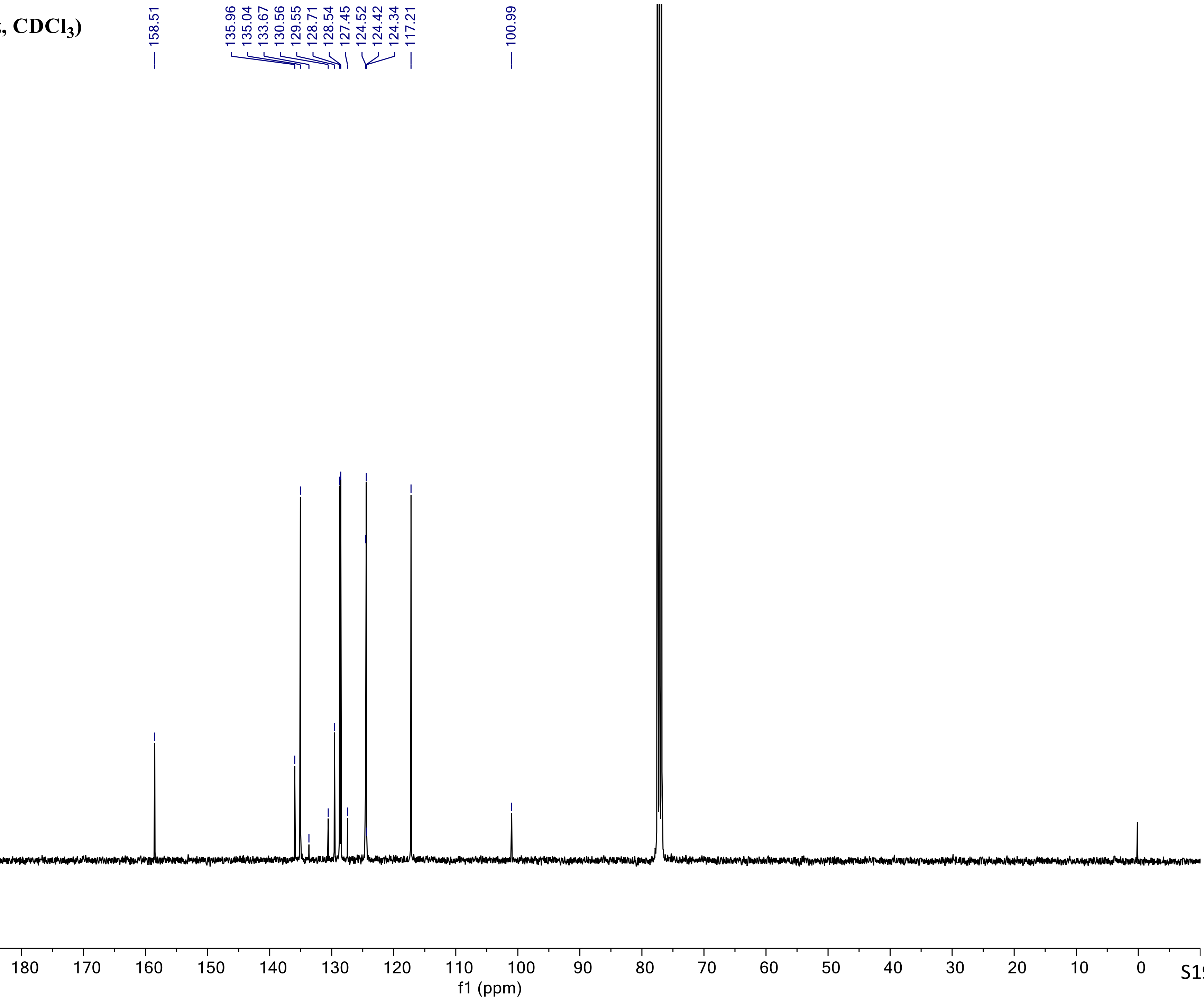

^{1}H NMR (400 MHz, $CDCl_3$)

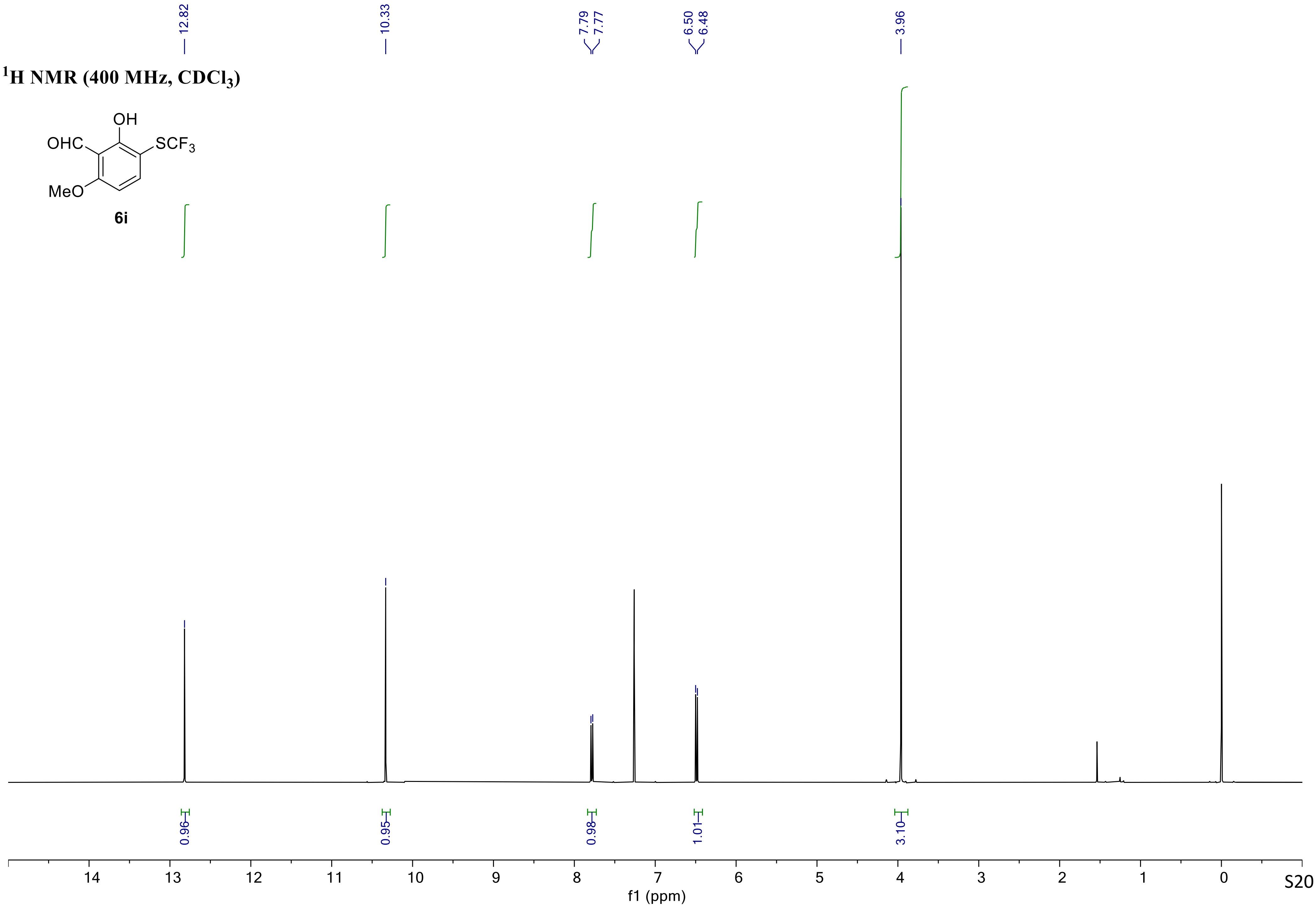

$^{13}C\{^{1}H\}$ NMR (101 MHz, $CDCl_3$)

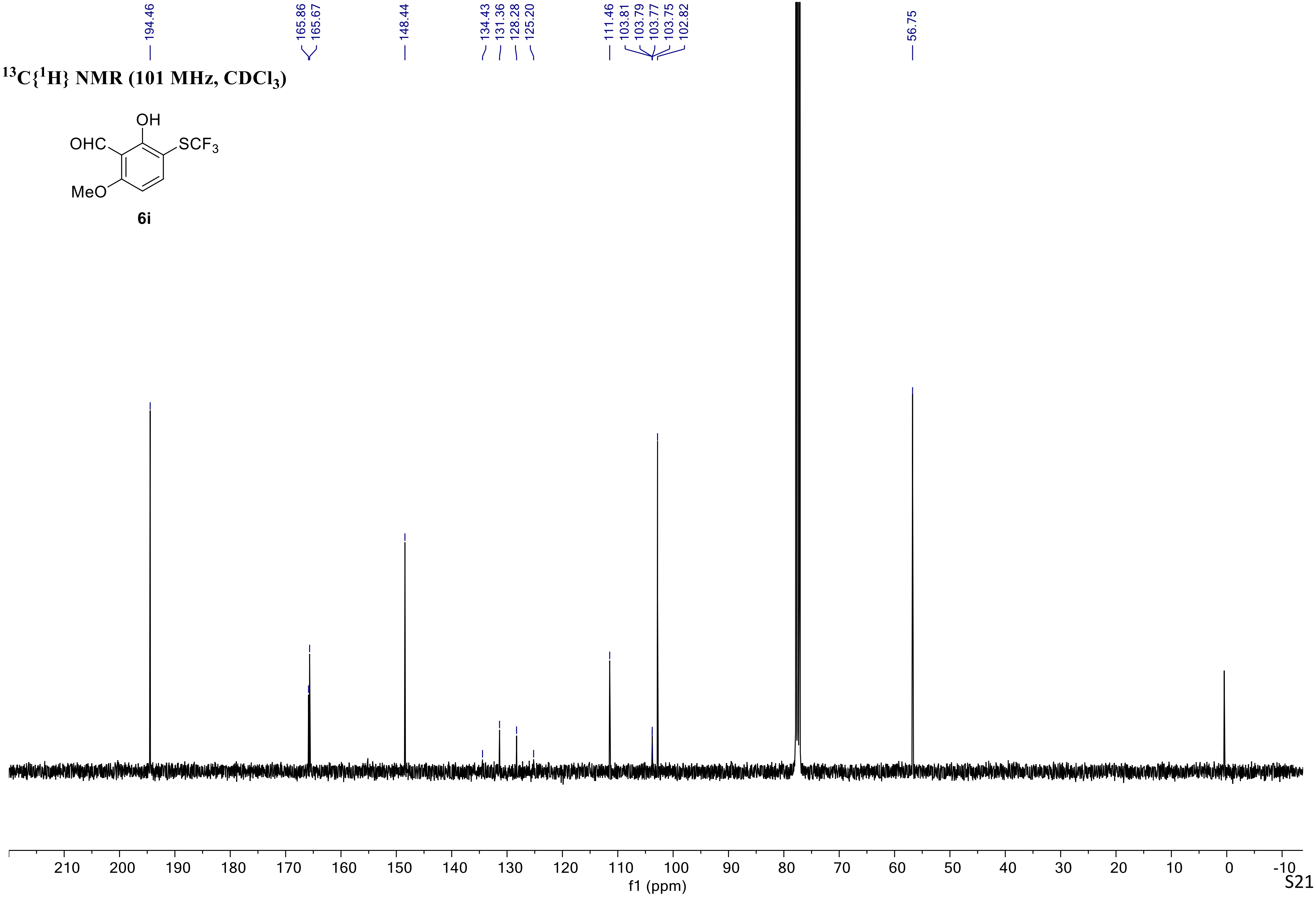

6i

^{19}F NMR (376 MHz, CDCl$_3$)

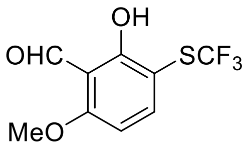

6i

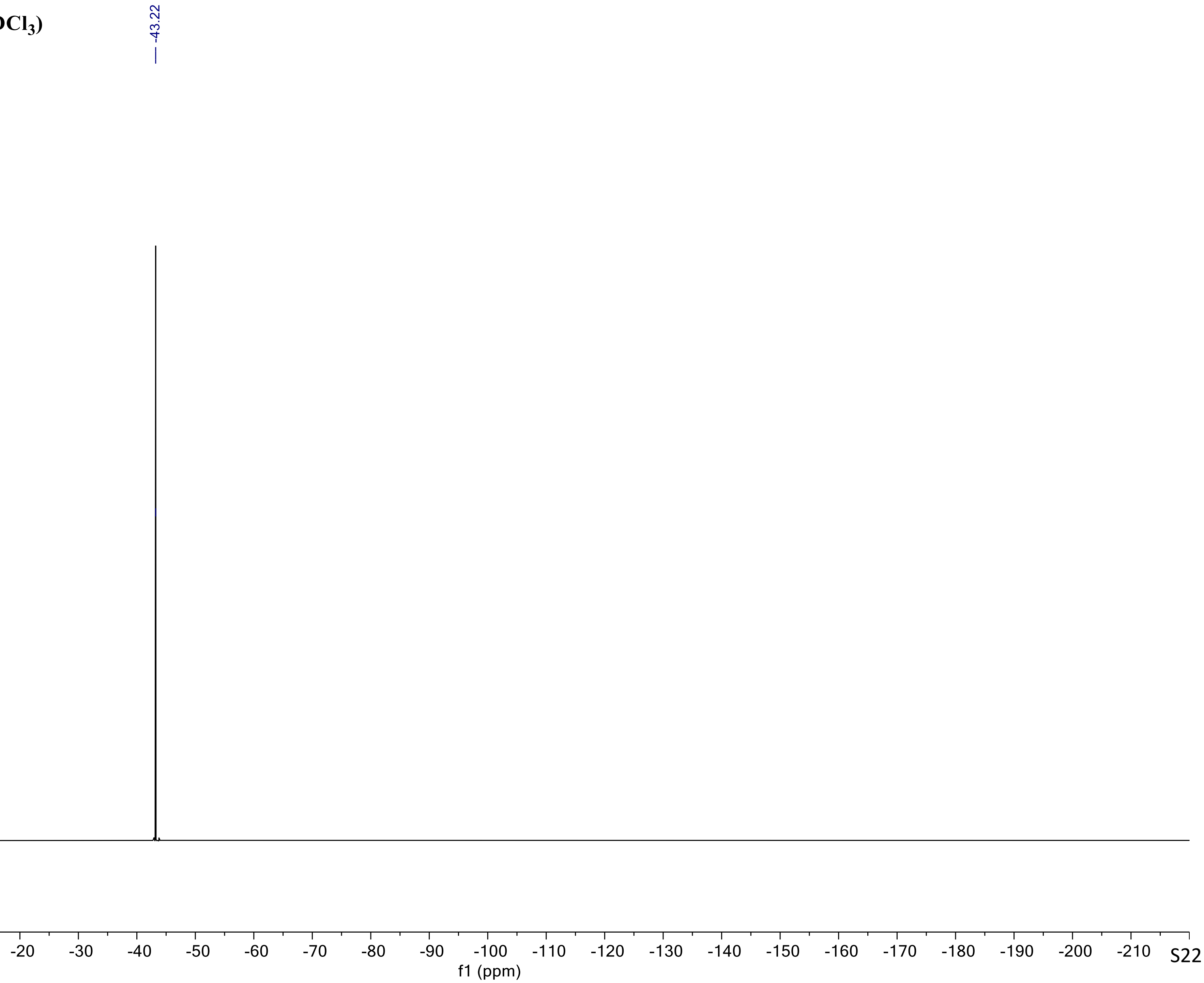

^{1}H NMR (400 MHz, $CDCl_3$)

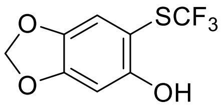

6j

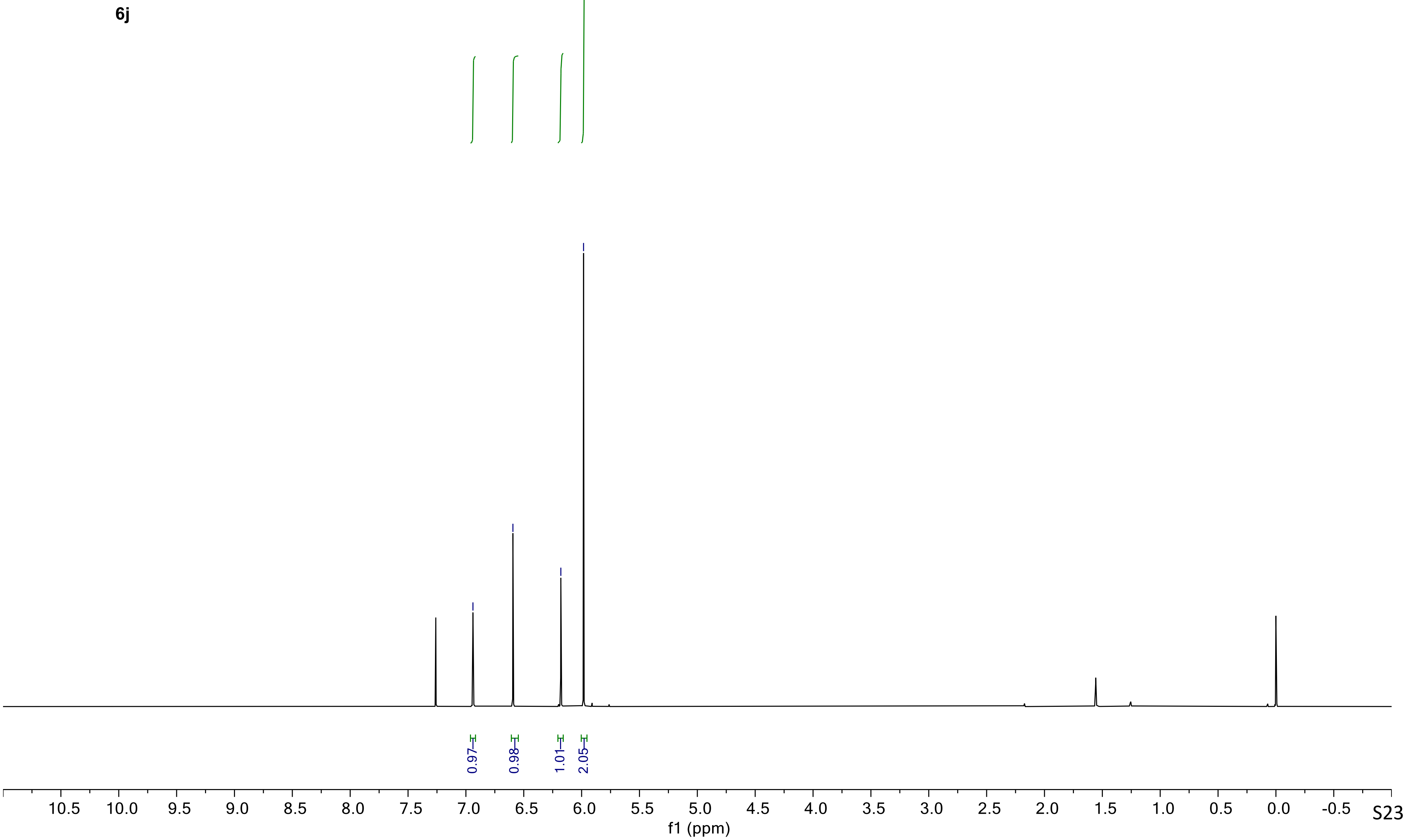

$^{13}C\{^1H\}$ NMR (101 MHz, $CDCl_3$)

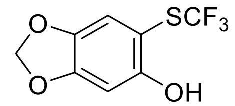

6j

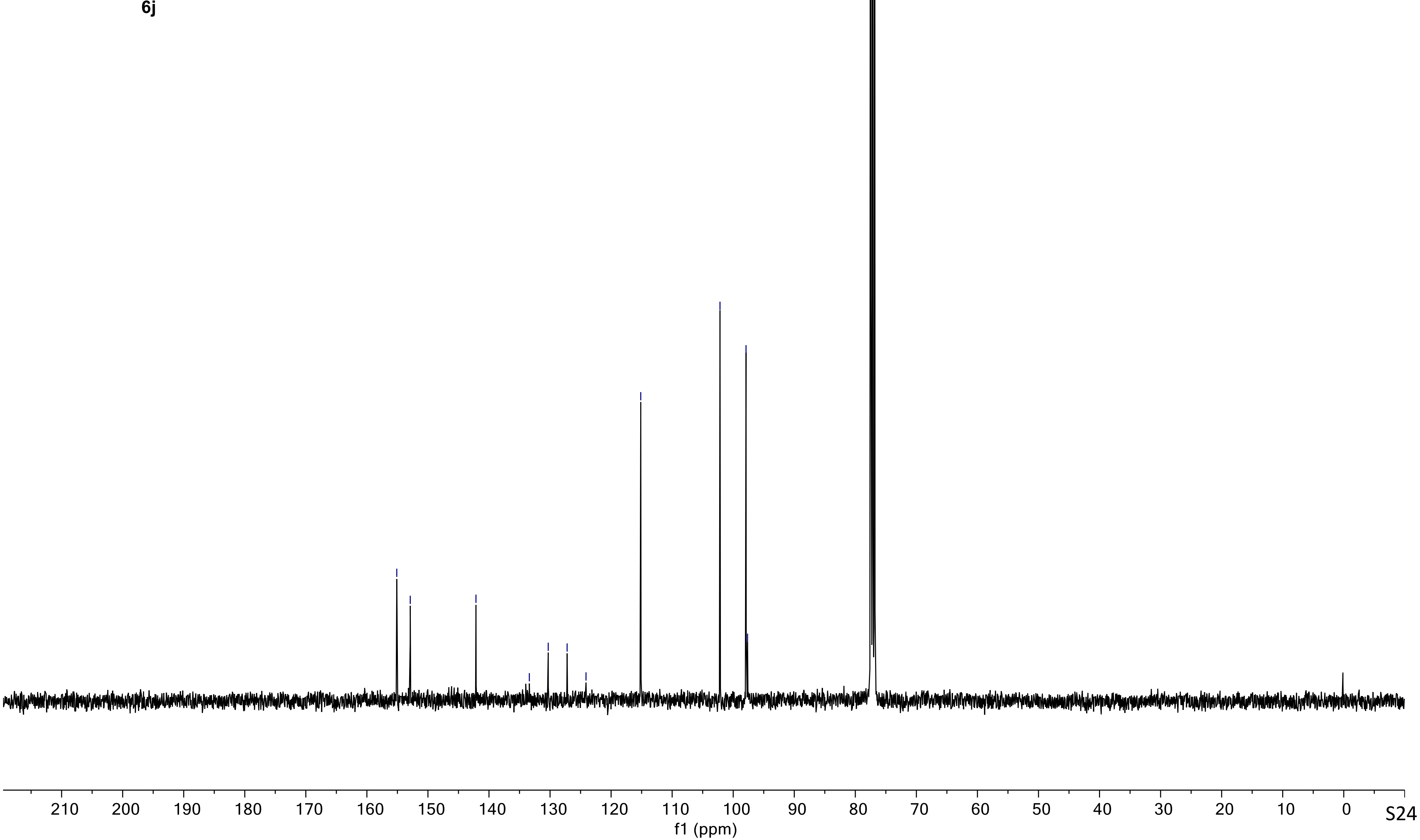

^{1}H NMR (400 MHz, $CDCl_3$)

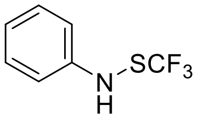

6k

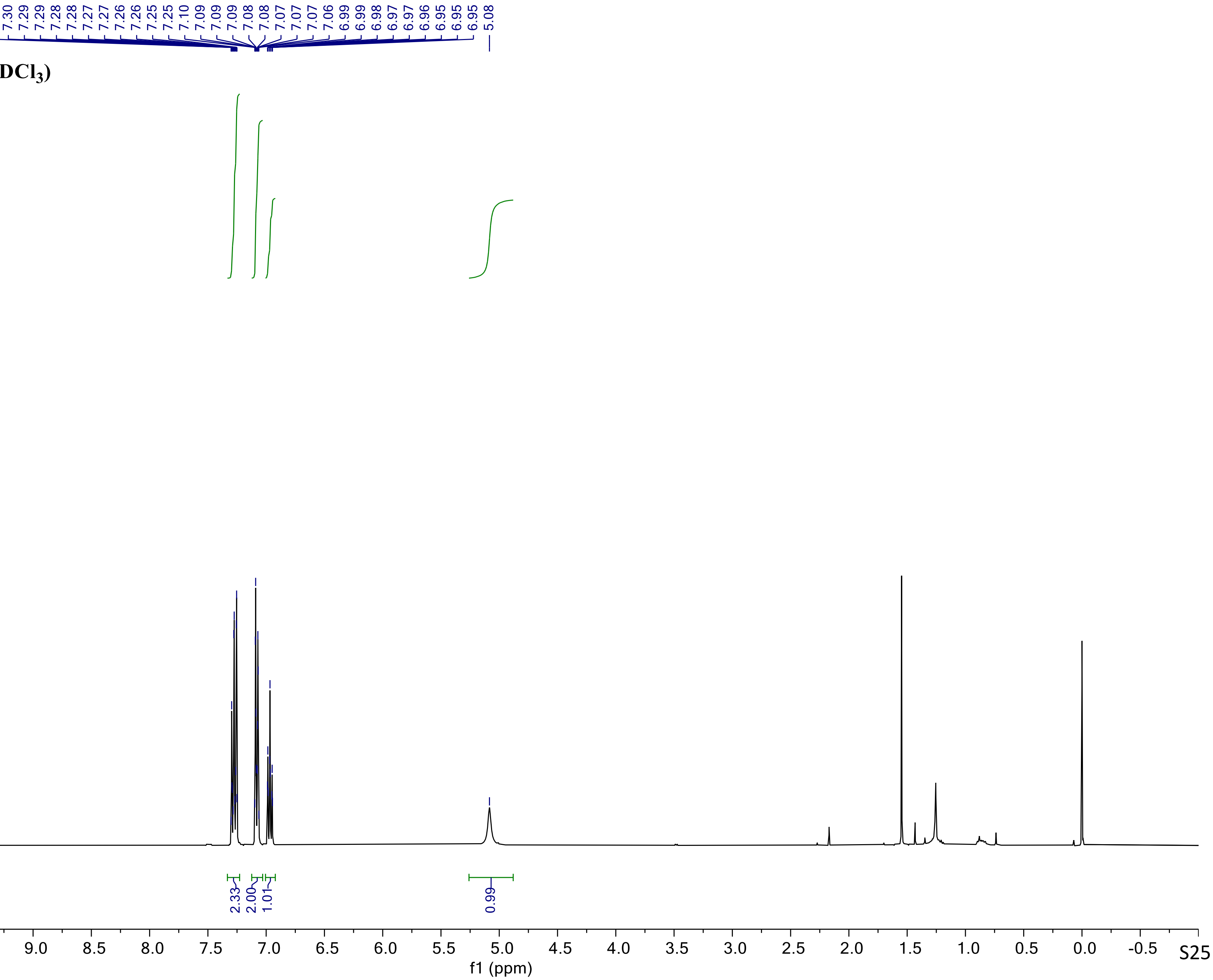

$^{13}C\{^1H\}$ NMR (101 MHz, $CDCl_3$)

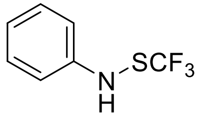

6k

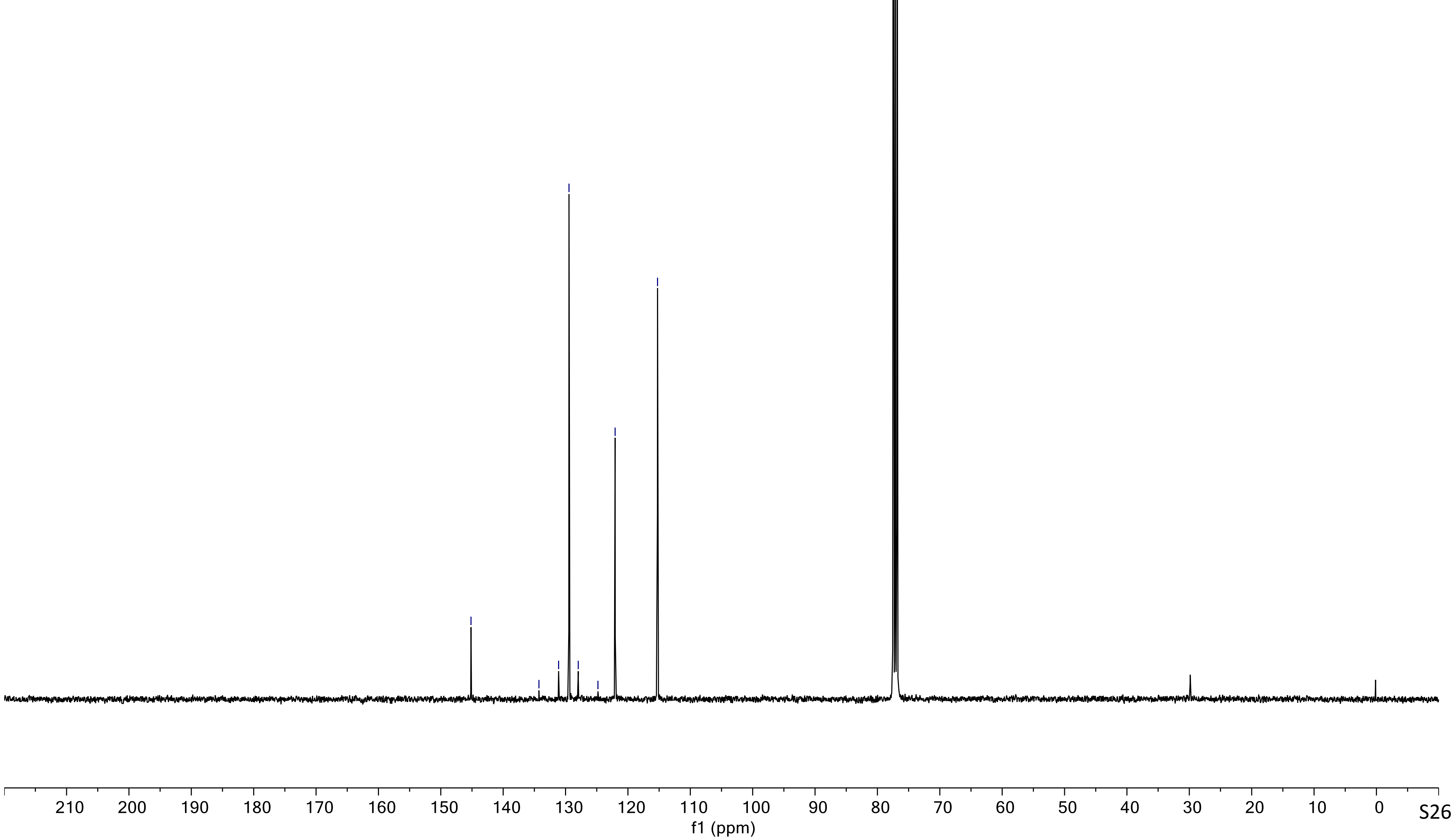

^{1}H NMR (400 MHz, $CDCl_3$)

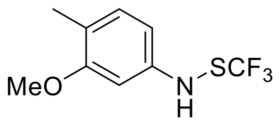

6l

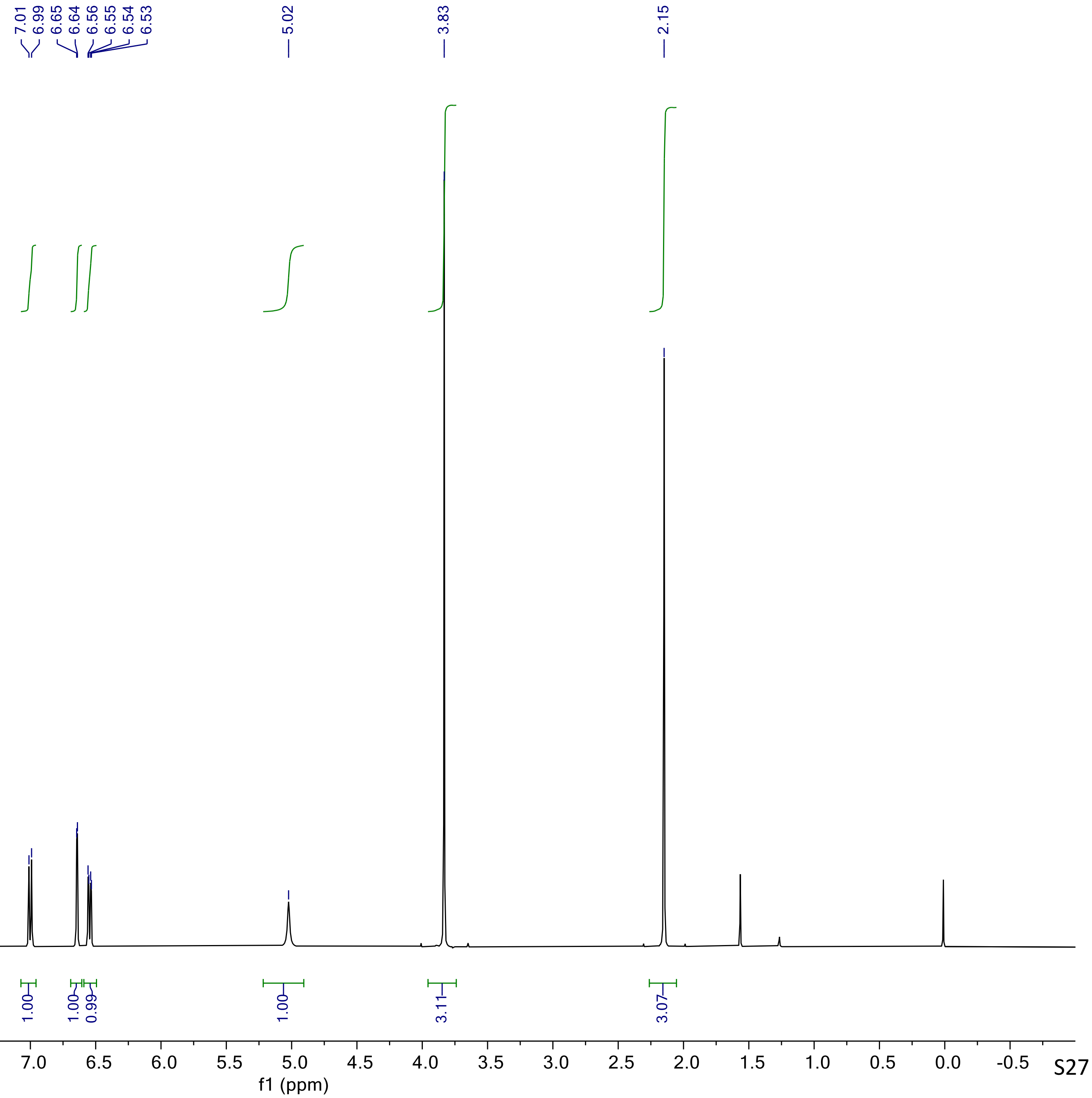

$^{13}C\{^1H\}$ NMR (101 MHz, $CDCl_3$)

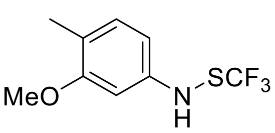

6l

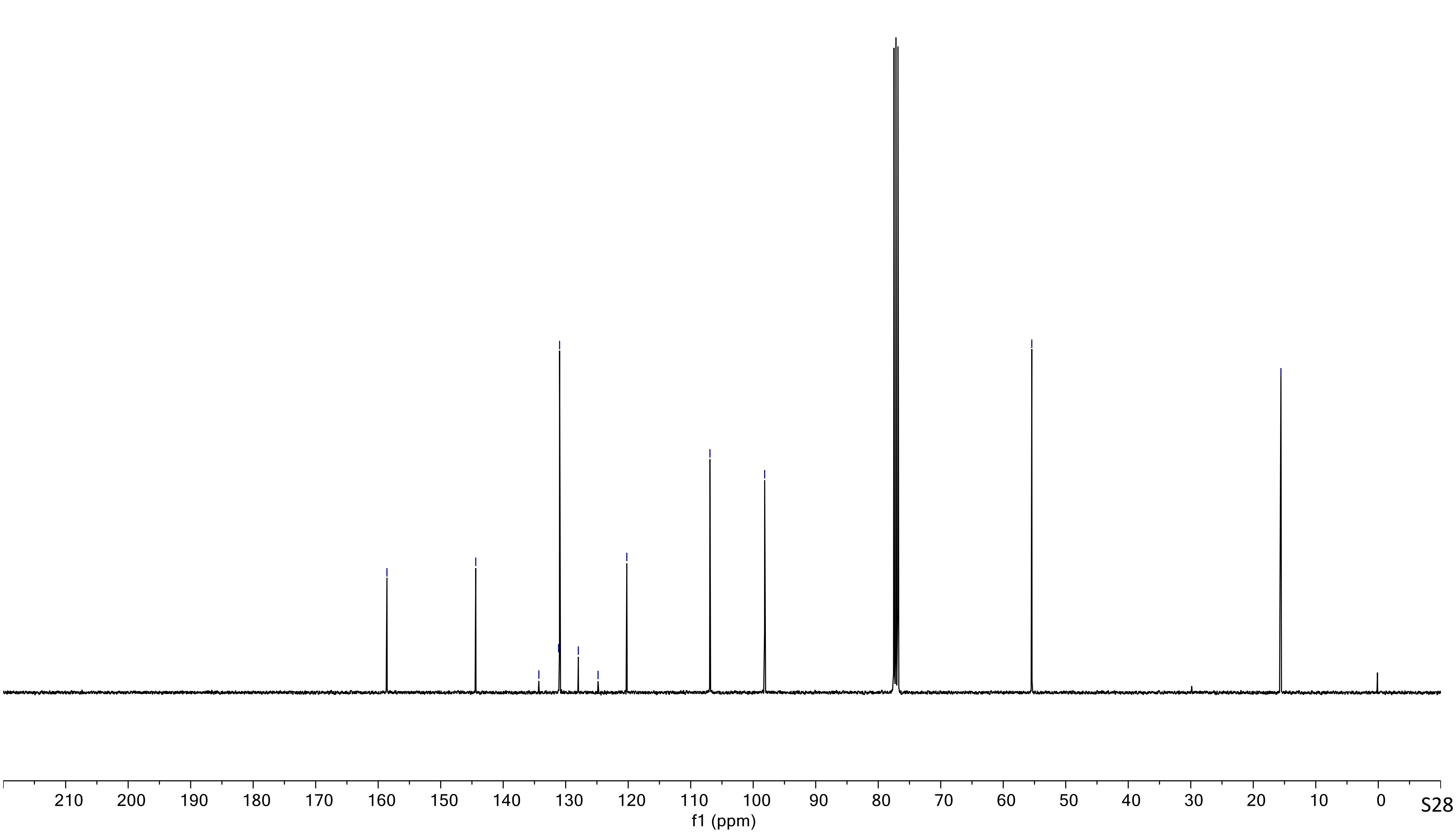

^{19}F NMR (376 MHz, CDCl$_3$)

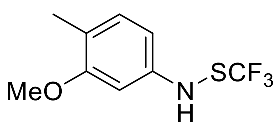

6l

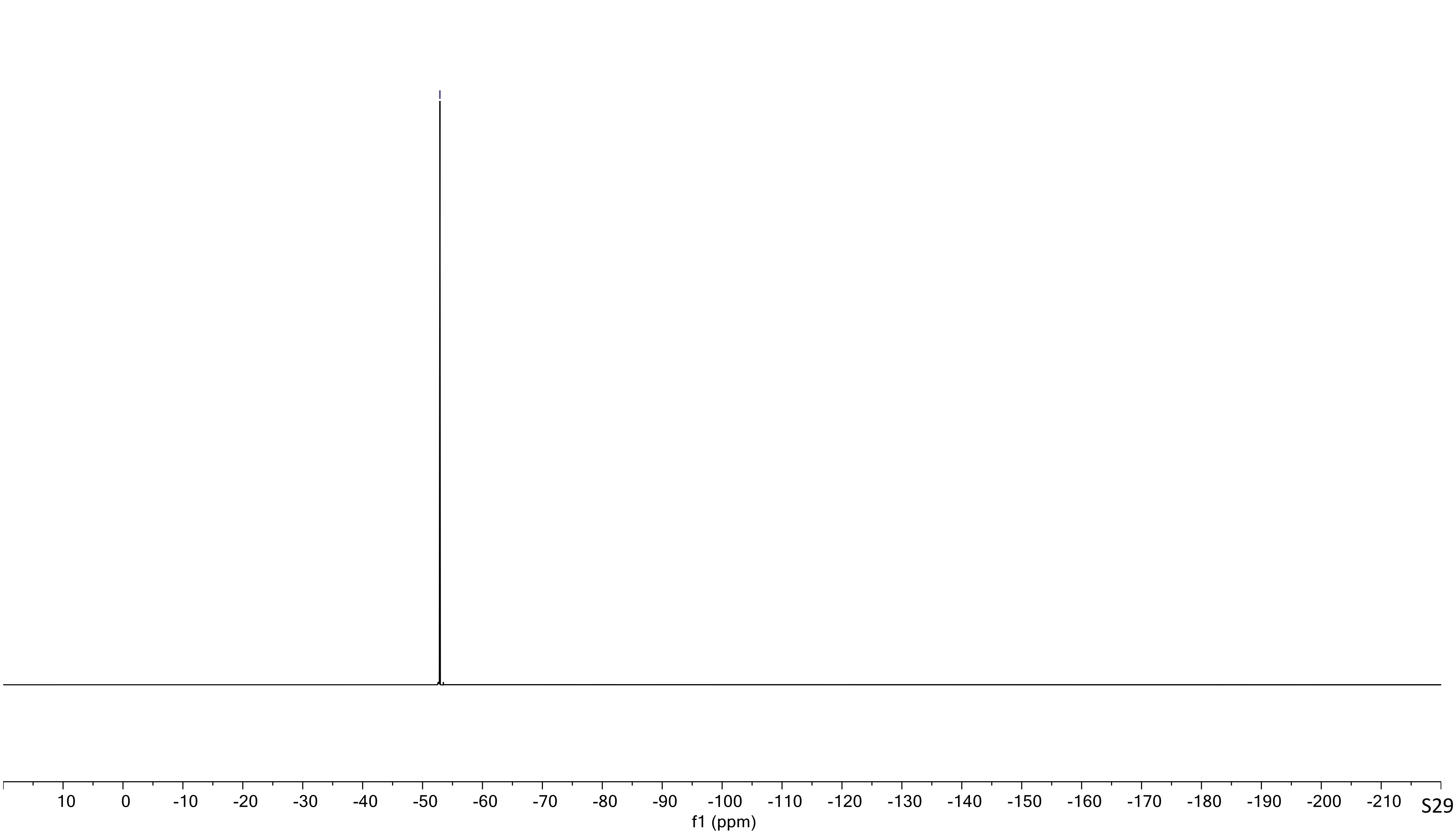

^{1}H NMR (400 MHz, $CDCl_3$)

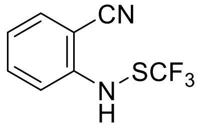

6m

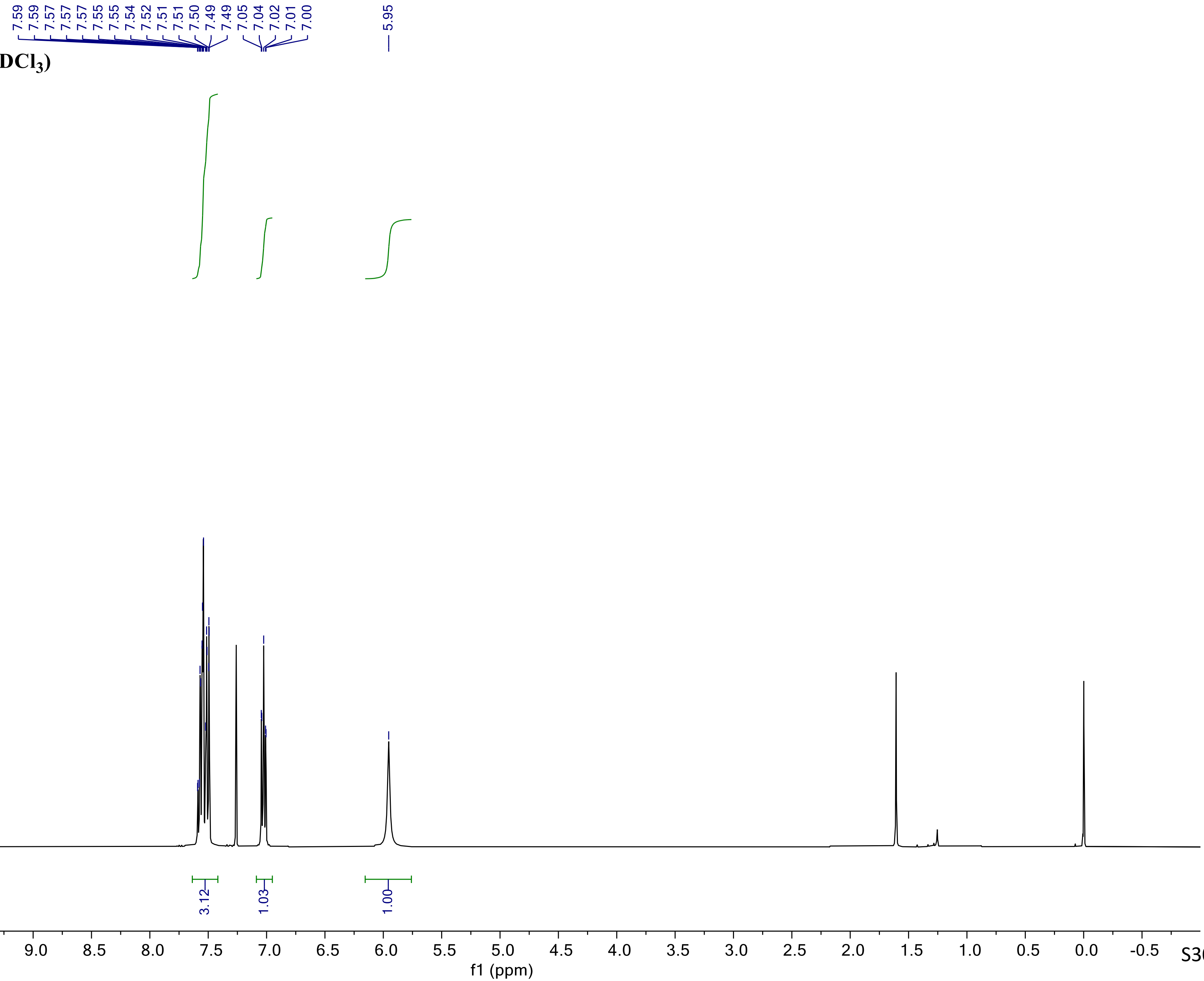

$^{13}C\{^{1}H\}$ NMR (101 MHz, $CDCl_3$)

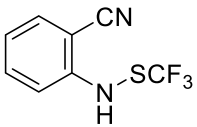

6m

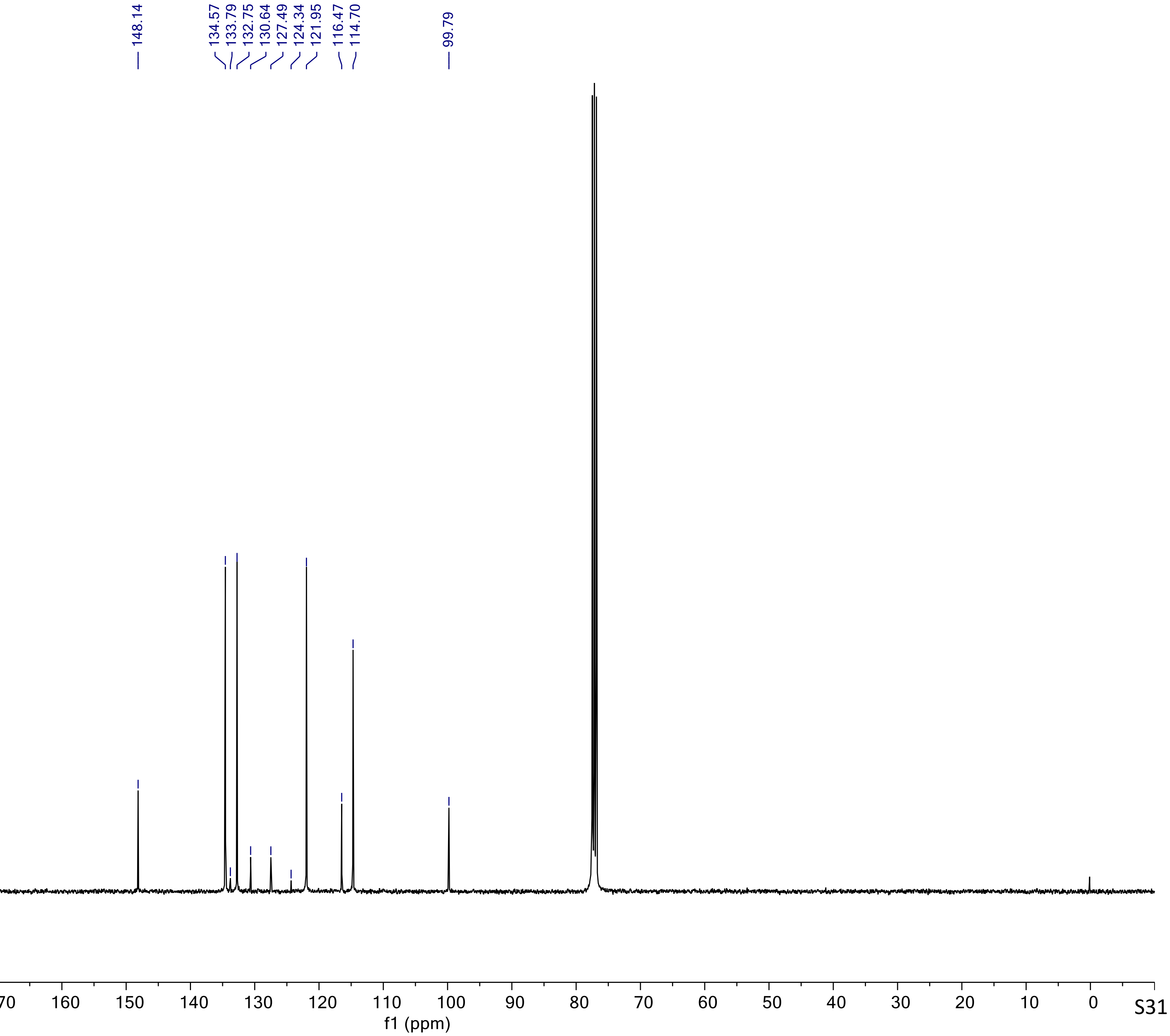

^{19}F NMR (376 MHz, $CDCl_3$)

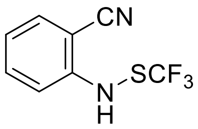

6m

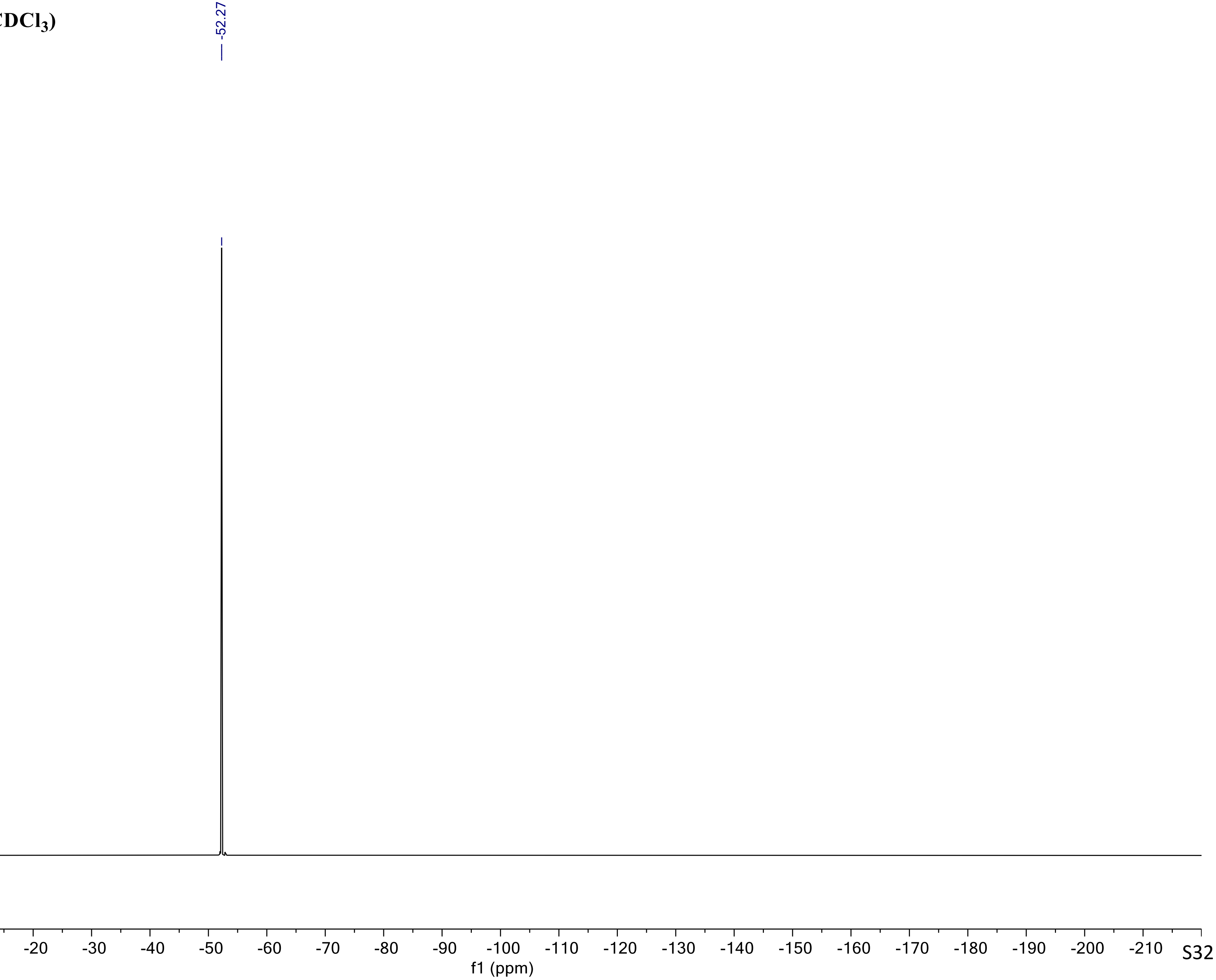

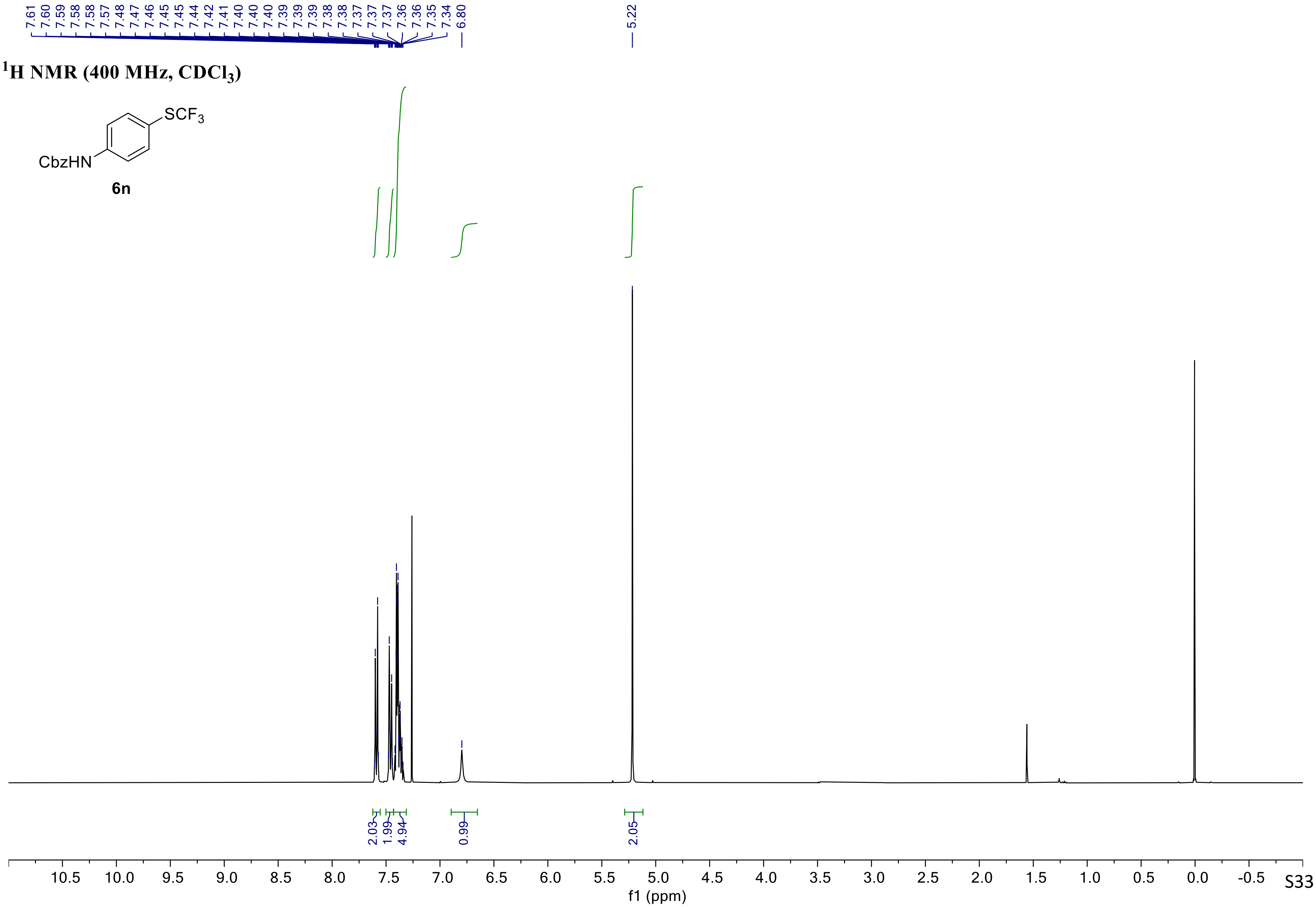
7.61
7.60
7.59
7.58
7.58
7.57
7.48
7.47
7.46
7.45
7.45
7.44
7.42
7.41
7.40
7.40
7.40
7.39
7.39
7.39
7.38
7.38
7.37
7.37
7.37
7.36
7.36
7.35
7.34
6.80
5.22
^{1}H NMR (400 MHz, $CDCl_3$)
SCF_3
CbzHN
6n
2.03
1.99
4.94
0.99
2.05
10.5
10.0
9.5
9.0
8.5
8.0
7.5
7.0
6.5
6.0
5.5
5.0
4.5
4.0
3.5
3.0
2.5
2.0
1.5
1.0
0.5
0.0
-0.5
f1 (ppm)

$^{13}C\{^{1}H\}$ NMR (101 MHz, $CDCl_3$)

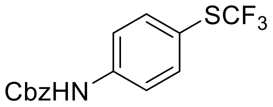

6n

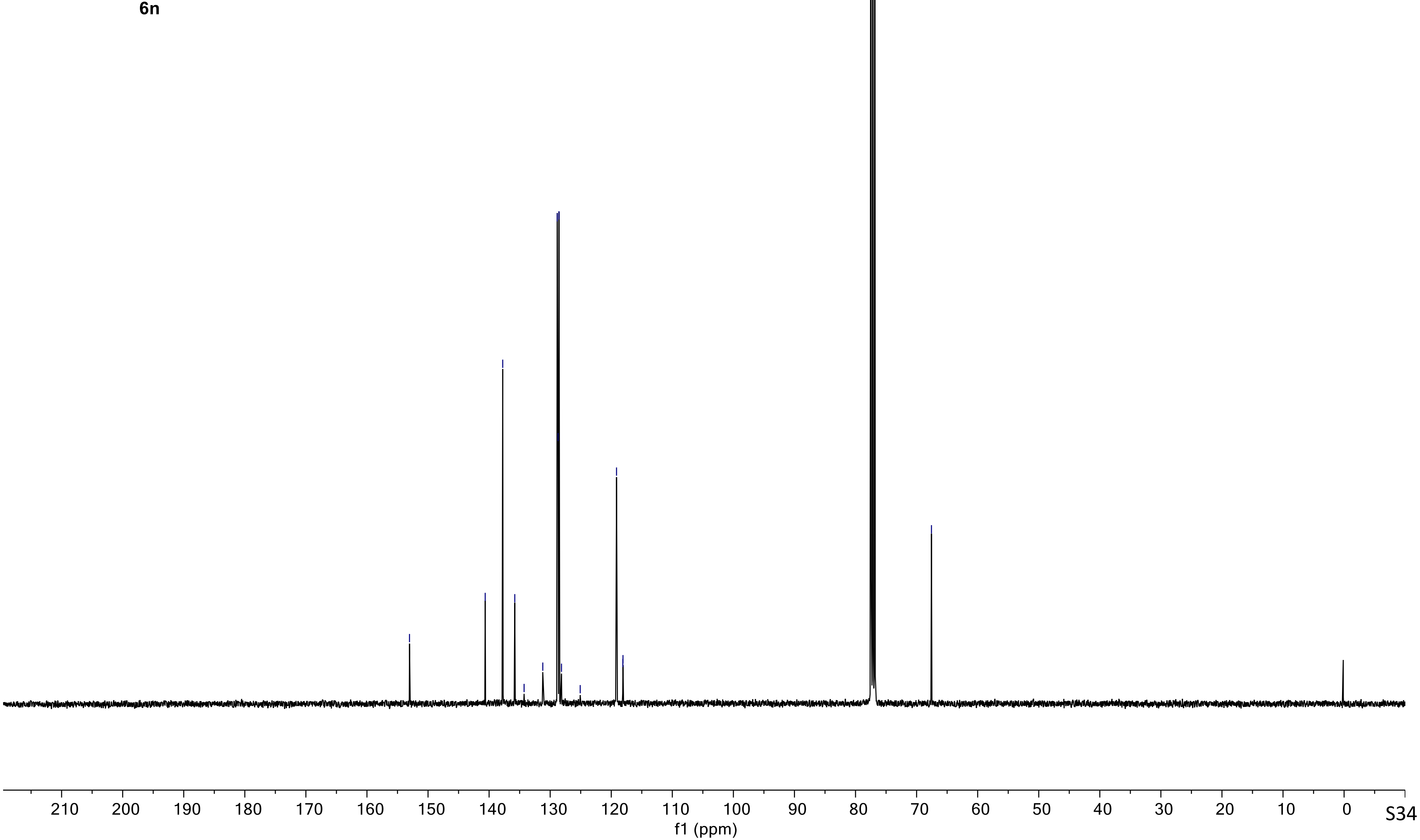

^{19}F NMR (376 MHz, CDCl$_3$)

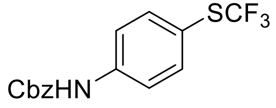

6n

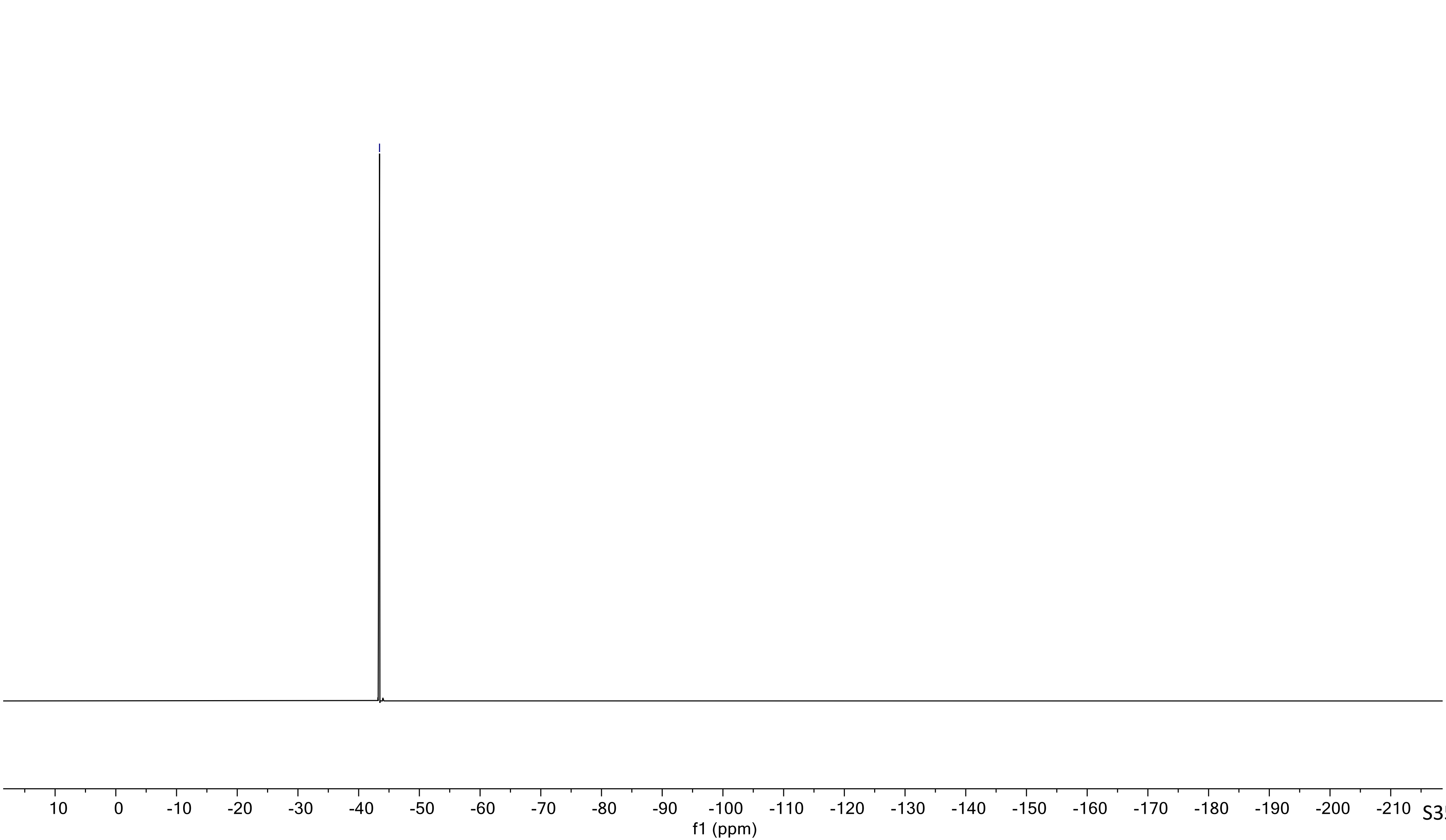

^{1}H NMR (400 MHz, CDCl3)

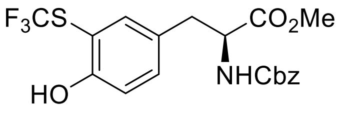

6o

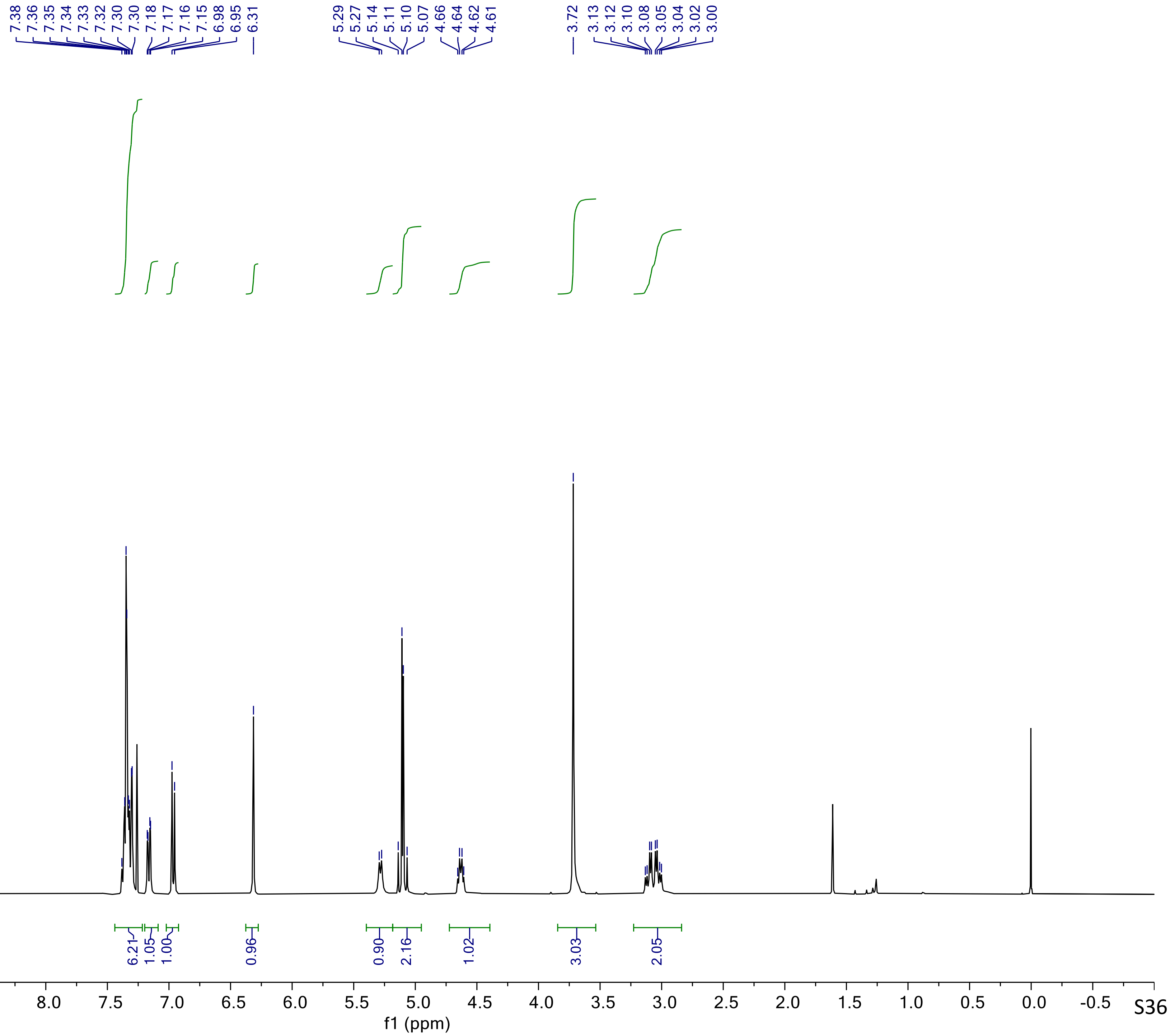

$^{13}C\{^1H\}$ NMR (101 MHz, $CDCl_3$)

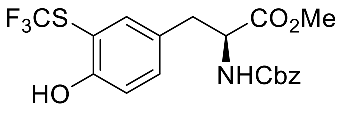

6o

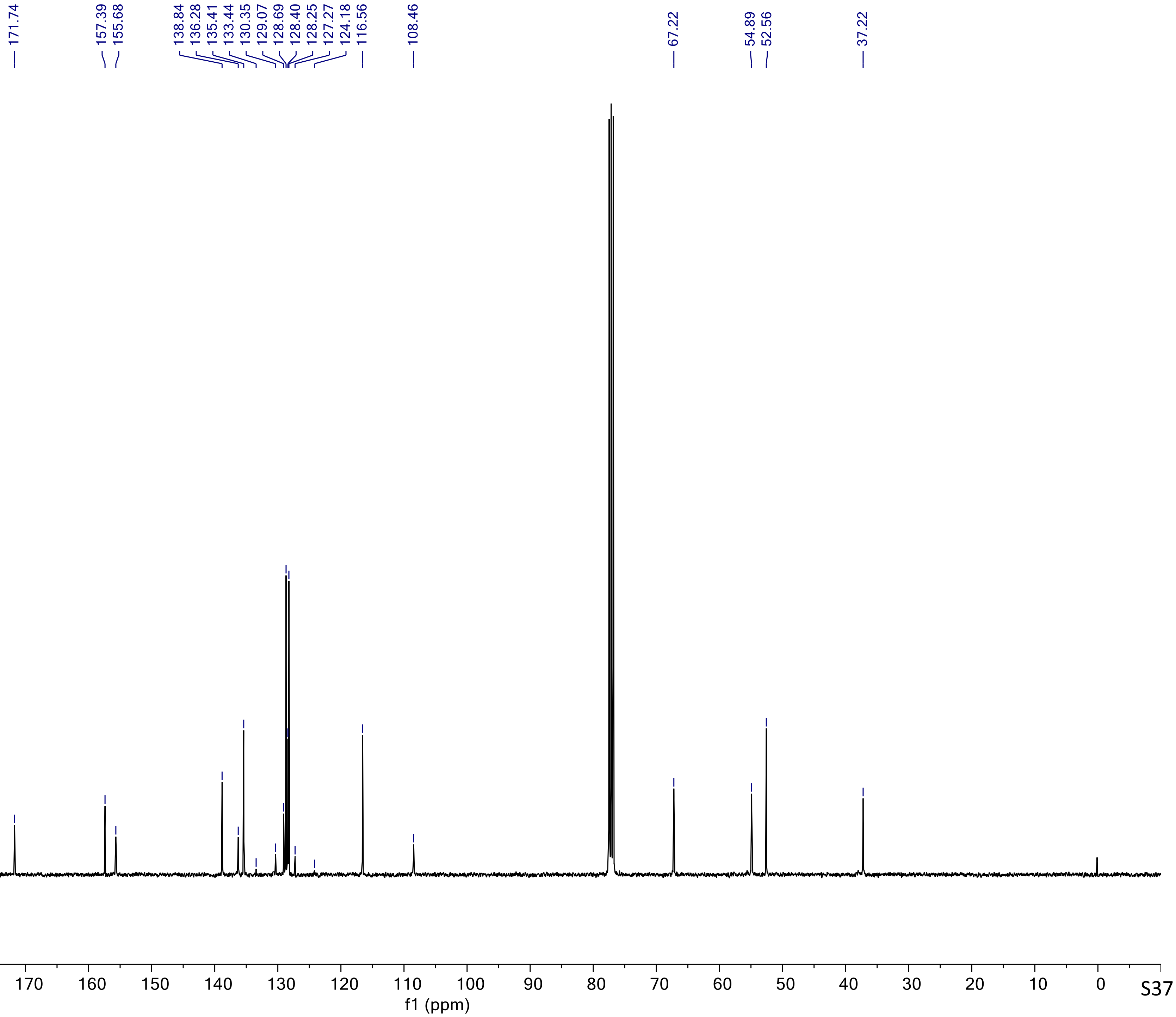

^{19}F NMR (376 MHz, $CDCl_3$)

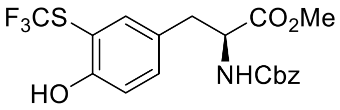

6o

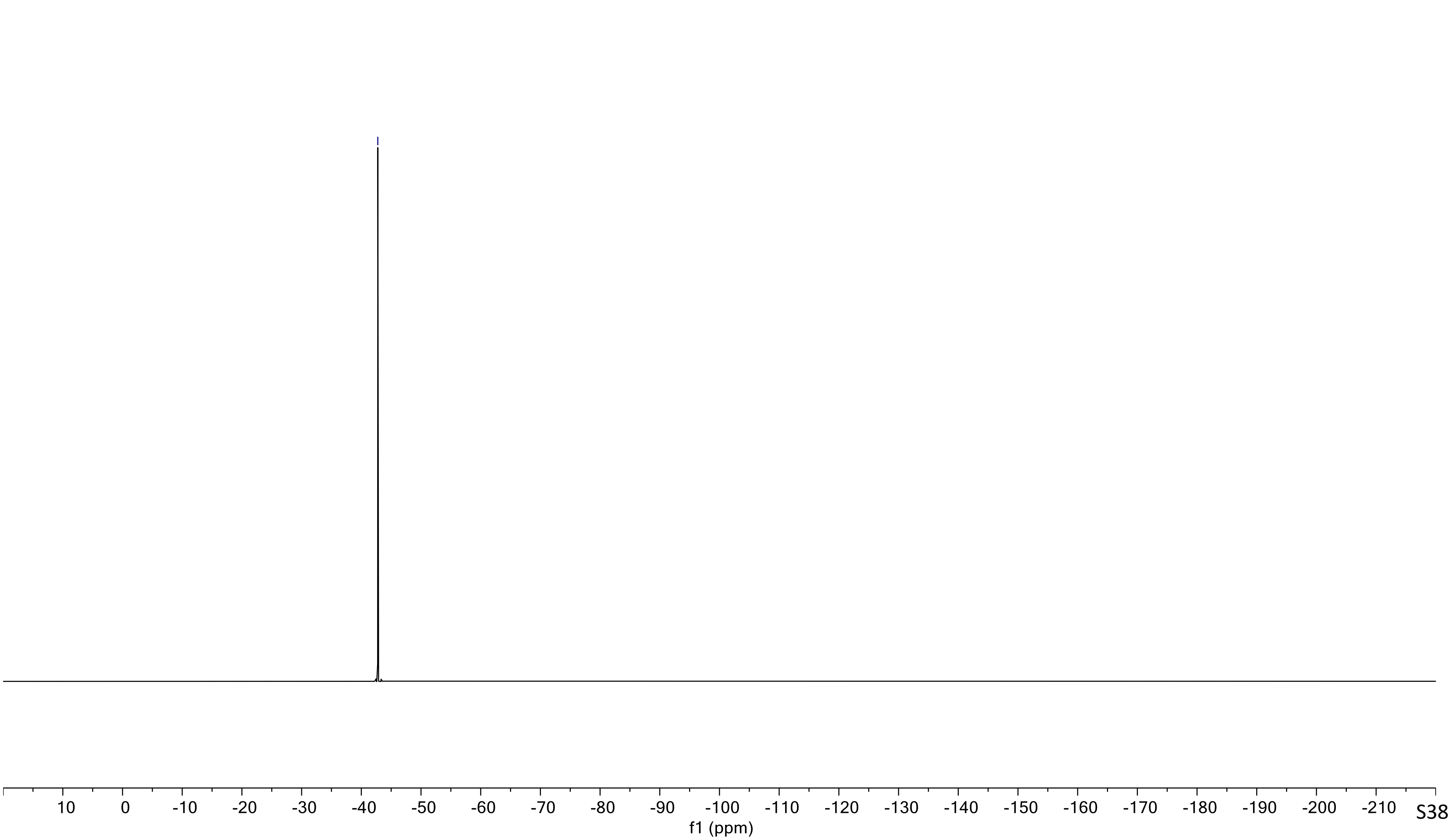

^{1}H NMR (400 MHz, $CDCl_3$)

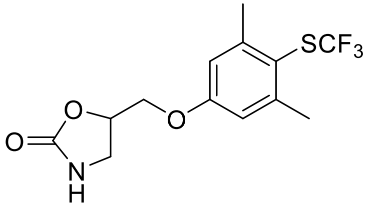

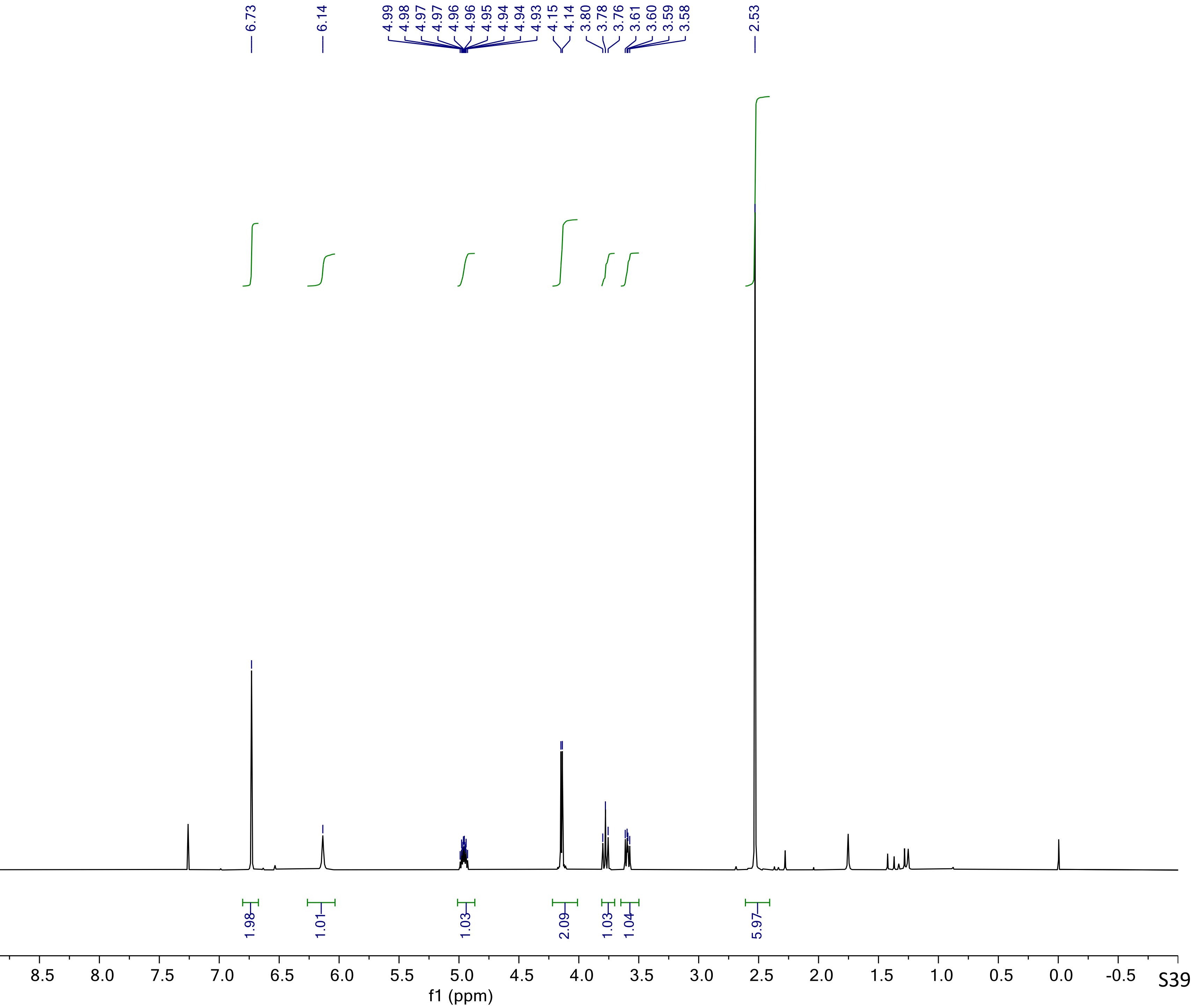

$^{13}C\{^{1}H\}$ NMR (101 MHz, $CDCl_3$)

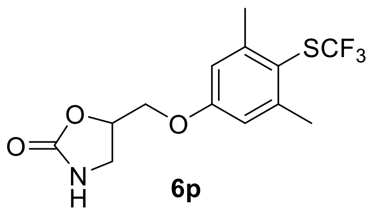

6p

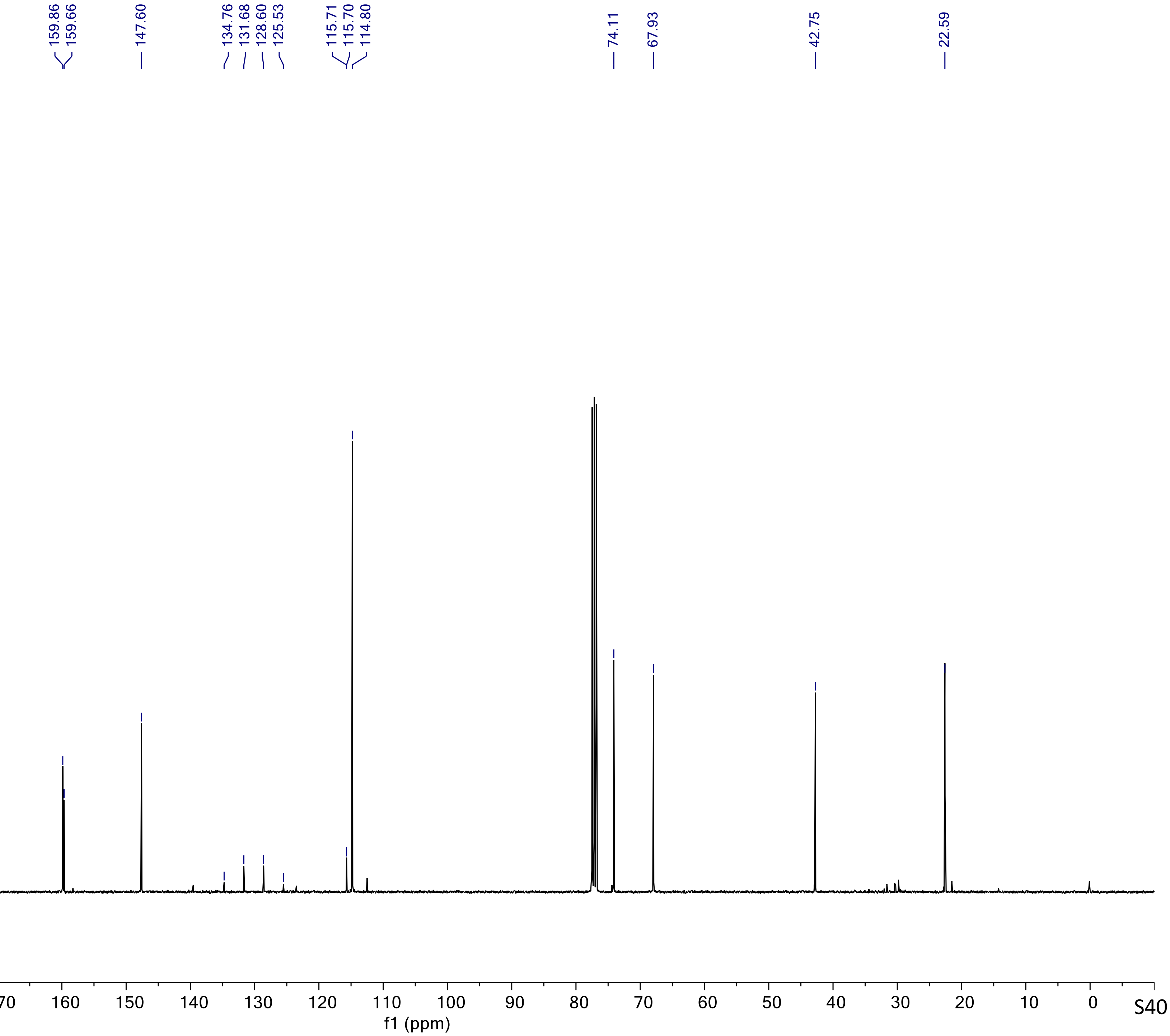

^{19}F NMR (376 MHz, $CDCl_3$)

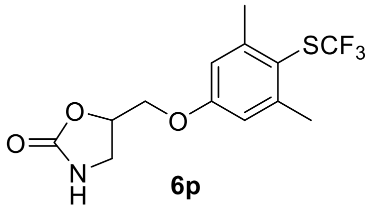

6p

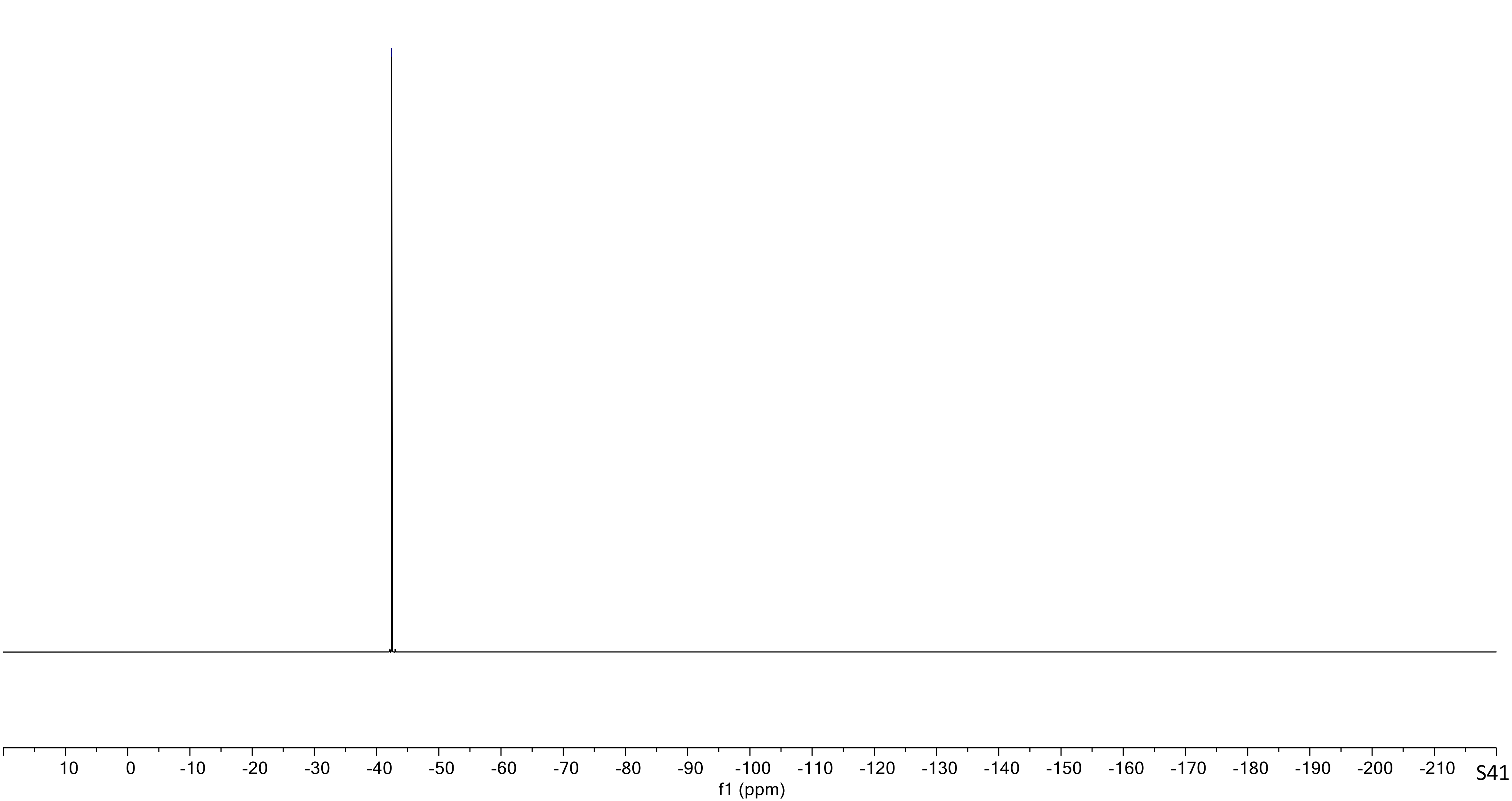

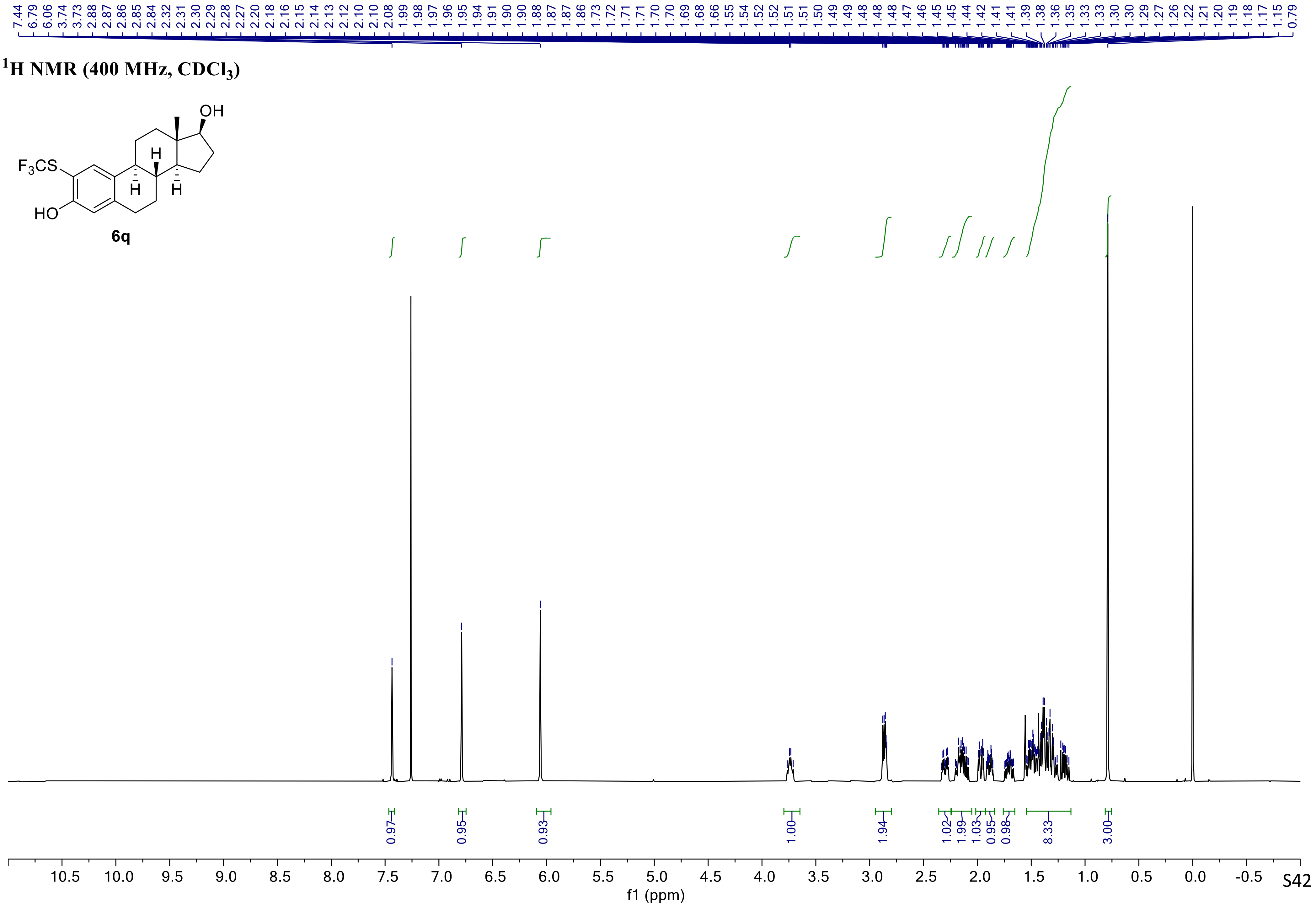

^{1}H NMR (400 MHz, $CDCl_3$)

6q

$^{13}C\{^{1}H\}$ NMR (101 MHz, $CDCl_3$)

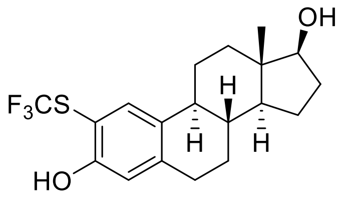

6q

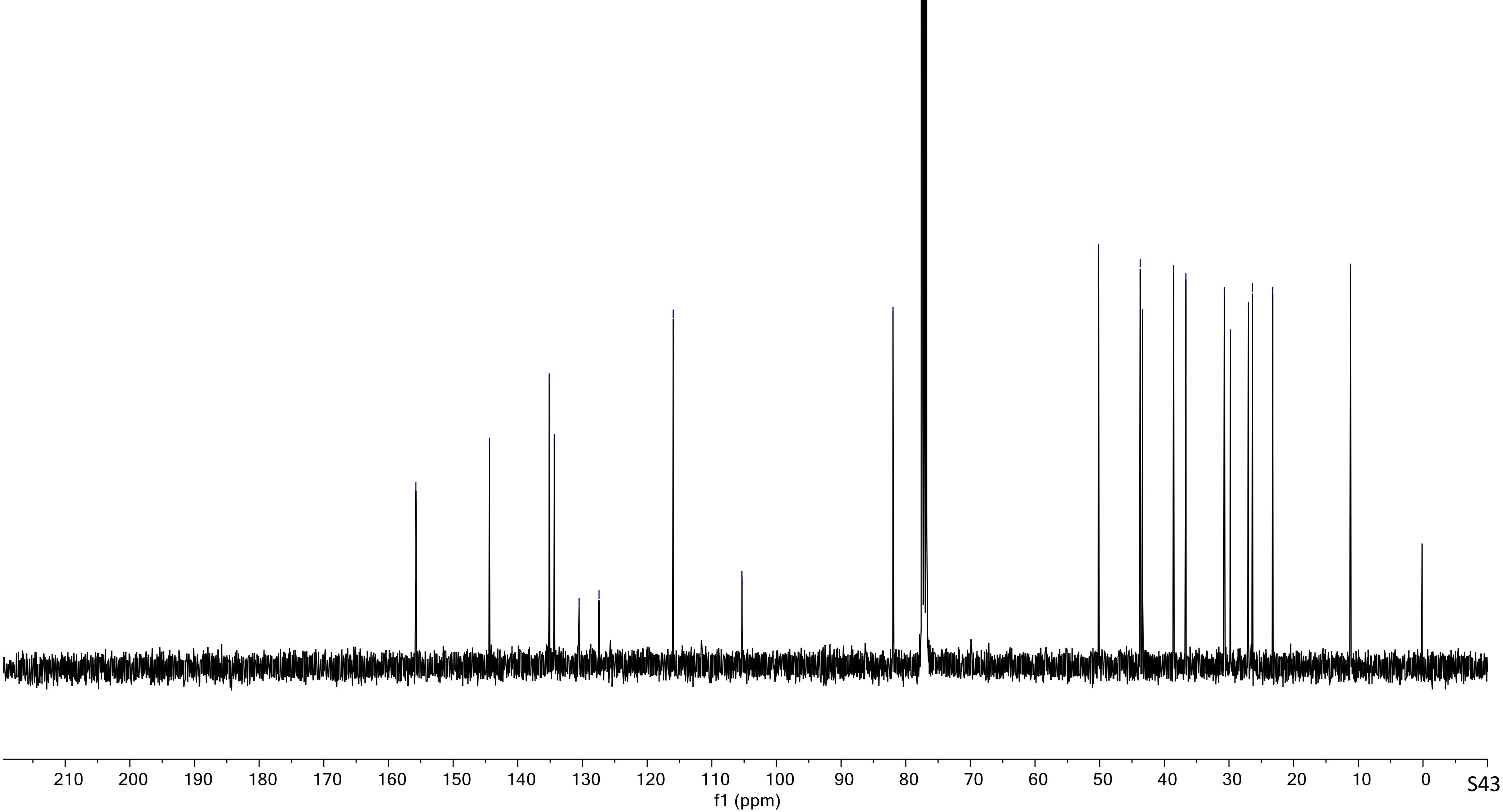

^{19}F NMR (376 MHz, CDCl$_3$)

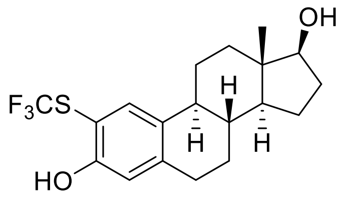

6q

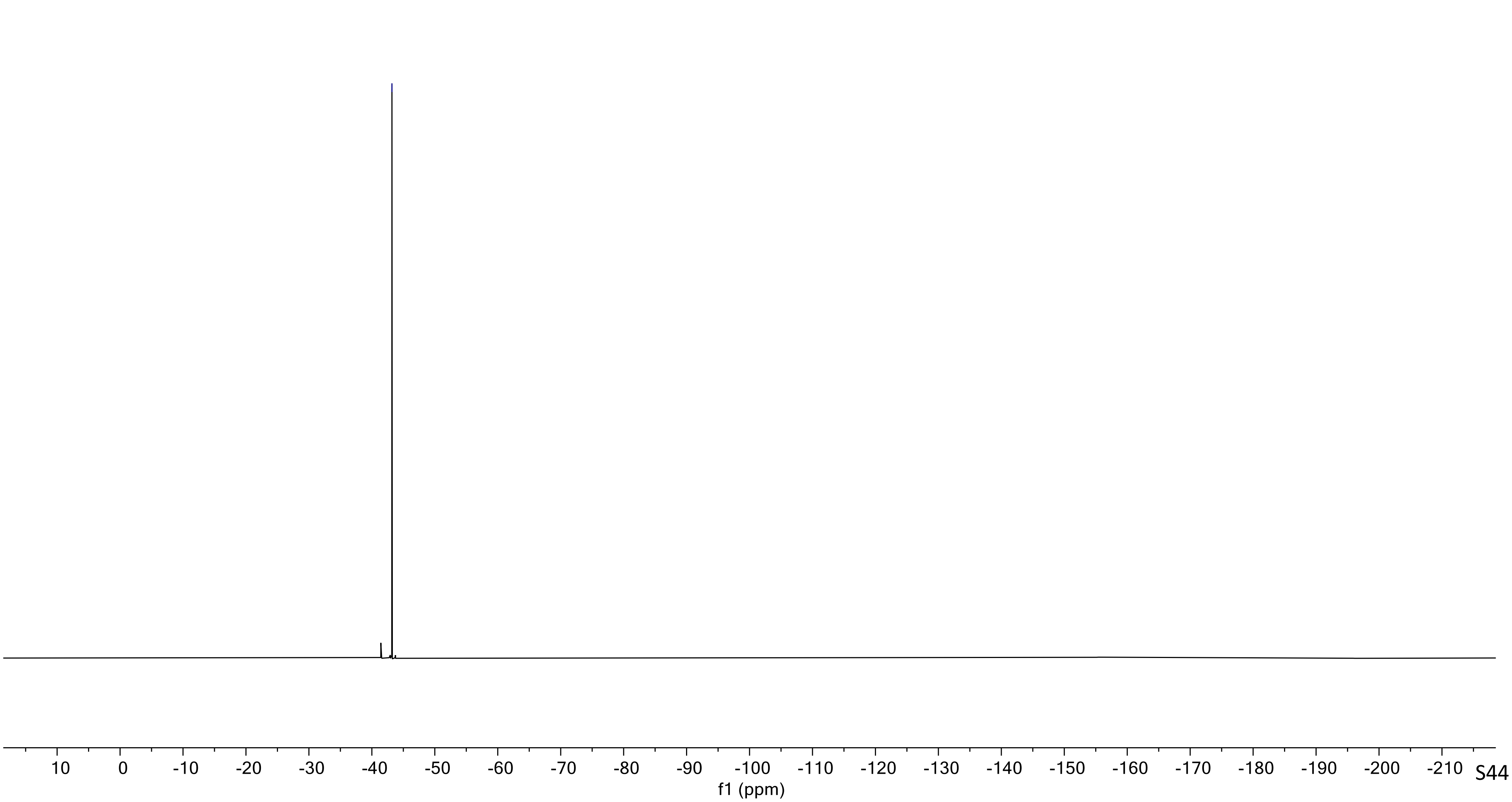

^{1}H NMR (400 MHz, $CDCl_3$)

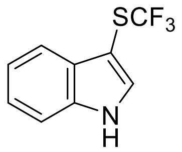

9a

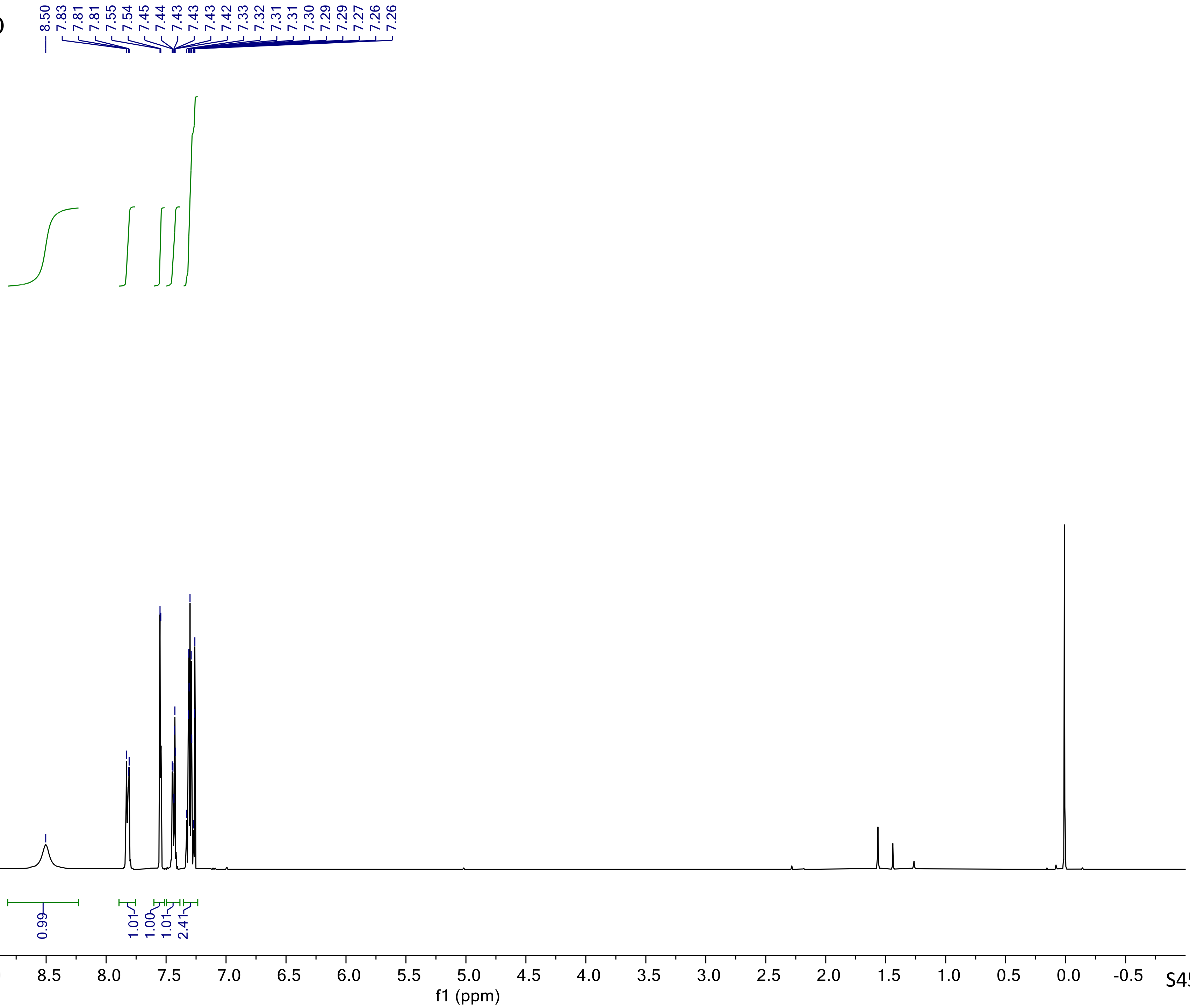

$^{13}C\{^{1}H\}$ NMR (101 MHz, $CDCl_3$)

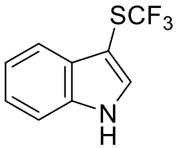

9a

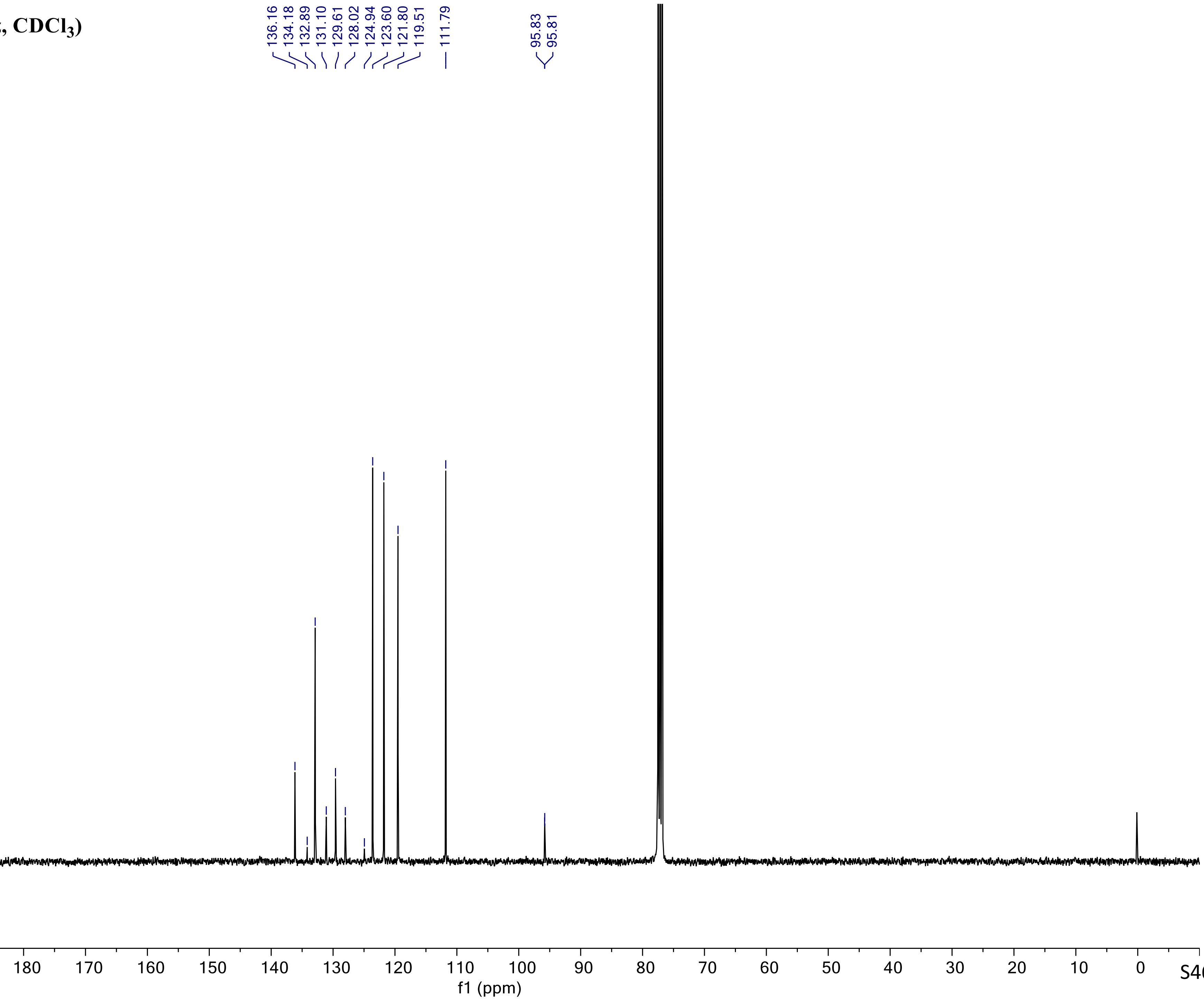

^{1}H NMR (400 MHz, CDCl$_3$)

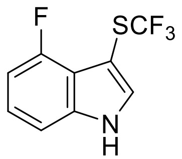

9b

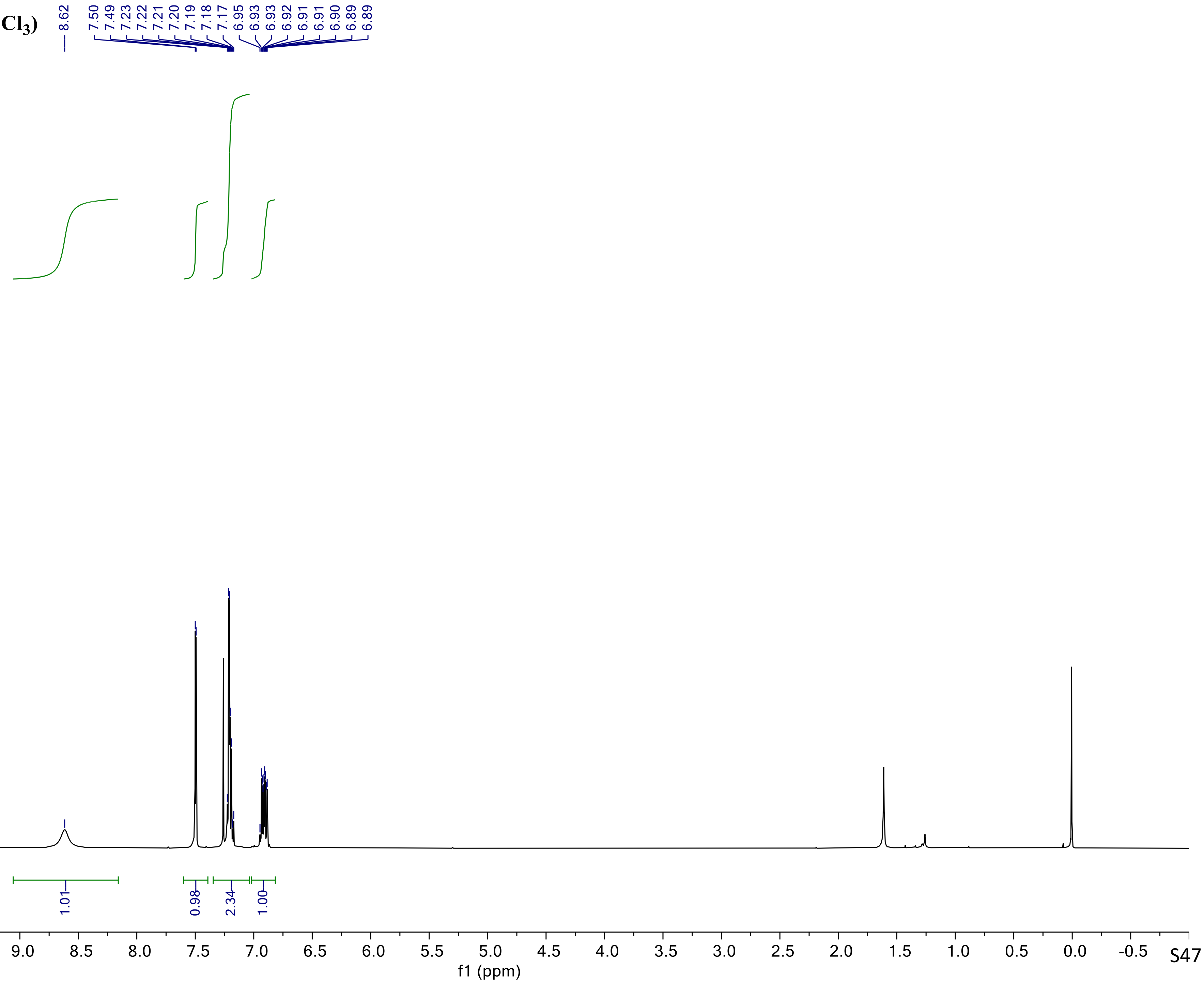

$^{13}C\{^{1}H\}$ NMR (101 MHz, $CDCl_3$)

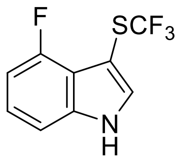

9b

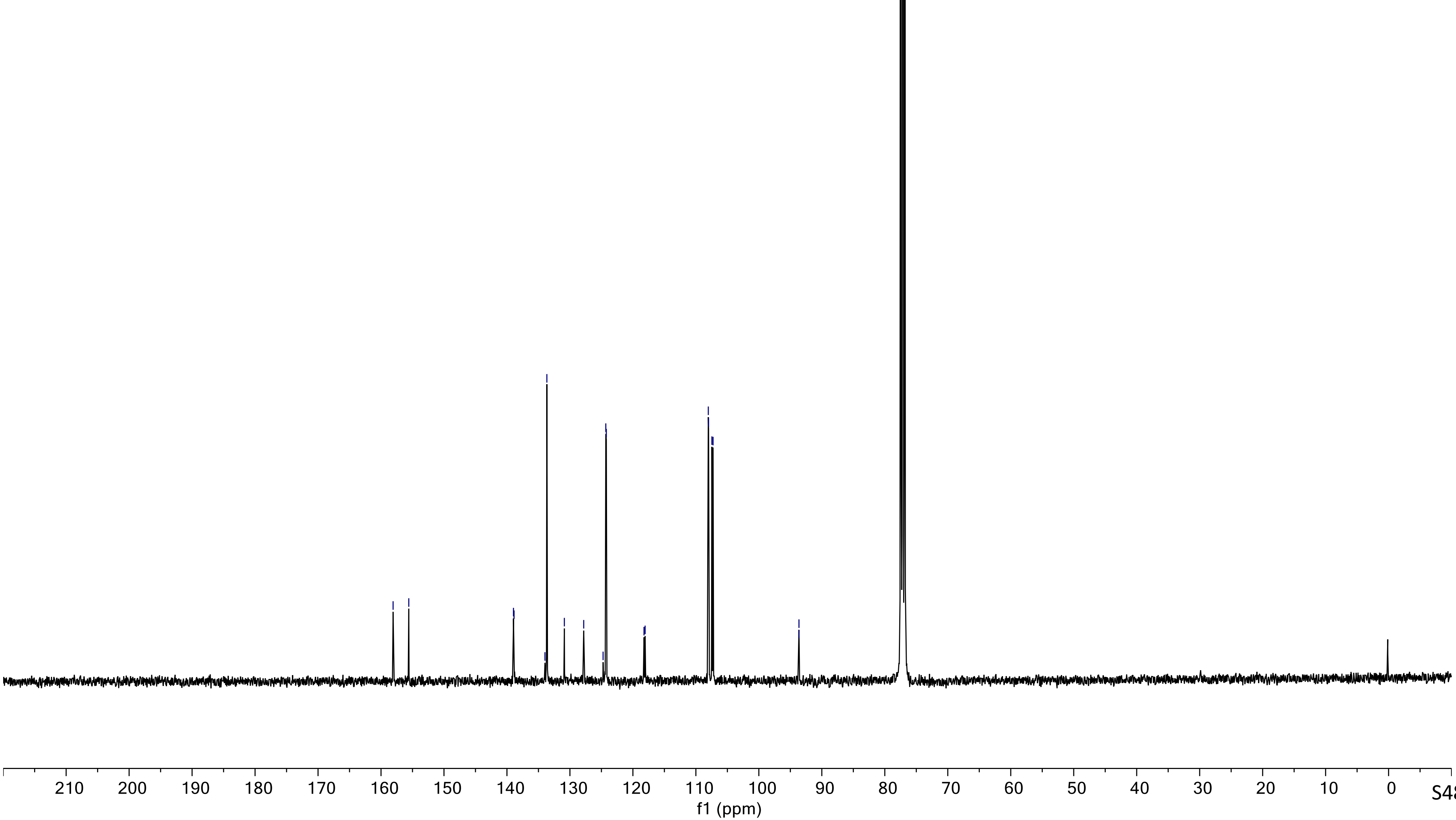

^{19}F NMR (376 MHz, $CDCl_3$)

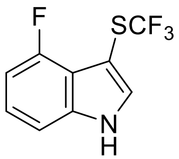

9b

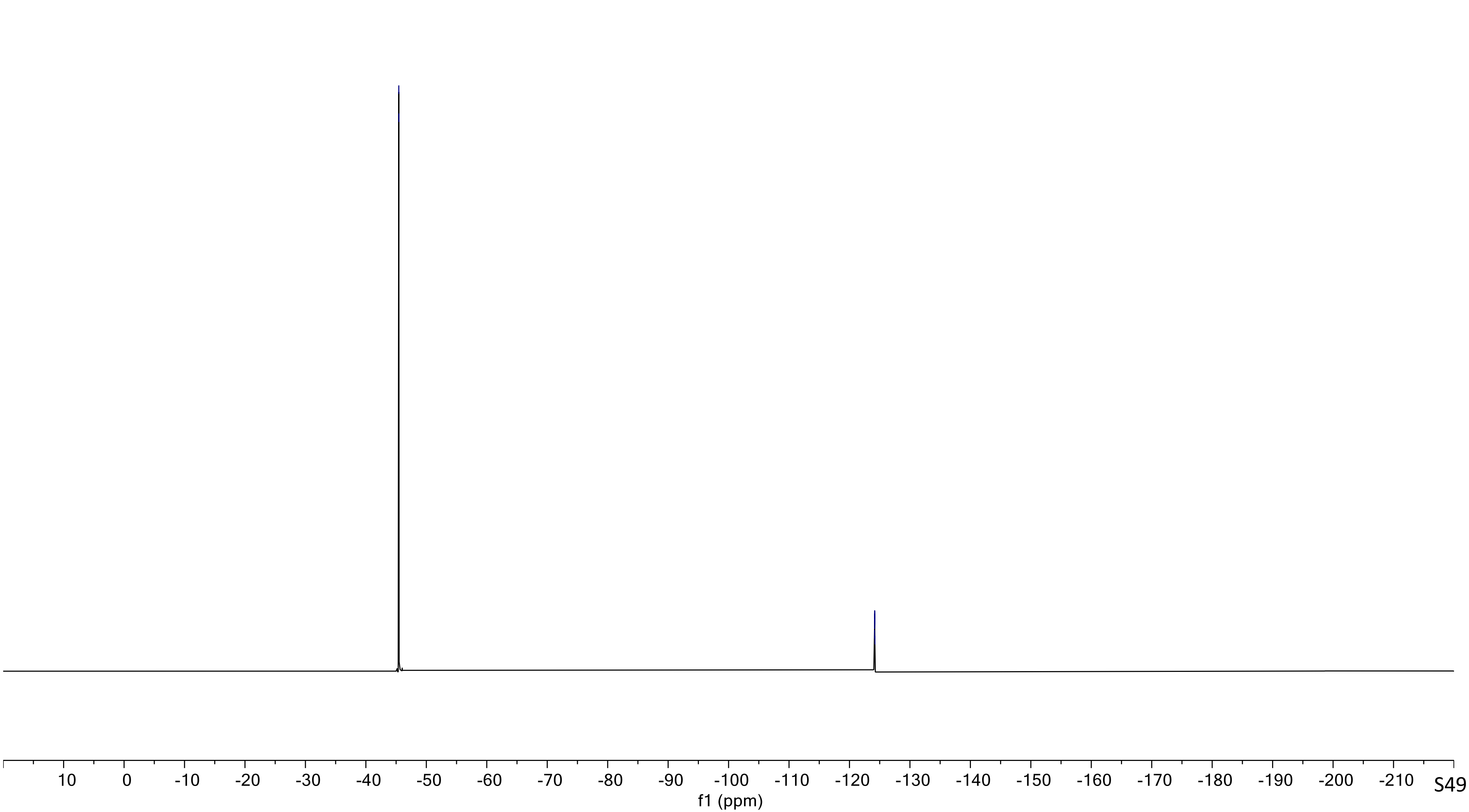

^{1}H NMR (400 MHz, $CDCl_3$)

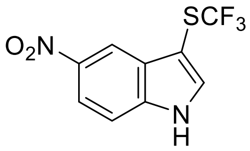

9c

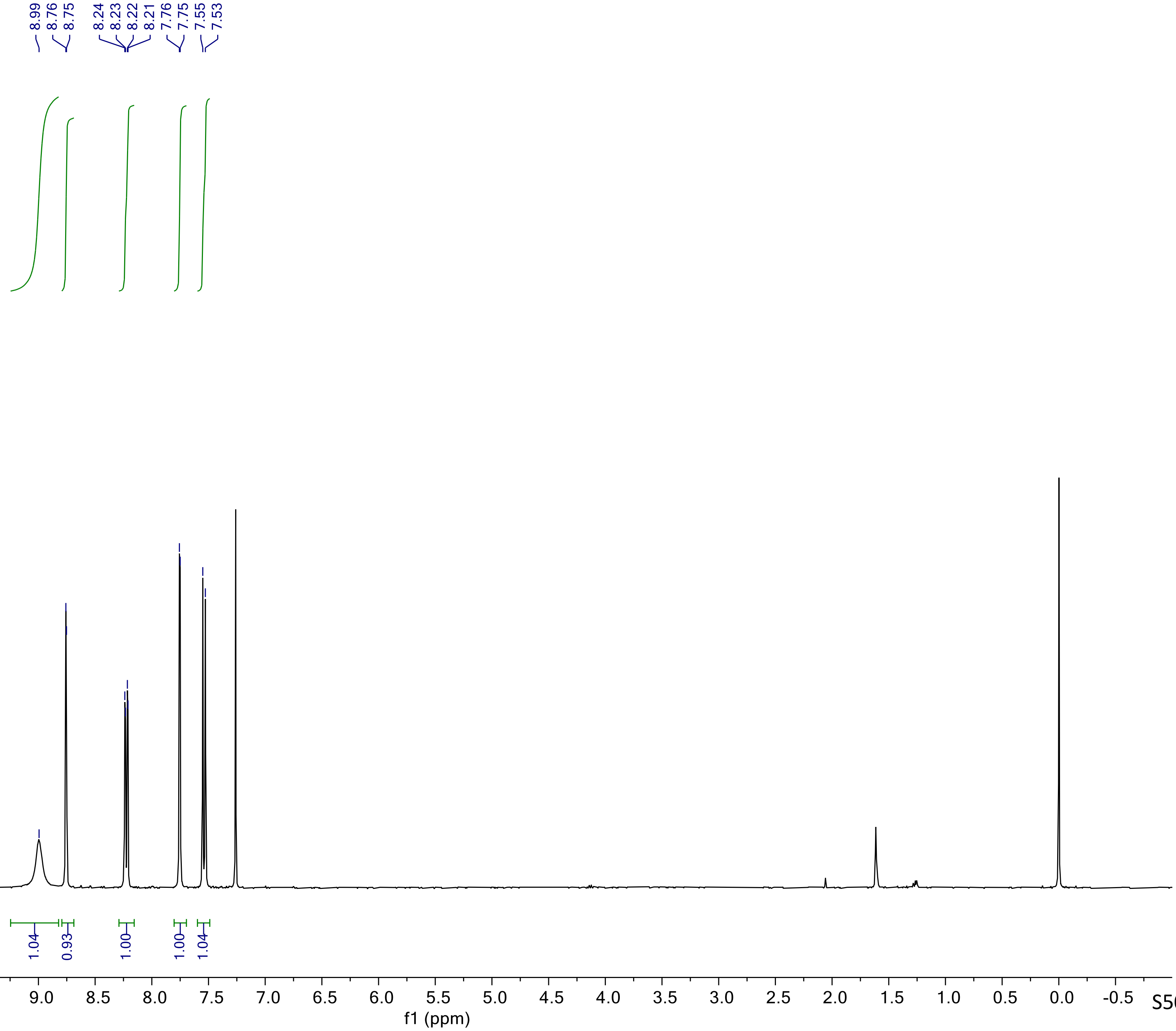

$^{13}C\{^1H\}$ NMR (101 MHz, $CDCl_3$)

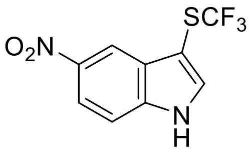

9c

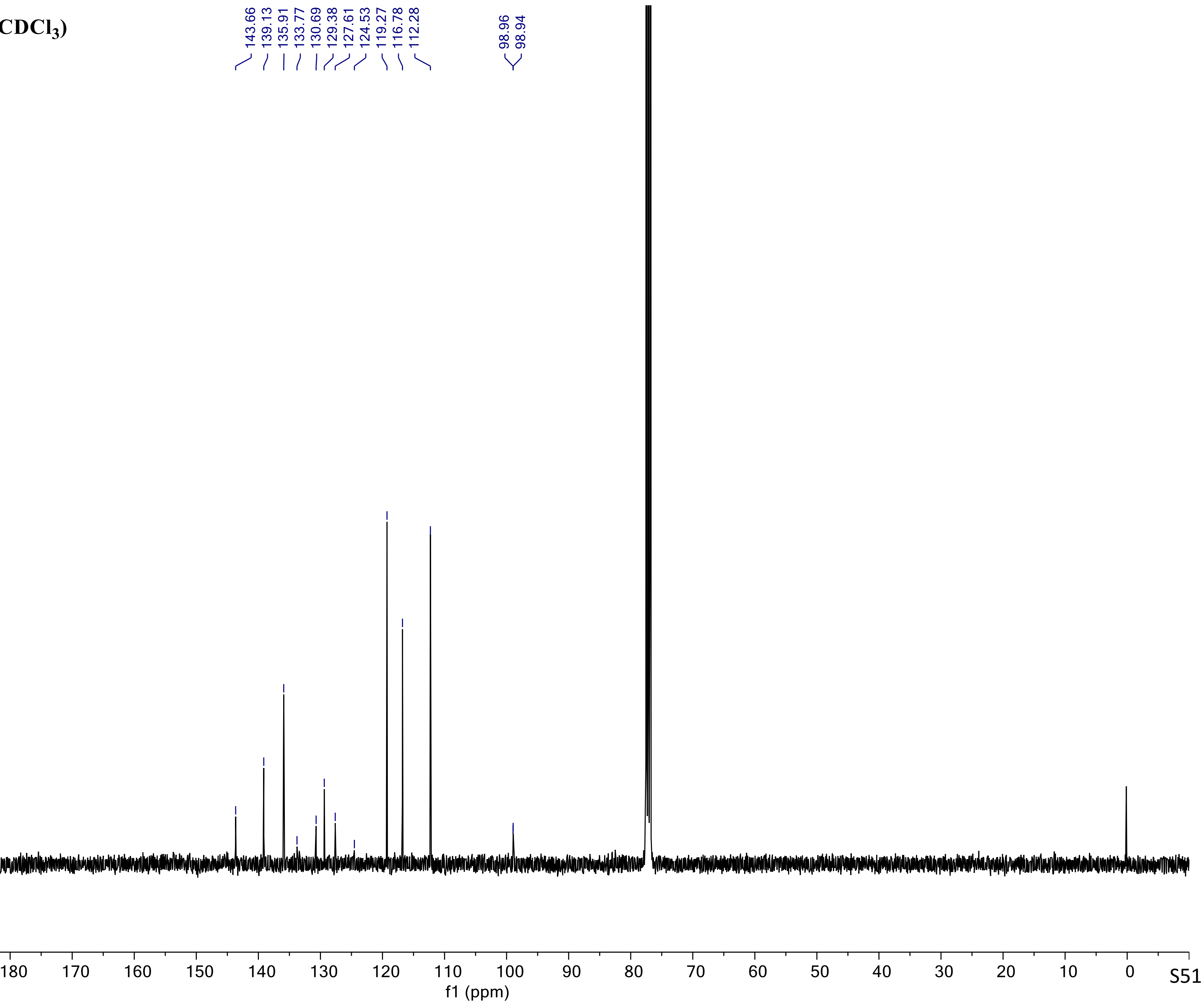

^{1}H NMR (400 MHz, $CDCl_3$)

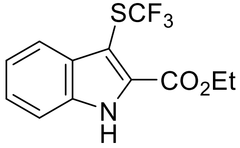

9d

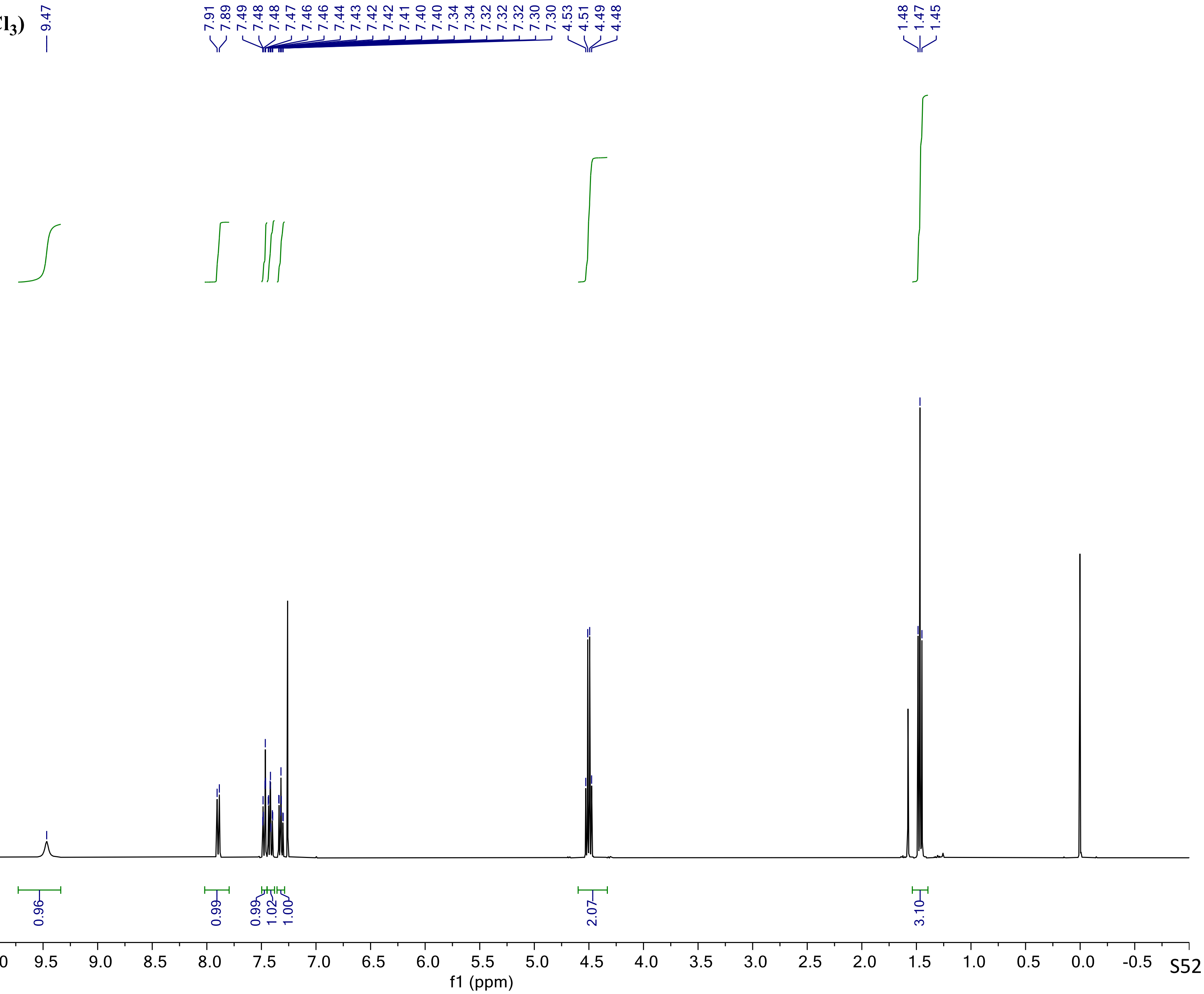

$^{13}C\{^{1}H\}$ NMR (101 MHz, $CDCl_3$)

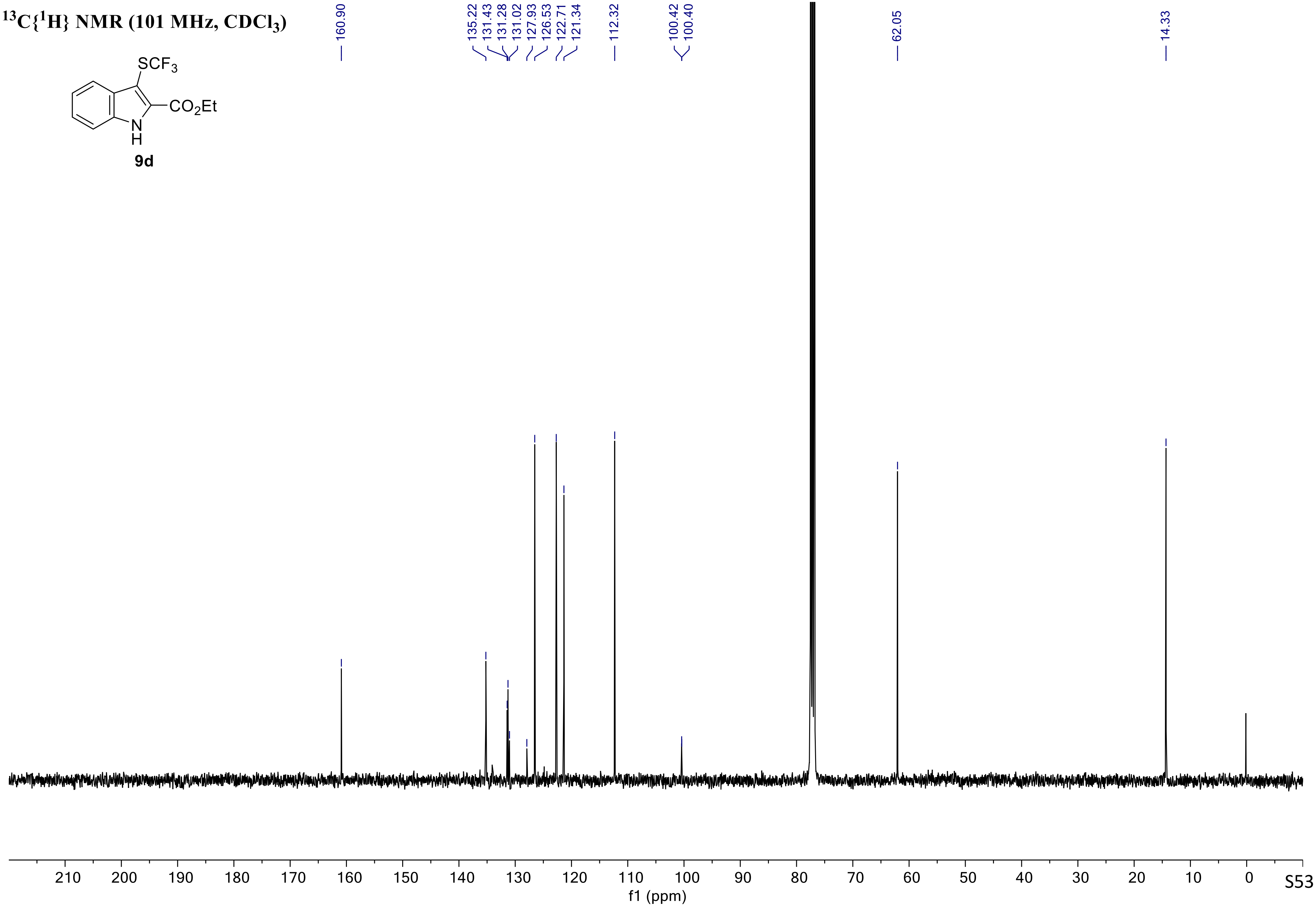

9d

^{19}F NMR (376 MHz, $CDCl_3$)

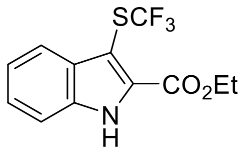

9d

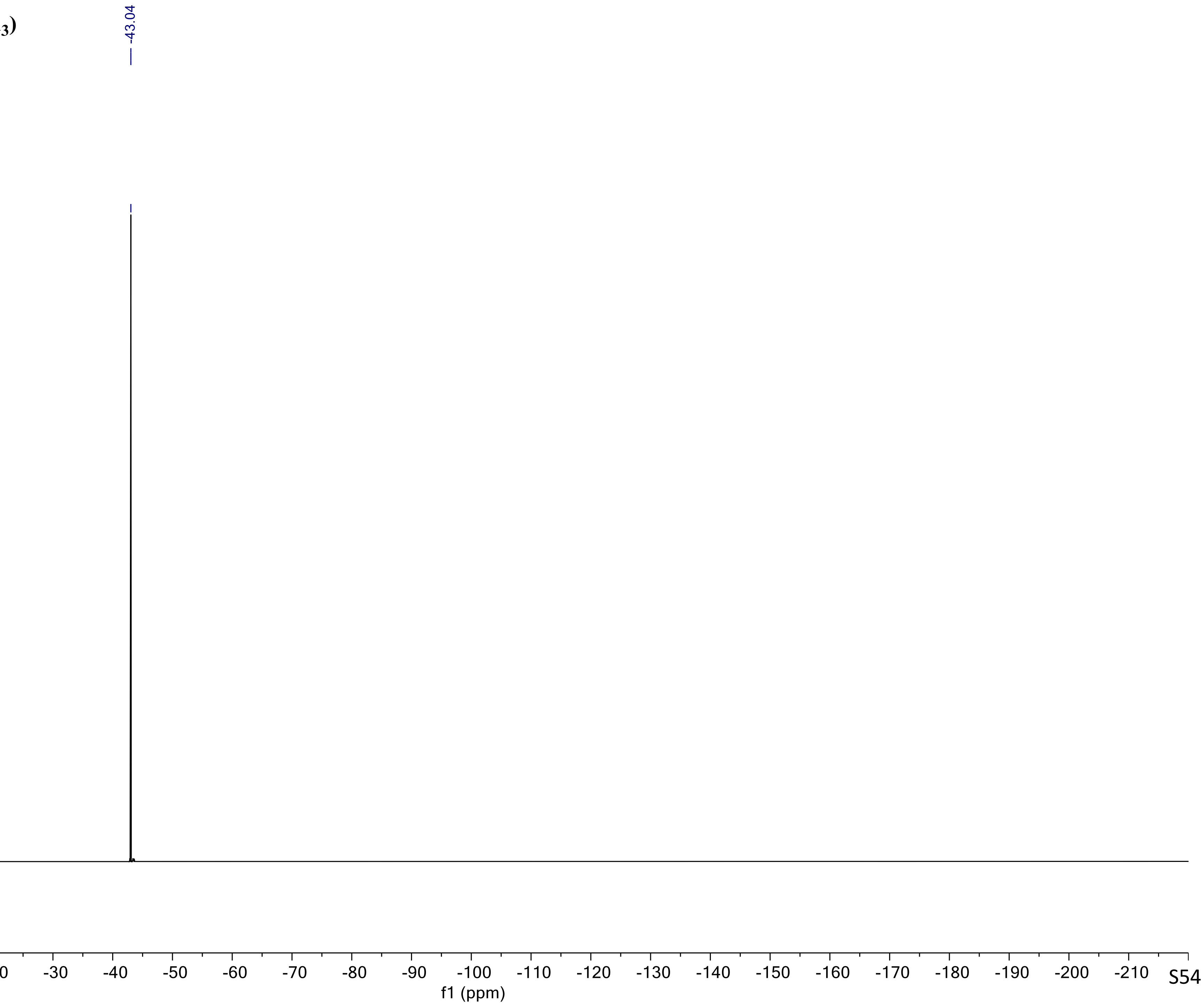

^{1}H NMR (400 MHz, $CDCl_3$)

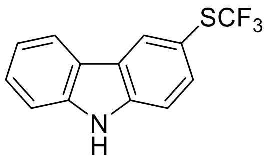

10a

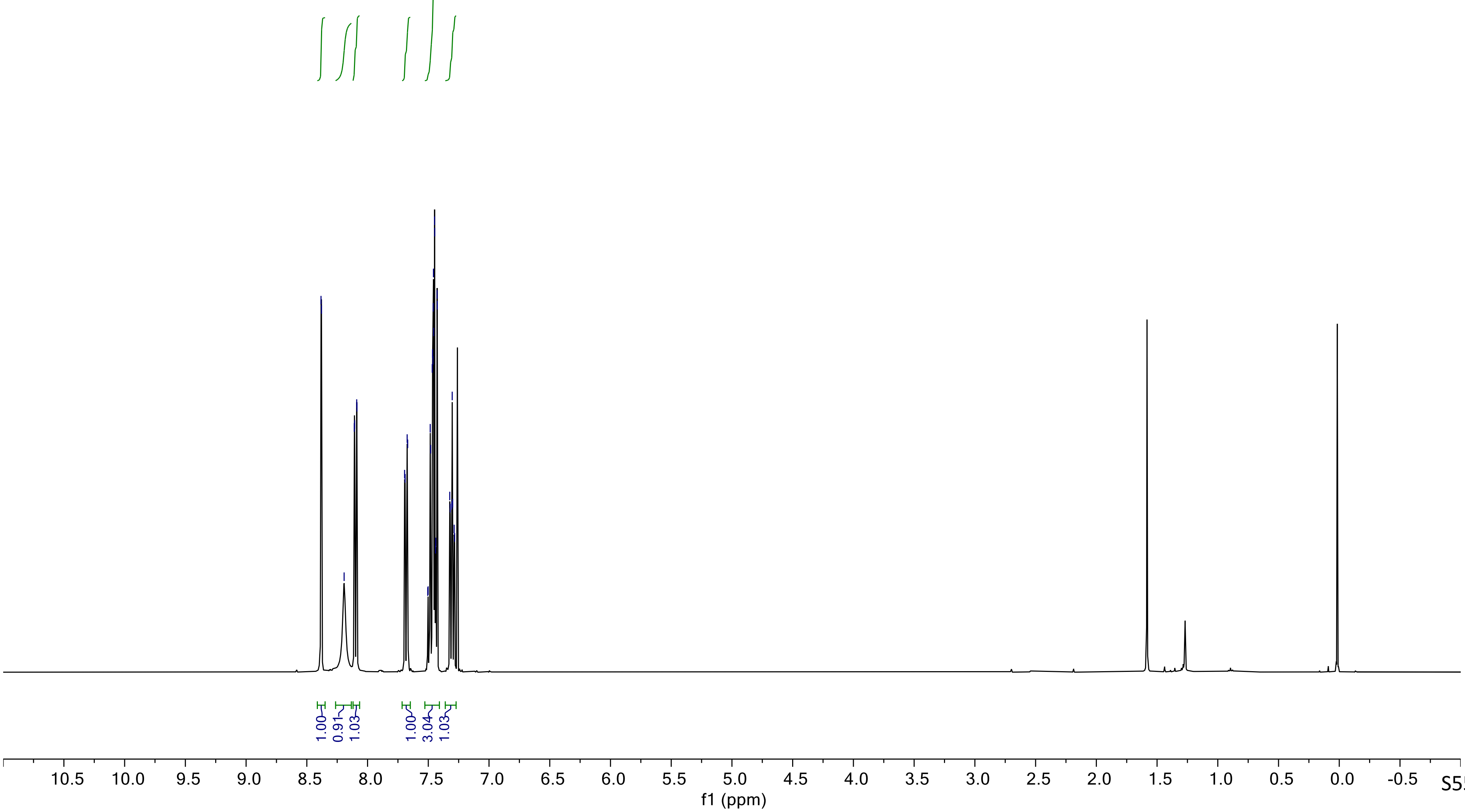

$^{13}C\{^1H\}$ NMR (101 MHz, $CDCl_3$)

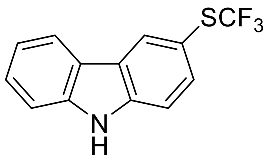

10a

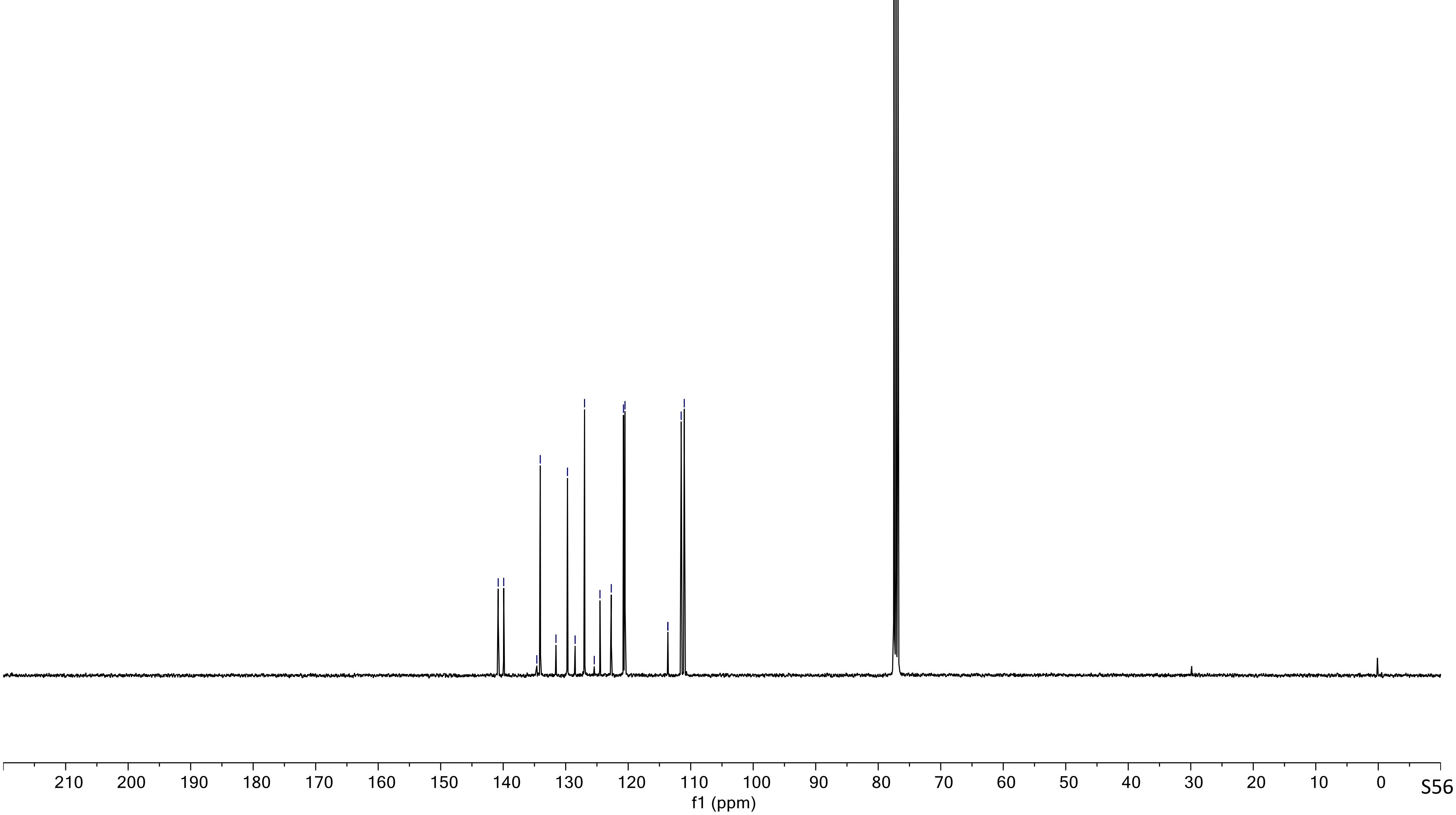

^{1}H NMR (400 MHz, $CDCl_3$)

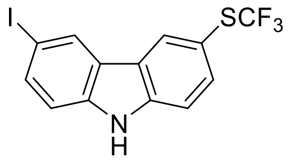

10b

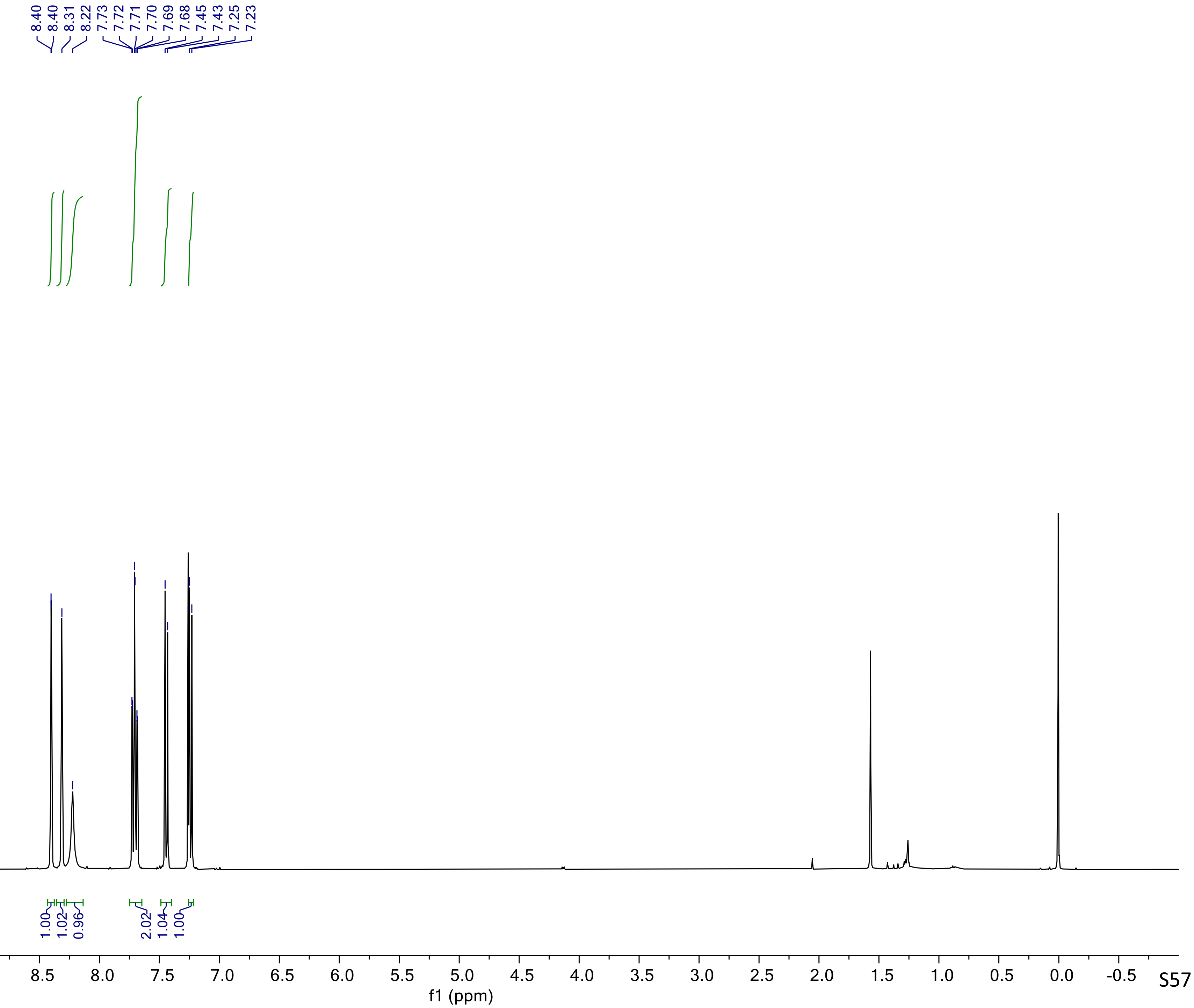

$^{13}C\{^{1}H\}$ NMR (101 MHz, $CDCl_3$)

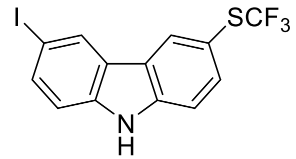

10b

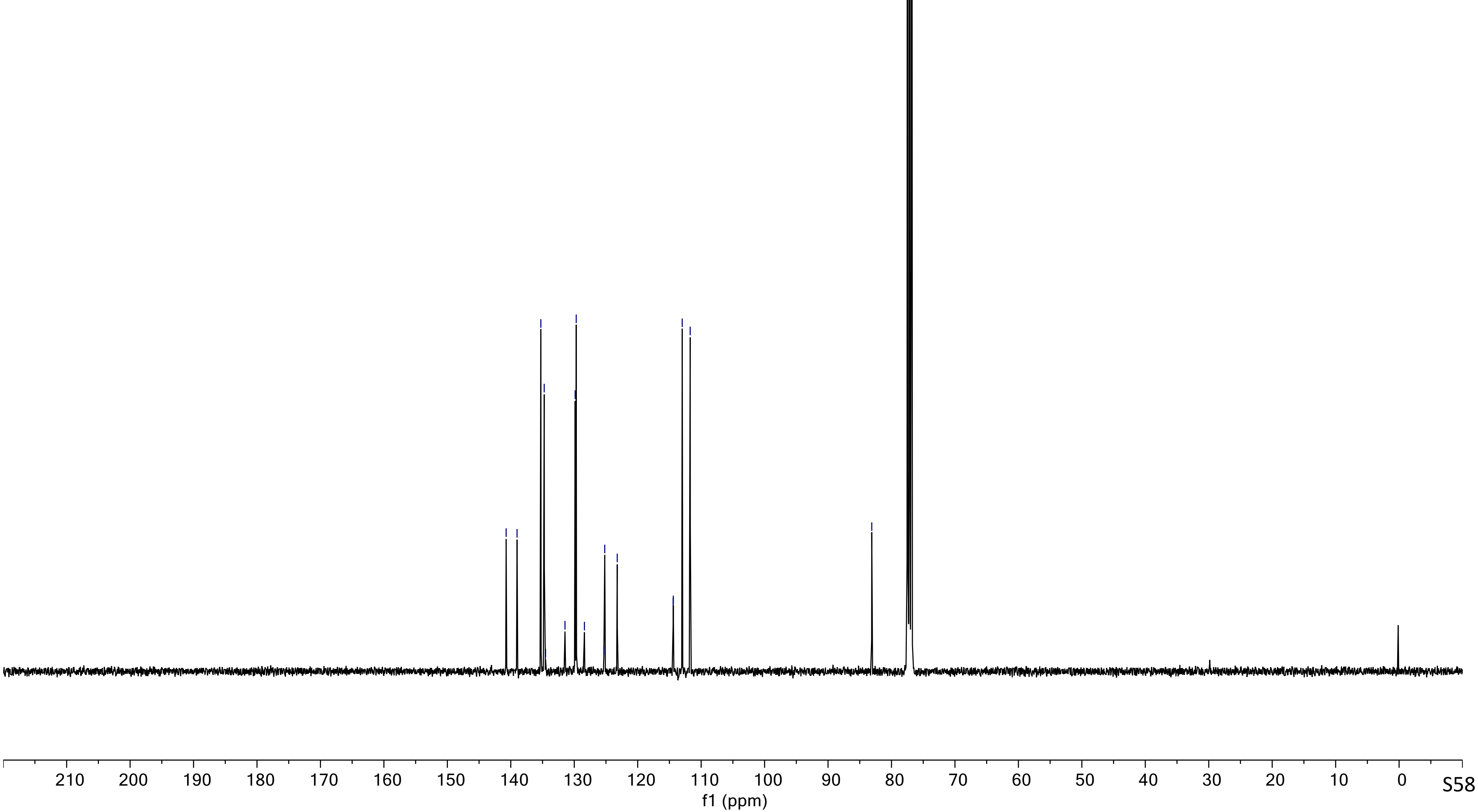

^{19}F NMR (376 MHz, CDCl$_3$)

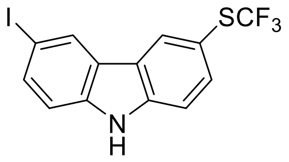

10b

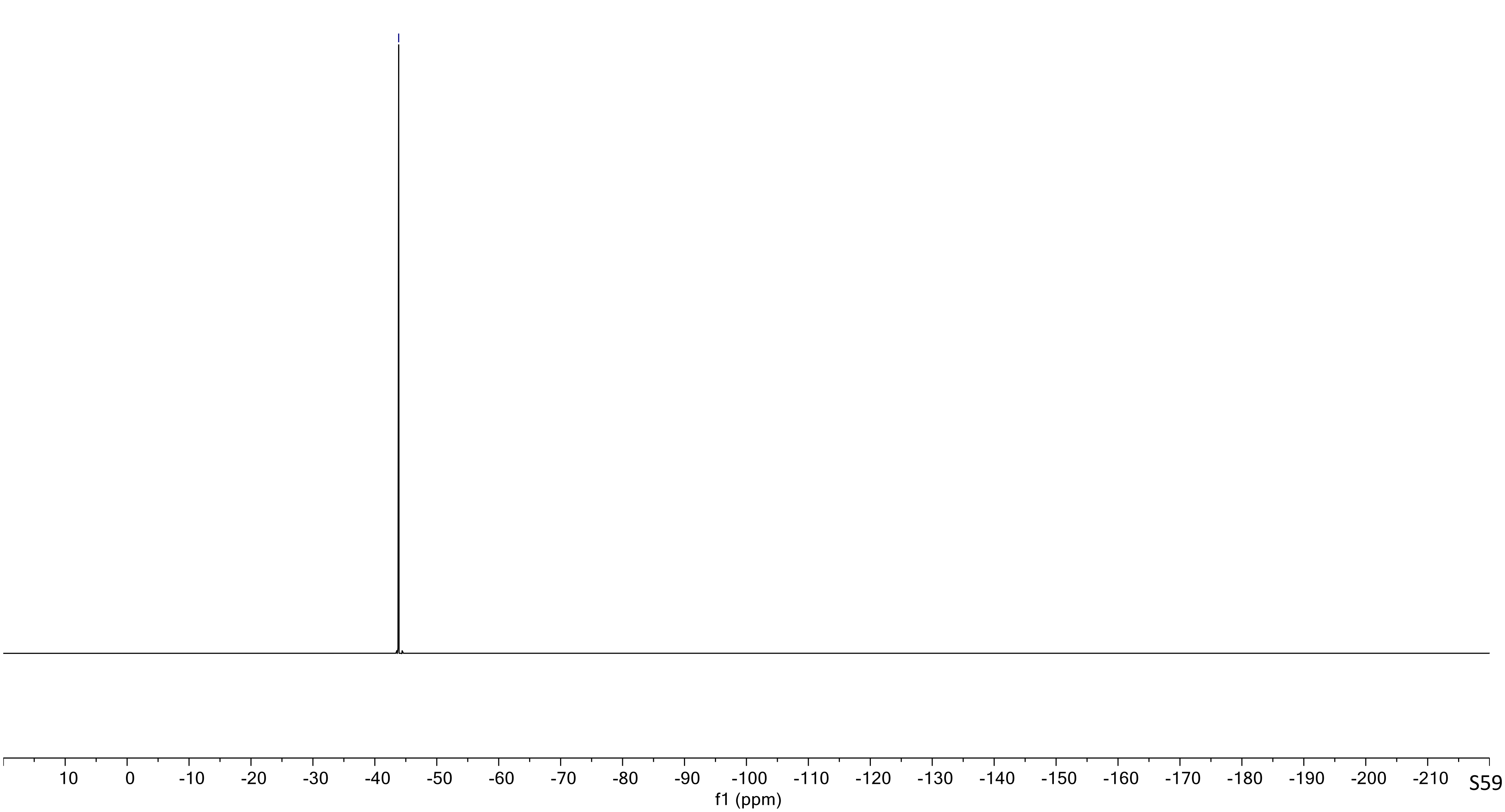

^{1}H NMR (400 MHz, $CDCl_3$)

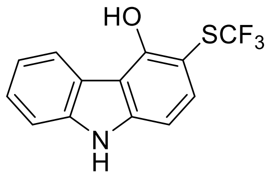

10c

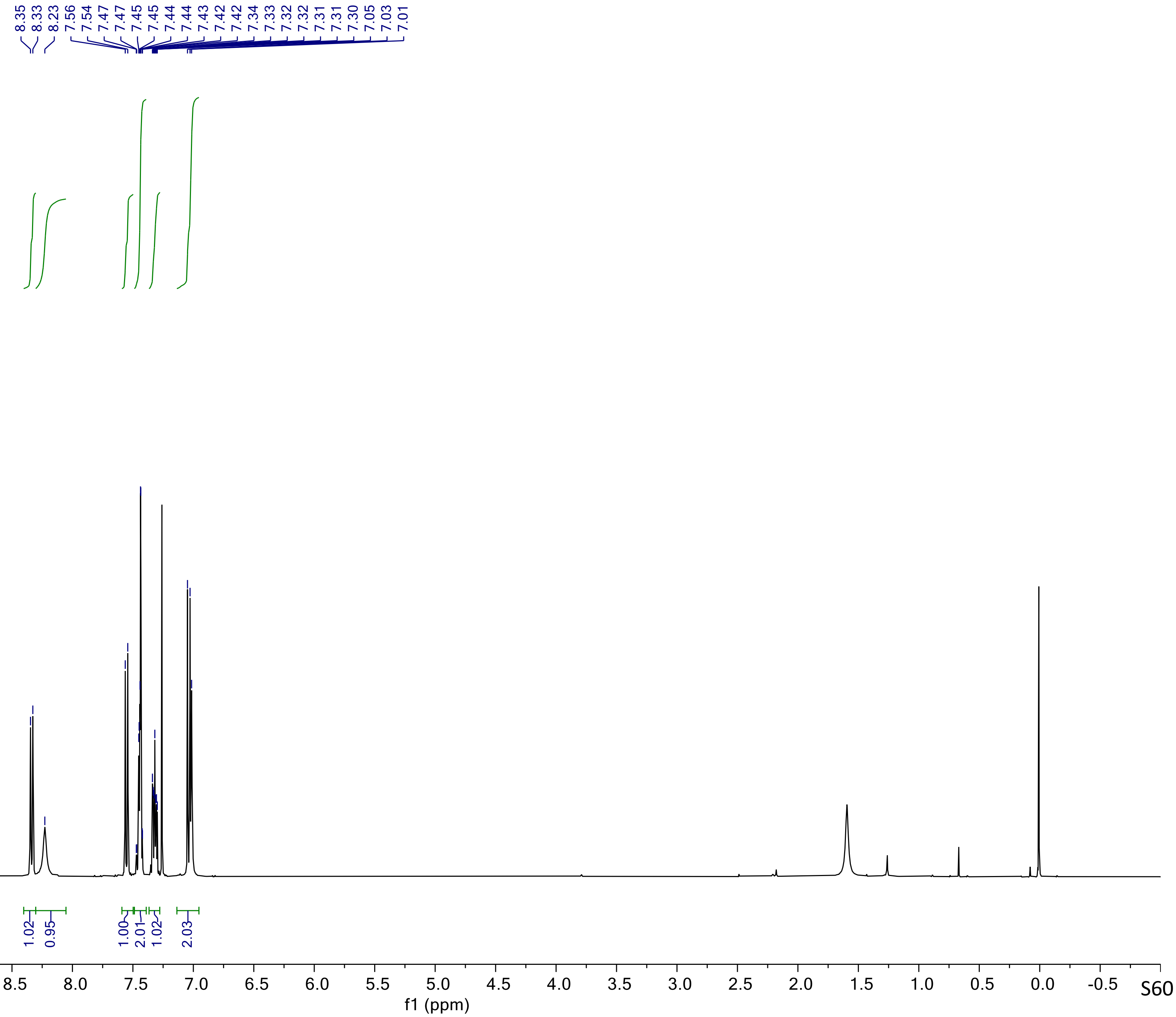

$^{13}C\{^{1}H\}$ NMR (101 MHz, $CDCl_3$)

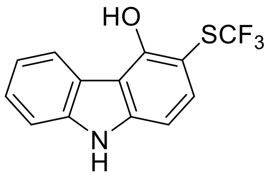

10c

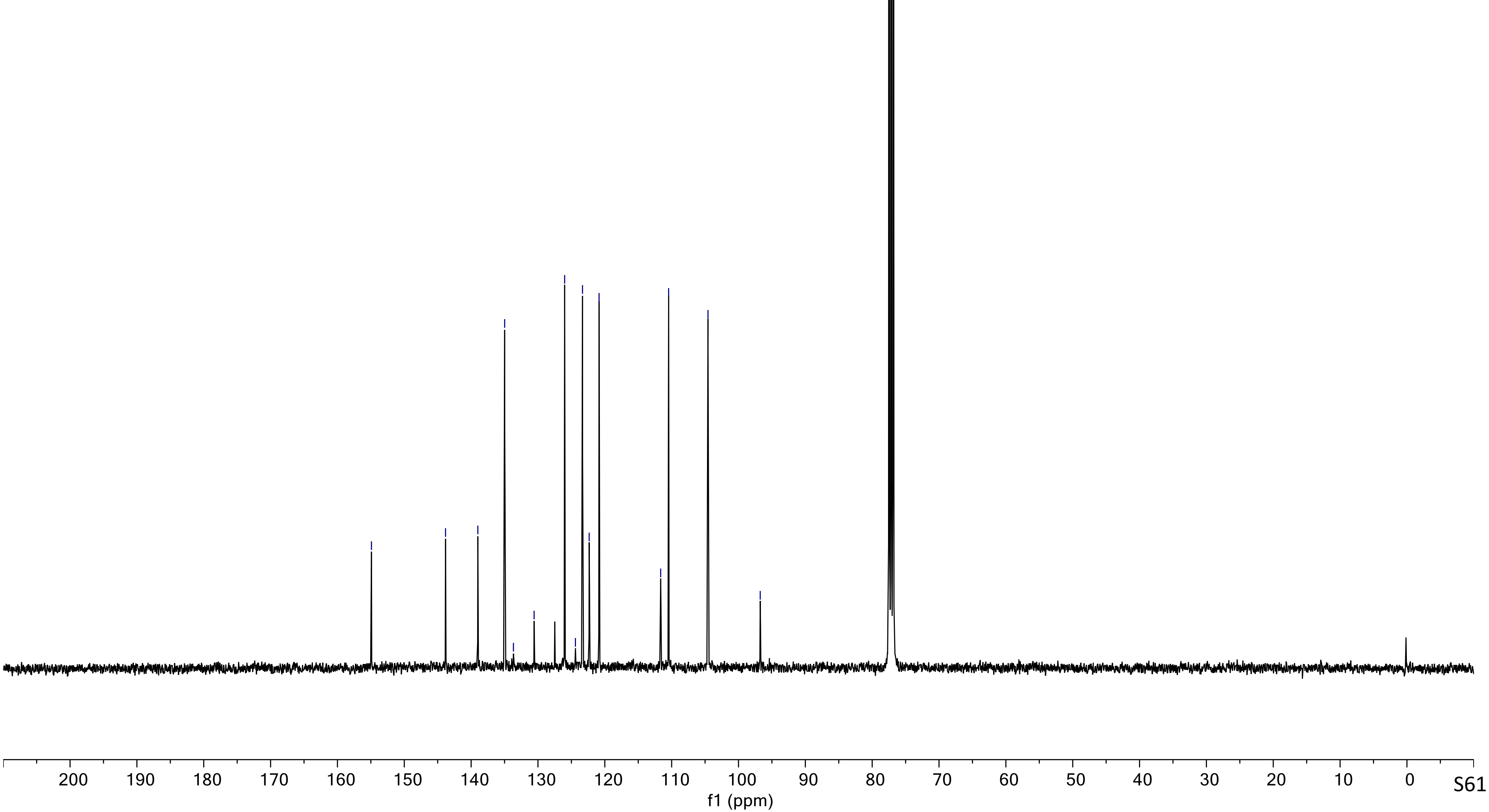

^{19}F NMR (376 MHz, CDCl$_3$)

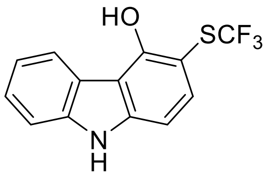

10c

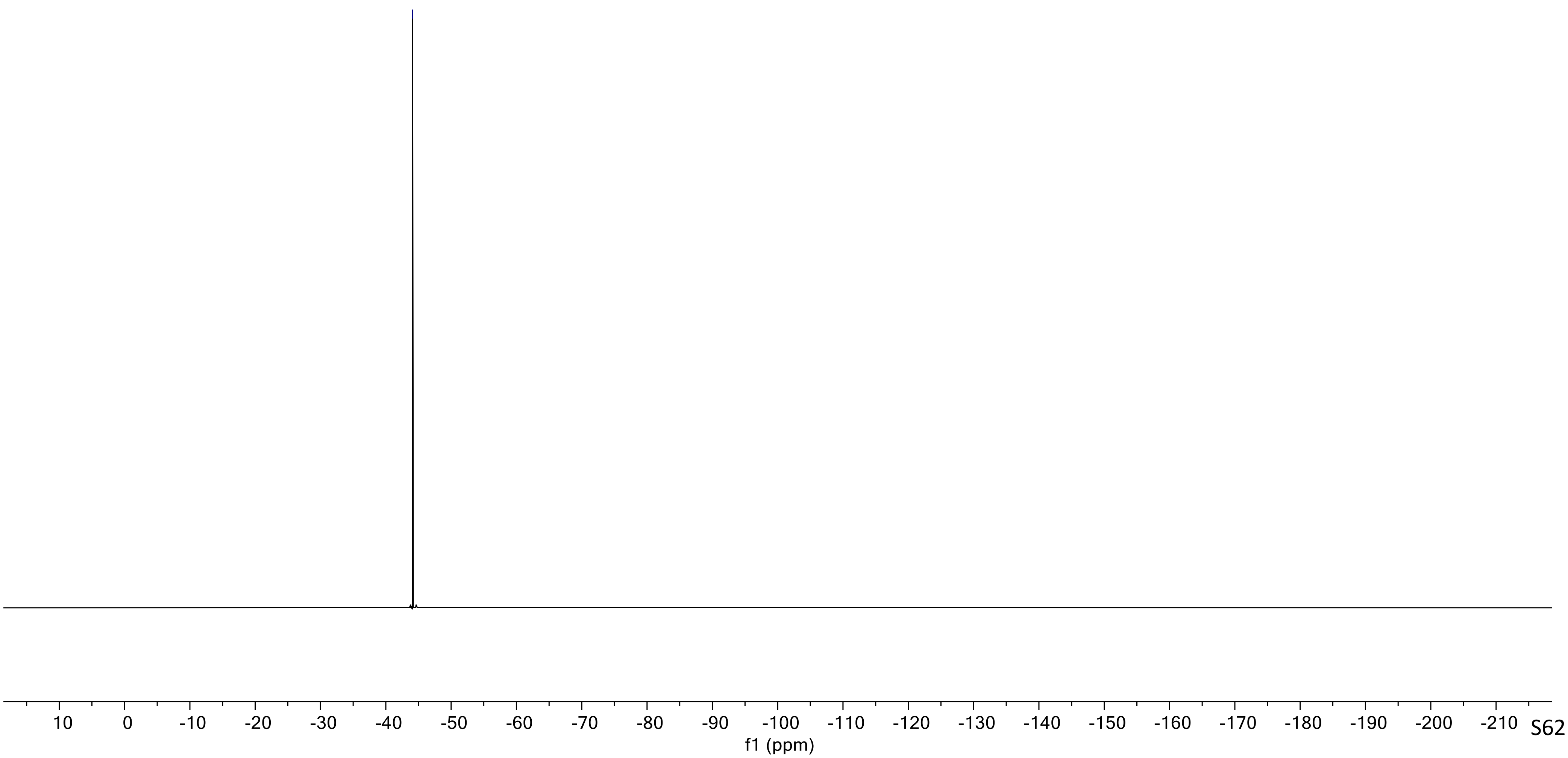

^{1}H NMR (400 MHz, $CDCl_3$)

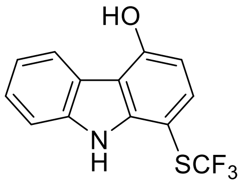

10d

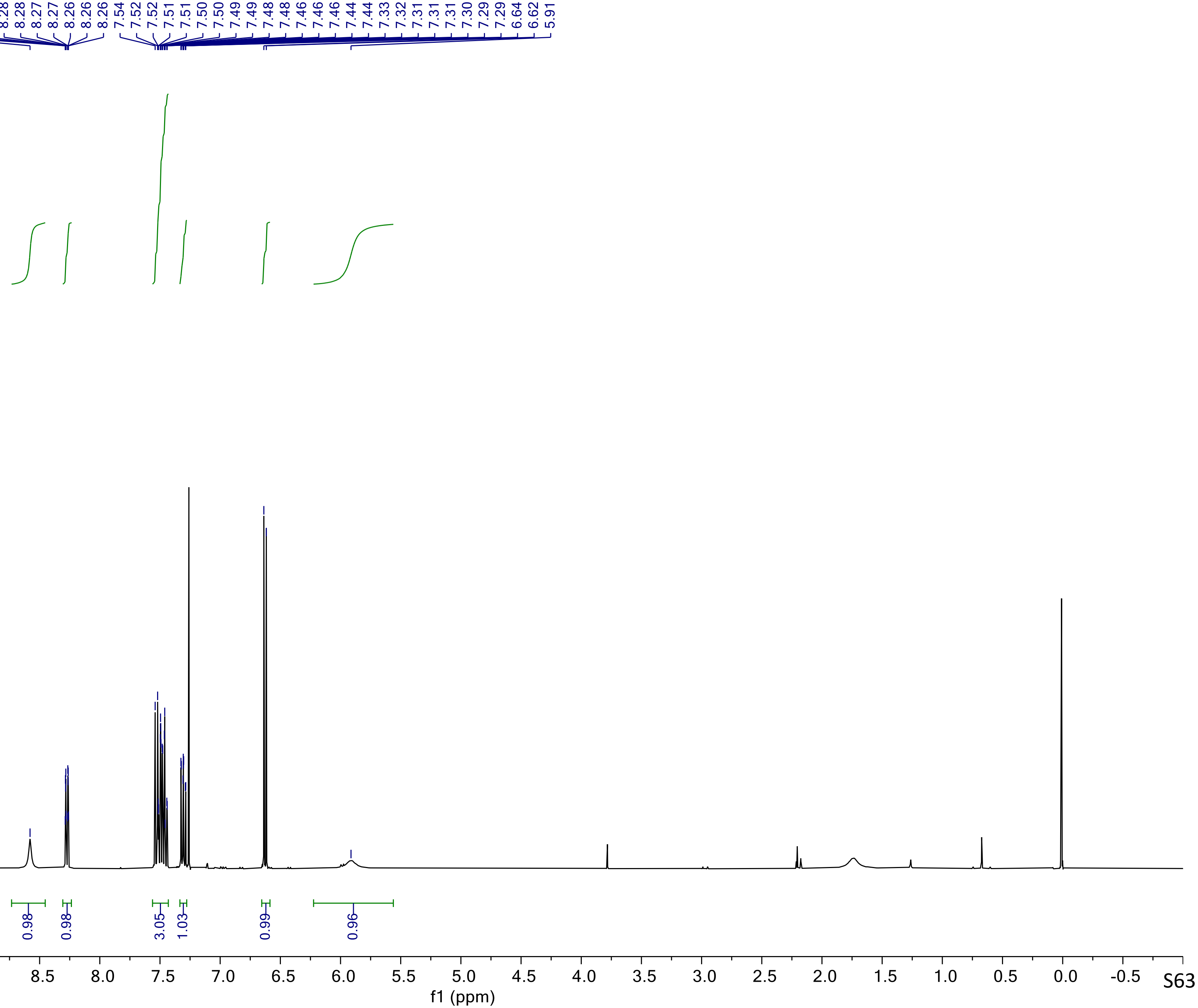

$^{13}C\{^{1}H\}$ NMR (101 MHz, $CDCl_3$)

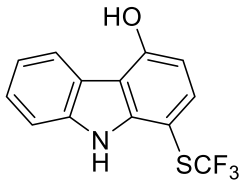

10d

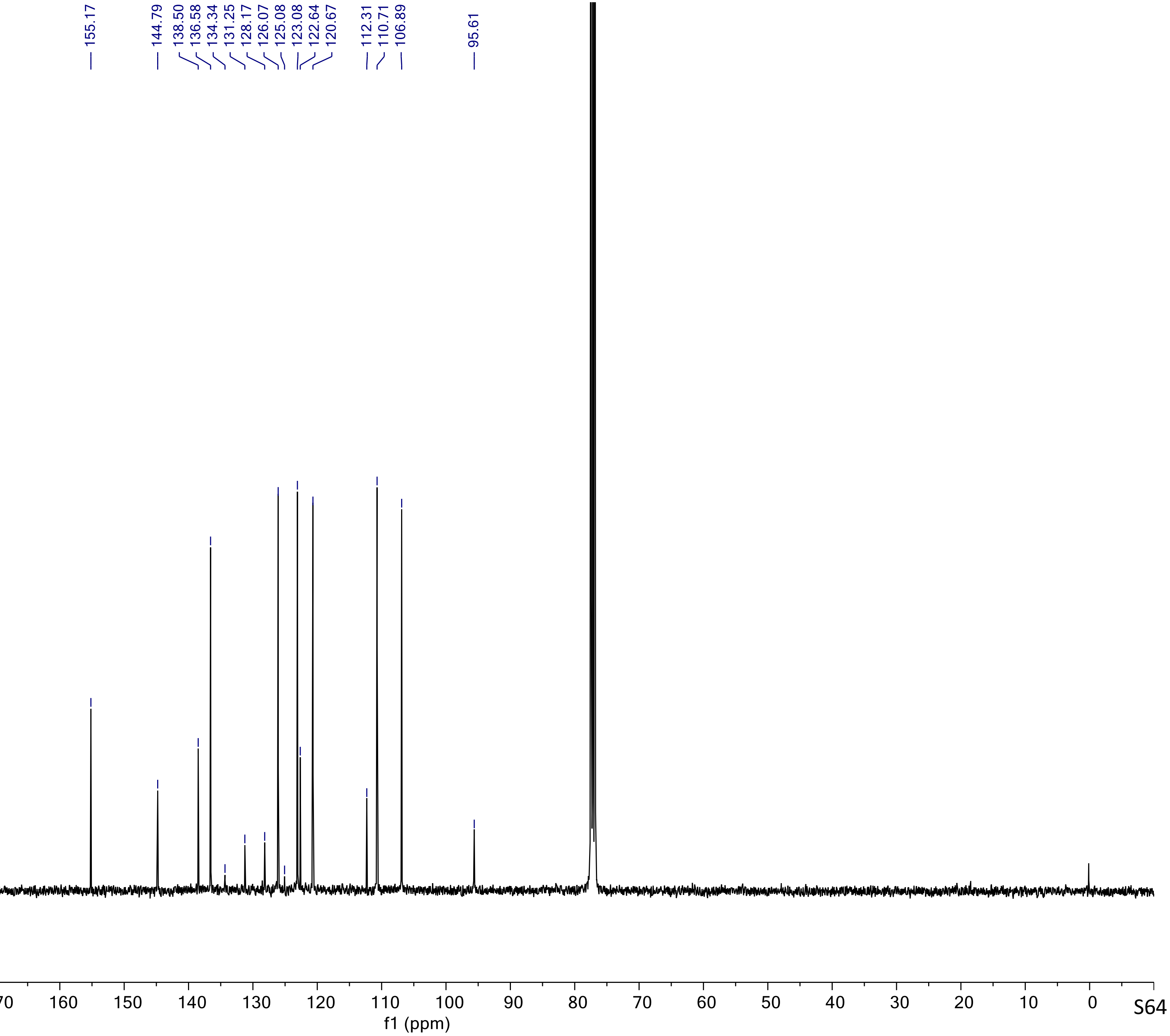

^{19}F NMR (376 MHz, $CDCl_3$)

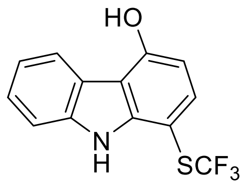

10d

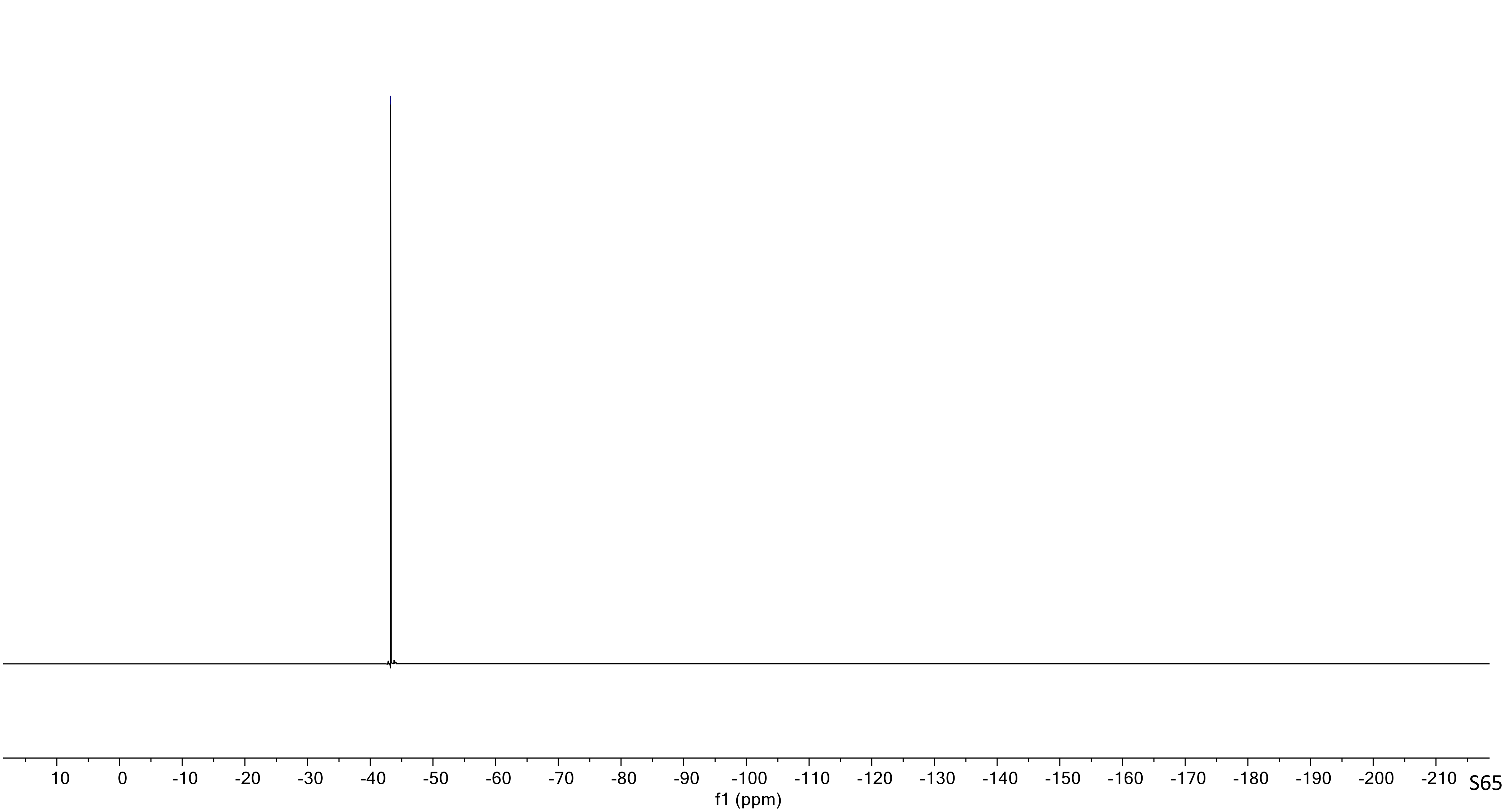

^{1}H NMR (400 MHz, $CDCl_3$)

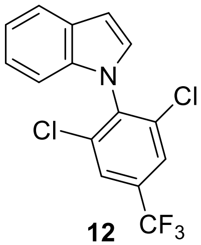

12

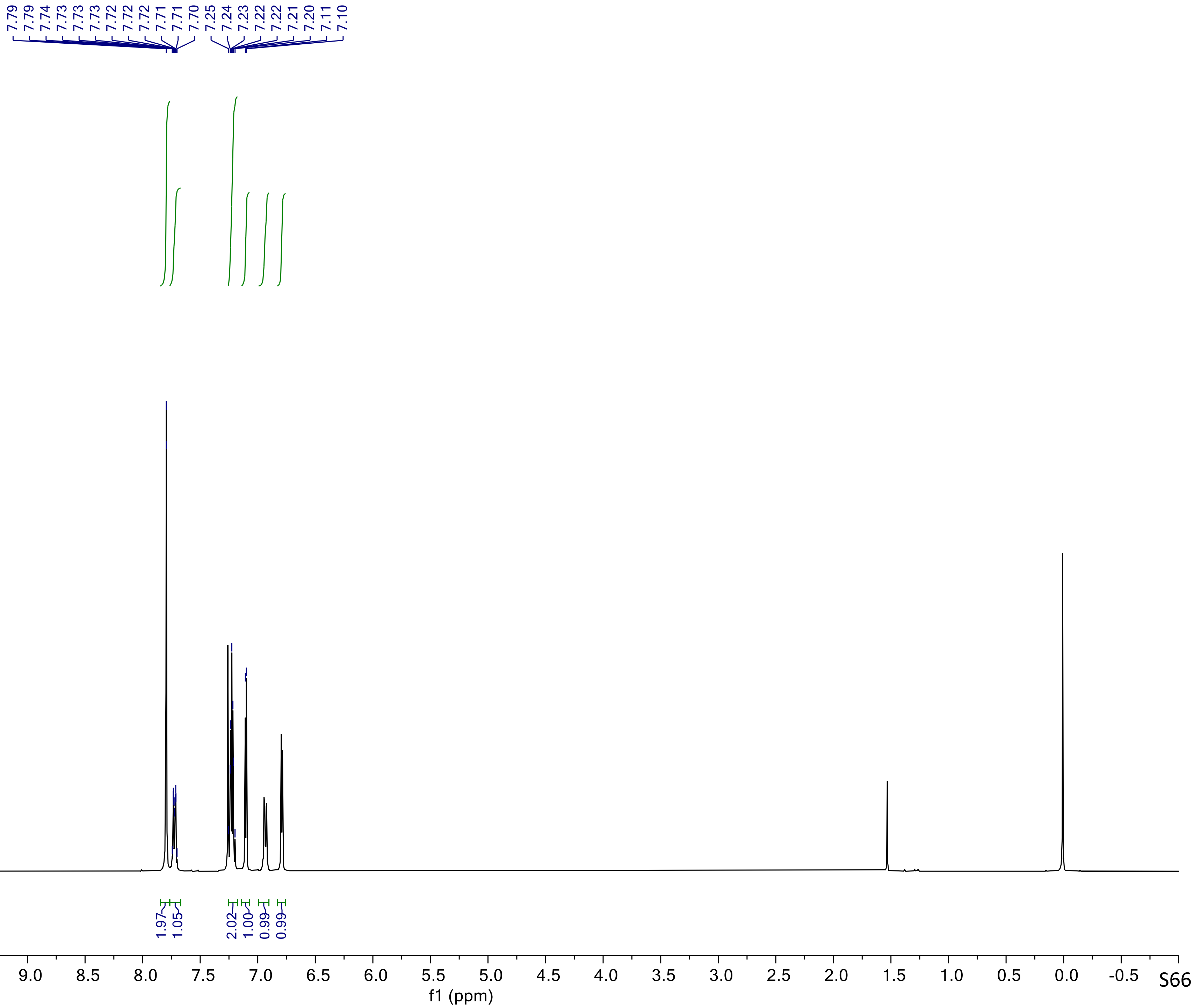

$^{13}C\{^{1}H\}$ NMR (101 MHz, $CDCl_3$)

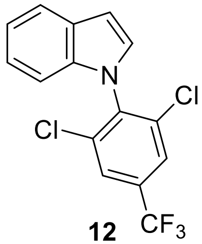

12

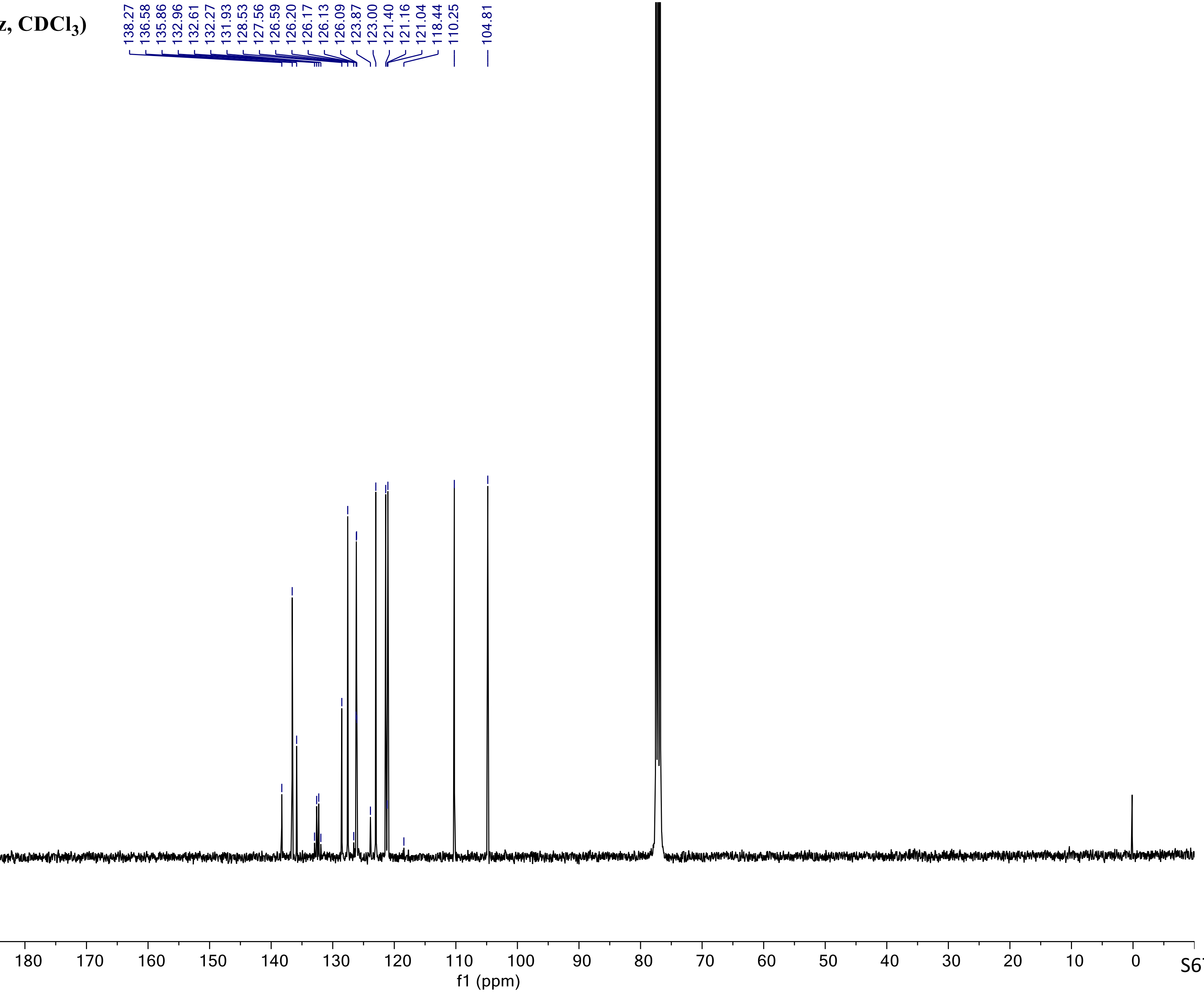

^{19}F NMR (376 MHz, $CDCl_3$)

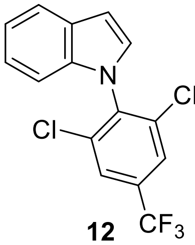

12

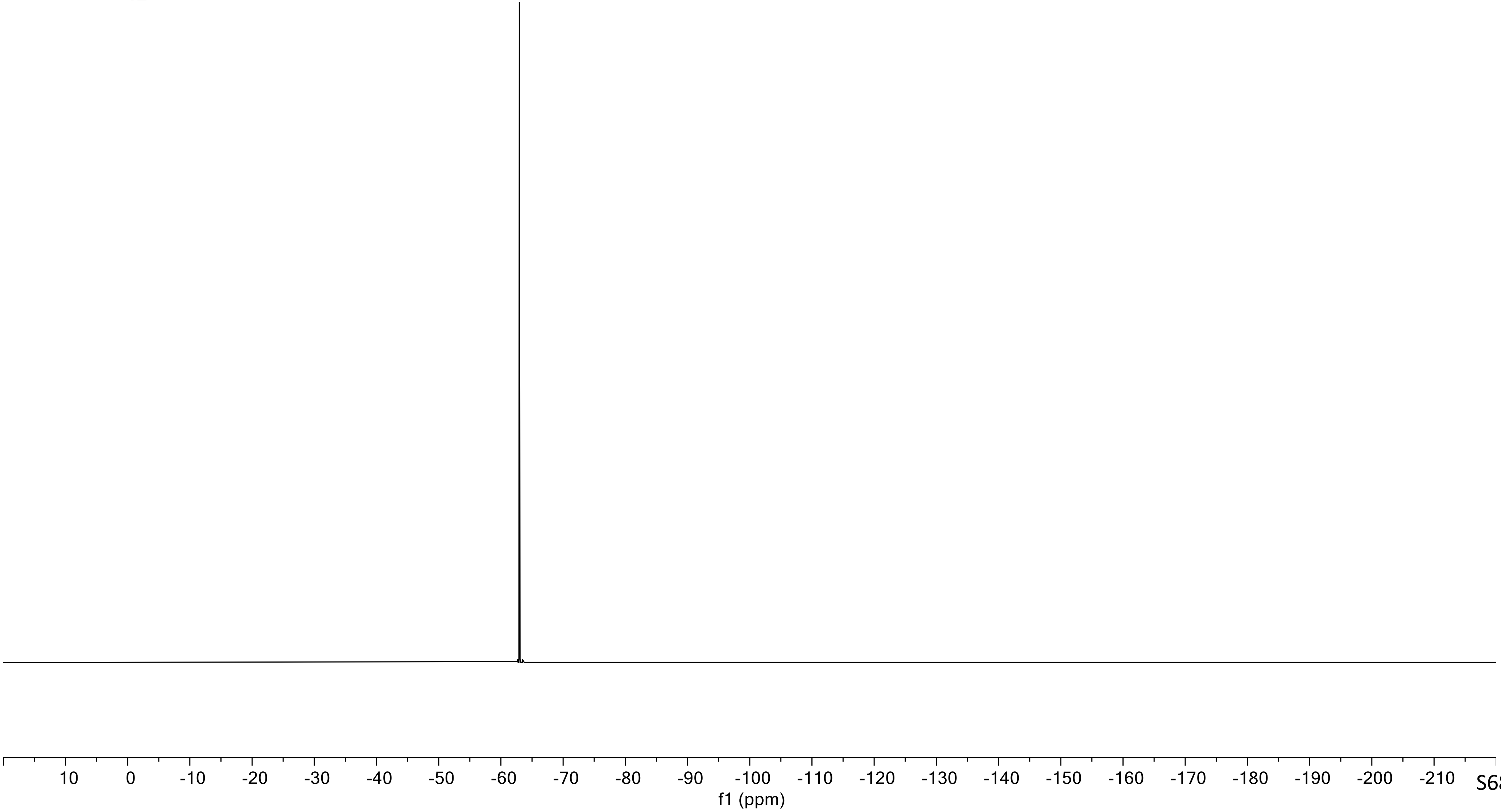

^{1}H NMR (400 MHz, $CDCl_3$)

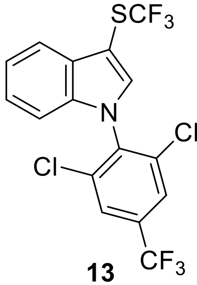

13

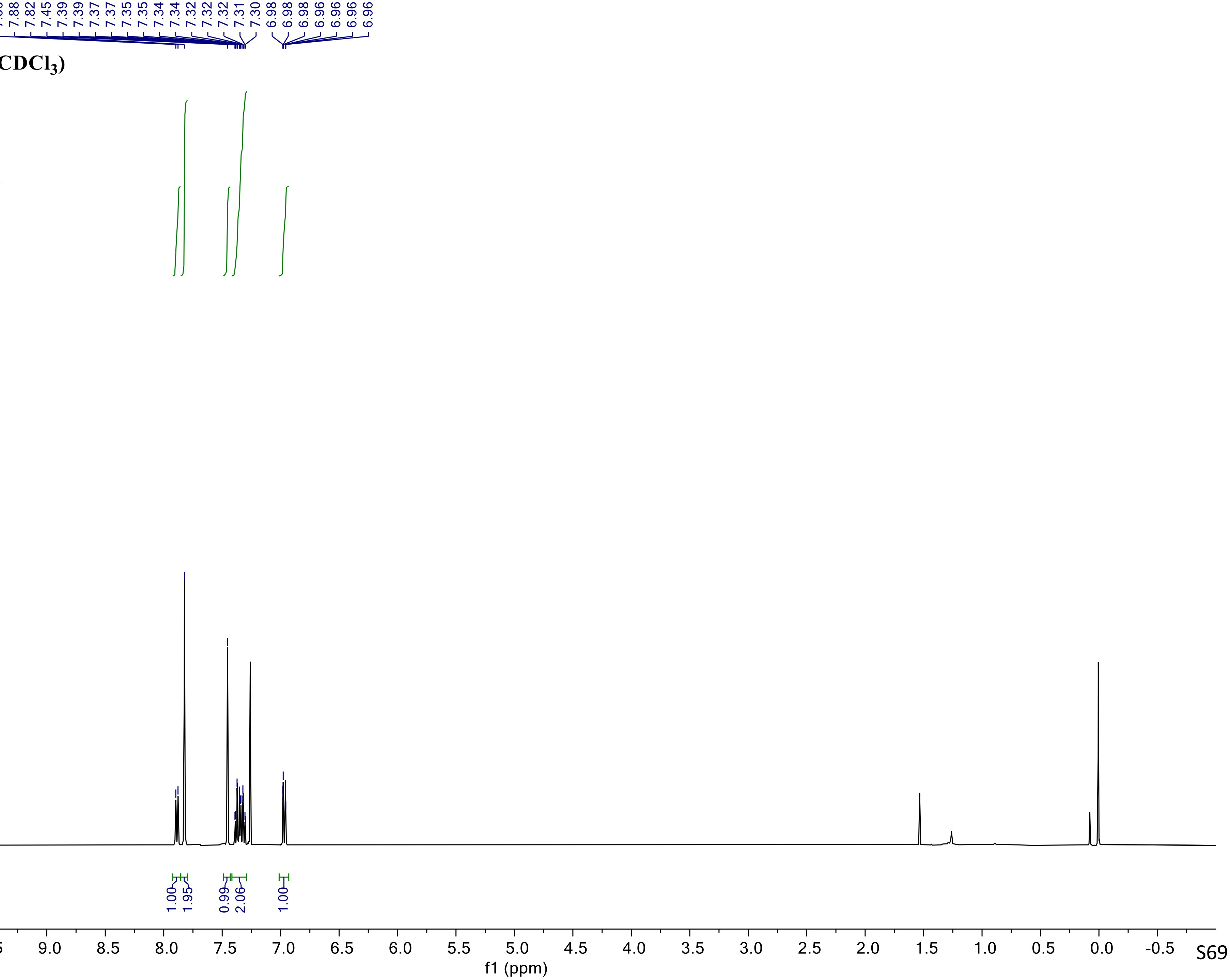

$^{13}C\{^{1}H\}$ NMR (101 MHz, $CDCl_3$)

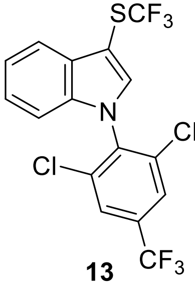

13

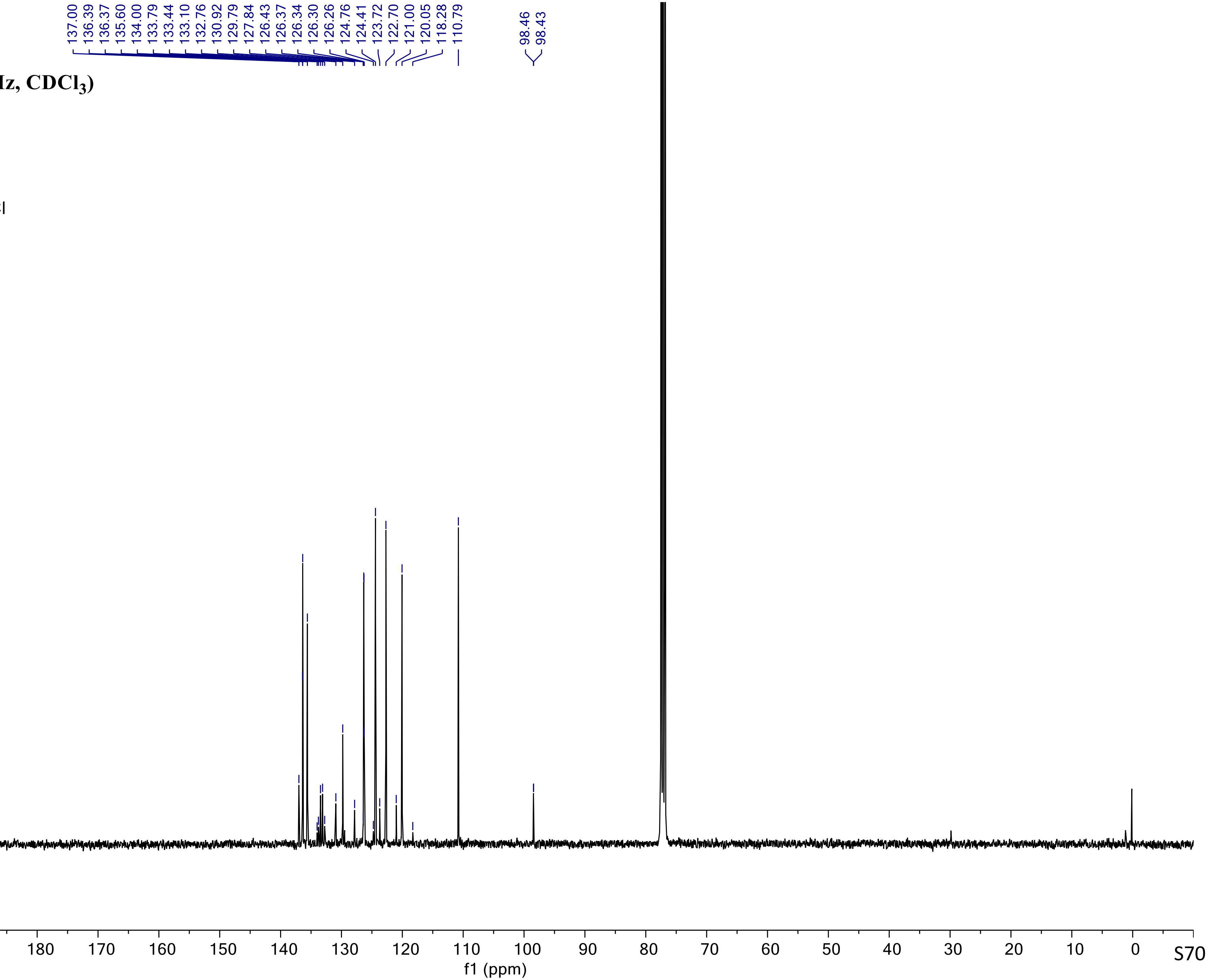

^{19}F NMR (376 MHz, $CDCl_3$)

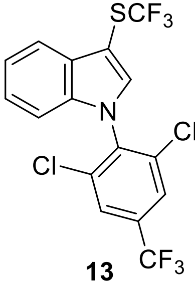

13

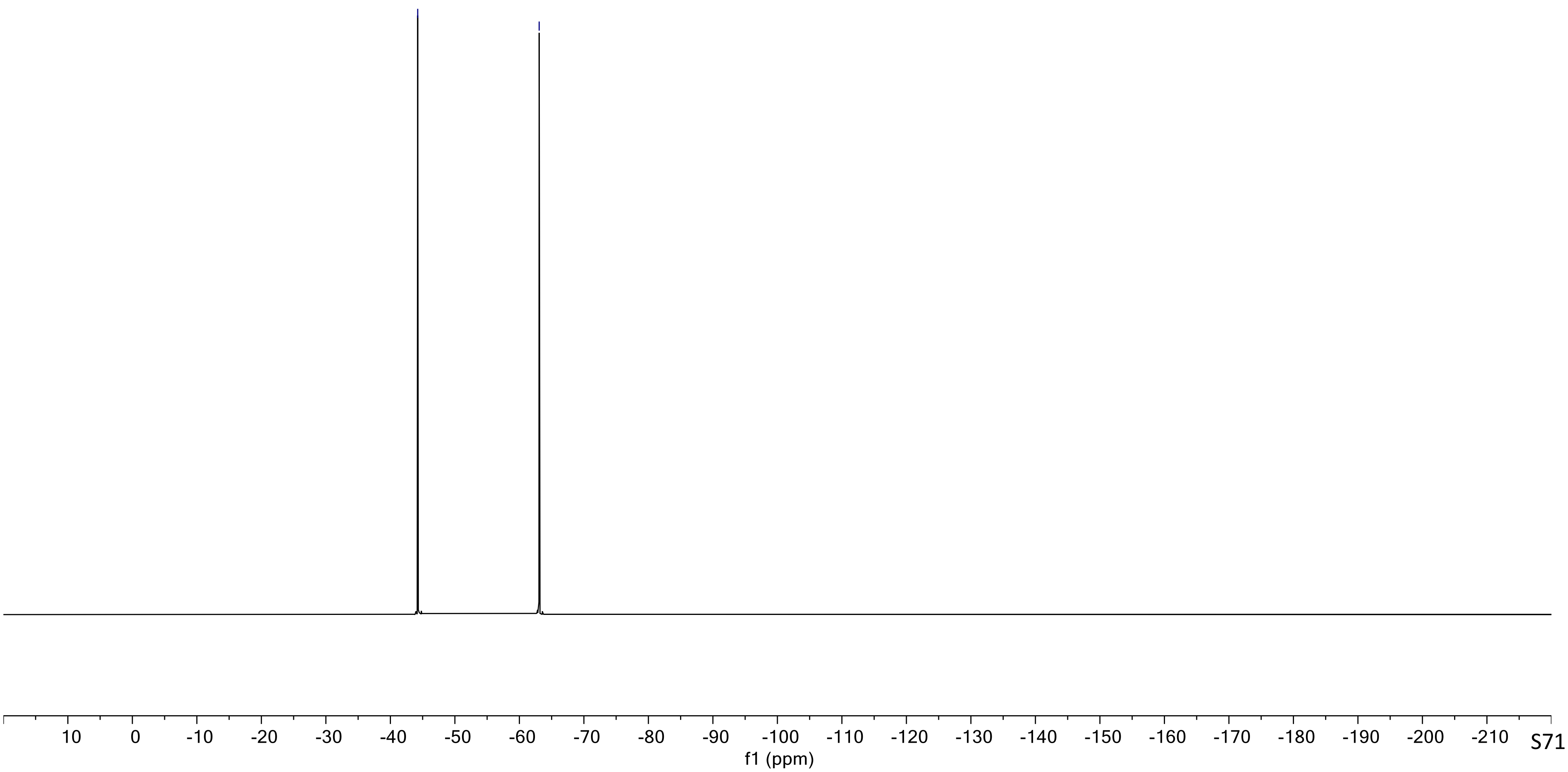

Supplement: Supplementary file 1 — jo3c02571_si_001.pdf [file jo3c02571_si_001.pdf]
